# Pacific tsunami records suggest similar rupture locations of two great Kamchatka earthquakes only 73 years apart

**DOI:** 10.1126/sciadv.aee5294

**Published:** 2026-09-23

**Authors:** Matías Carvajal, Kelin Wang, Alejandra Gubler, Tianhaozhe Sun, César Araya

**Affiliations:** ^1^ Instituto de Geografía, Pontificia Universidad Católica de Valparaíso, Valparaíso, Chile.; ^2^ Instituto Milenio de Oceanografía, Universidad de Concepción, Concepción, Chile.; ^3^ Pacific Geoscience Centre, Geological Survey of Canada, Natural Resources Canada, Sidney, British Columbia, Canada.; ^4^ School of Earth and Ocean Science, University of Victoria, Victoria, British Columbia, Canada.; ^5^ Instituto de Geografía, Pontificia Universidad Católica de Chile, Santiago, Chile.

## Abstract

Whether seismic slip can far exceed the slip budget of a subduction megathrust over a short time has important implications to earthquake physics and hazards. The first modern test comes from two great Kamchatka earthquakes of moment magnitude 8.8 to 9.0 in 1952 and 2025. Here, we use tide gauge tsunami records across the Pacific to determine whether the two events ruptured the same or complementary fault areas. We extract records of the 1952 tsunami from analog marigrams at 52 tide gauge stations and minimize their time uncertainties by matching hindcast tides. The 1952 tsunami waveforms and travel times are remarkably similar to those of the 2025 event. Tsunami modeling shows that a range of relative rupture locations can produce the waveform similarity, but only nearly colocated ruptures can explain the travel time similarity. On the basis of conventional rationale, the 2025 earthquake consumed more than twice the megathrust slip deficit accrued since 1952. We explain the conundrum by fast energy accumulation in a viscoelastic Earth.

## INTRODUCTION

**Fig. 1.**
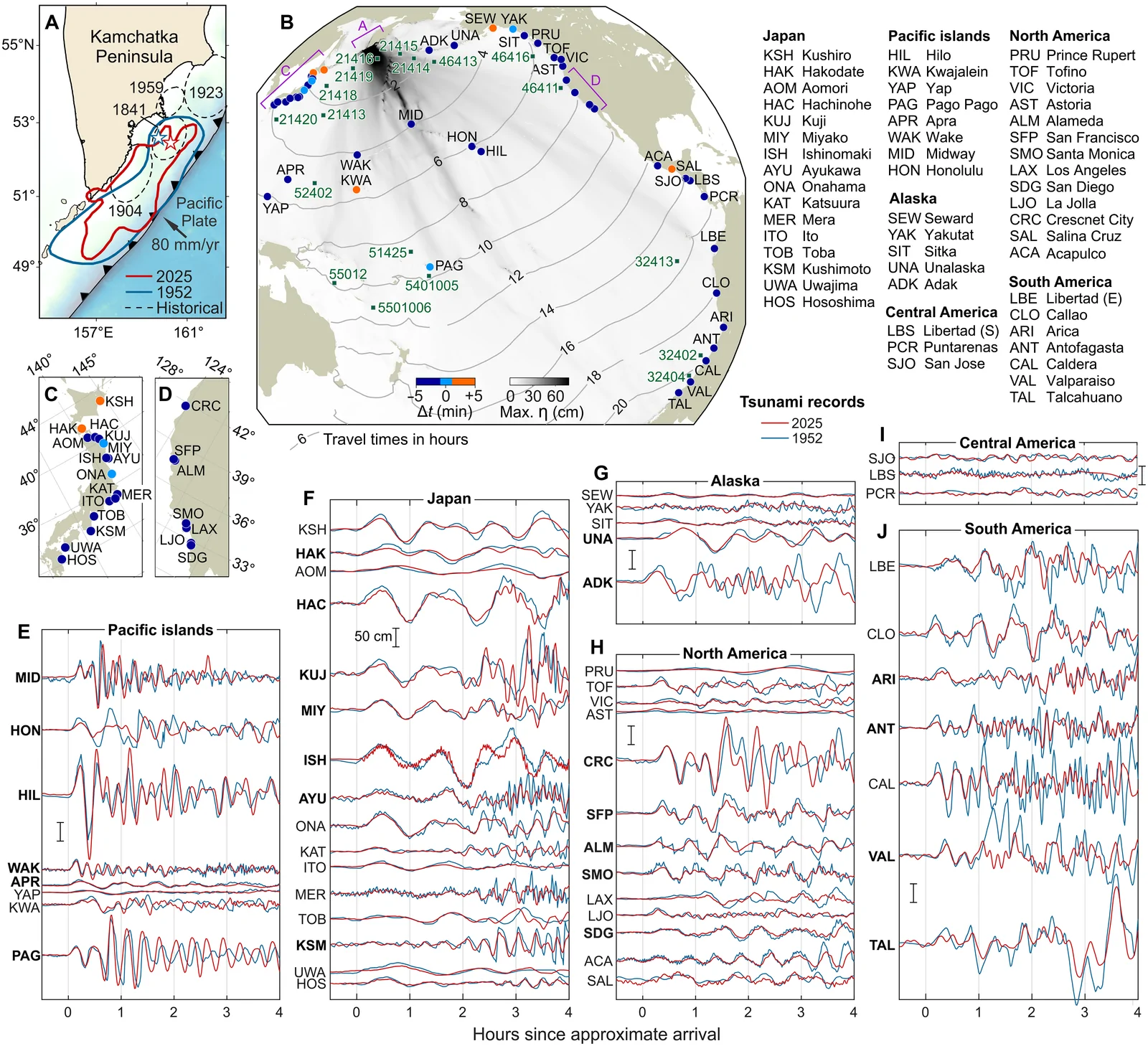
Pacific tsunami records of the great 2025 and 1952 Kamchatka earthquakes. (**A**) Tectonic setting of southern Kamchatka. Outlined are the 2-m slip contour of the 2025 rupture (red) (*13*), 1952 aftershock zone (blue) (*15*), and approximate rupture areas of historical megathrust earthquakes (dashed). Stars indicate 2025 and 1952 epicenters. Bathymetry is from the GEBCO_2019 Grid (*49*). yr, year. (**B**) The 52 tide gauge stations color coded by $\Delta t=T_{\text{1952}}-T_{\text{2025}}$, where $T_{\text{1952}}$ and $T_{\text{2025}}$ are travel times of the tsunami waves to each station in 1952 and 2025, respectively. DART stations used in Fig. 3F are shown with green squares and labeled with station names. Maximum tsunami elevation and travel times in hours are calculated using model Ref33 (Fig. 3B). (**C** and **D**) Close-up view of two areas marked in (B). (**E** to **J**) Tsunami waveforms at the 52 stations. −Δ*t* is added to the 1952 records for easy comparison with the 2025 records. Names of the stations used in tsunami modeling are shown in boldface.

Great earthquakes usually fill “gap” areas of the culprit fault that are in a locked state and have not ruptured in recent large earthquakes (*1*–*8*). Because seismic slip over multiple earthquakes should not exceed the slip budget of the fault, the expectation of “gap filling” is an important consideration in earthquake preparedness. However, two great megathrust earthquakes off southern Kamchatka call this expectation into question (Fig. 1A). Here, the Pacific plate subducts at a rate of 80 mm/year, generating many megathrust earthquakes (Fig. 1A) (*9*, *10*). South of 53°N, where seismic rupture tends to be larger and less frequent than farther north, the recurrence interval of great earthquakes is about 140 to 200 years (*9*, *11*, *12*). The 2025 moment magnitude (*M*_w_) = 8.8 earthquake and the 1952 *M*_w_ = 8.8 to 9.0 earthquake both occurred in this area but only 73 years apart. Because fault slip in the 2025 earthquake far exceeds the 6-m deficit of the Kamchatka megathrust accrued over 73 years (*13*, *14*), one could expect the 2025 rupture to have filled a gap left behind by the 1952 rupture. However, the two events appear to be in unexpectedly close spatial proximity (Fig. 1A), with epicenters located next to each other, aftershocks distributed in roughly the same area (*13*, *15*), and comparable tsunami heights along the Kamchatka and nearby coasts (*16*, *17*). For understanding hazards and fault dynamics, it is important to scrutinize to what degree their rupture zones overlapped and whether the scenario of gap filling is feasible.

The 2025 rupture is constrained by ample modern seismic-geodetic records (*13*) and tsunami observations from space (*18*) (Fig. 1A), but until recently, the location and distribution of the 1952 rupture were inferred only from limited tsunami observations (*19*, *20*). In the most comprehensive study of the 1952 event (*19*), using locally observed tsunami heights, several rupture scenarios ranging from a single patch south of 52°N to more complex slip extending as far north as 54°N explain the local tsunami observations equally well. The poor knowledge of the 1952 rupture zone poses greatest challenge to investigating the overlapping versus gap-filling relationship between the two events. Recently, Fujii and Satake (*21*) derived rupture models for both earthquakes by inverting tsunami waveforms from 37 tide gauge locations across the Pacific Ocean. They reported a high-slip patch of the 1952 rupture ∼10 km shallower than that of the 2025 event and interpreted this difference as evidence for a form of gap filling. However, this interpretation assumes that waveform fitting alone can robustly resolve the relative location of the rupture zones.

Here, we consider both tsunami travel times and waveforms to study the relative location between the 1952 and 2025 ruptures. We search, gather, digitize, and analyze tide gauge records of the 1952 tsunami at 52 stations across the Pacific in Japan (=16), Alaska (=5), North America (=13), Central America (=3), South America (=7), and Pacific islands (=8) (Fig. 1, B to D, and table S1). A subset of these stations was also used by Fujii and Satake (*21*) (table S1). The 1952 tsunami waveforms and travel times at the 52 stations are remarkably similar to those of the 2025 event (Fig. 1). We will show that tsunami waveform similarity between the 1952 and 2025 events can be reproduced by slip models spanning a range of relative rupture locations but that the observed tsunami travel time similarity requires the two rupture zones to be nearly colocated.

## RESULTS

### Correcting tsunami waveforms and travel times from analog marigrams

**Fig. 2.**
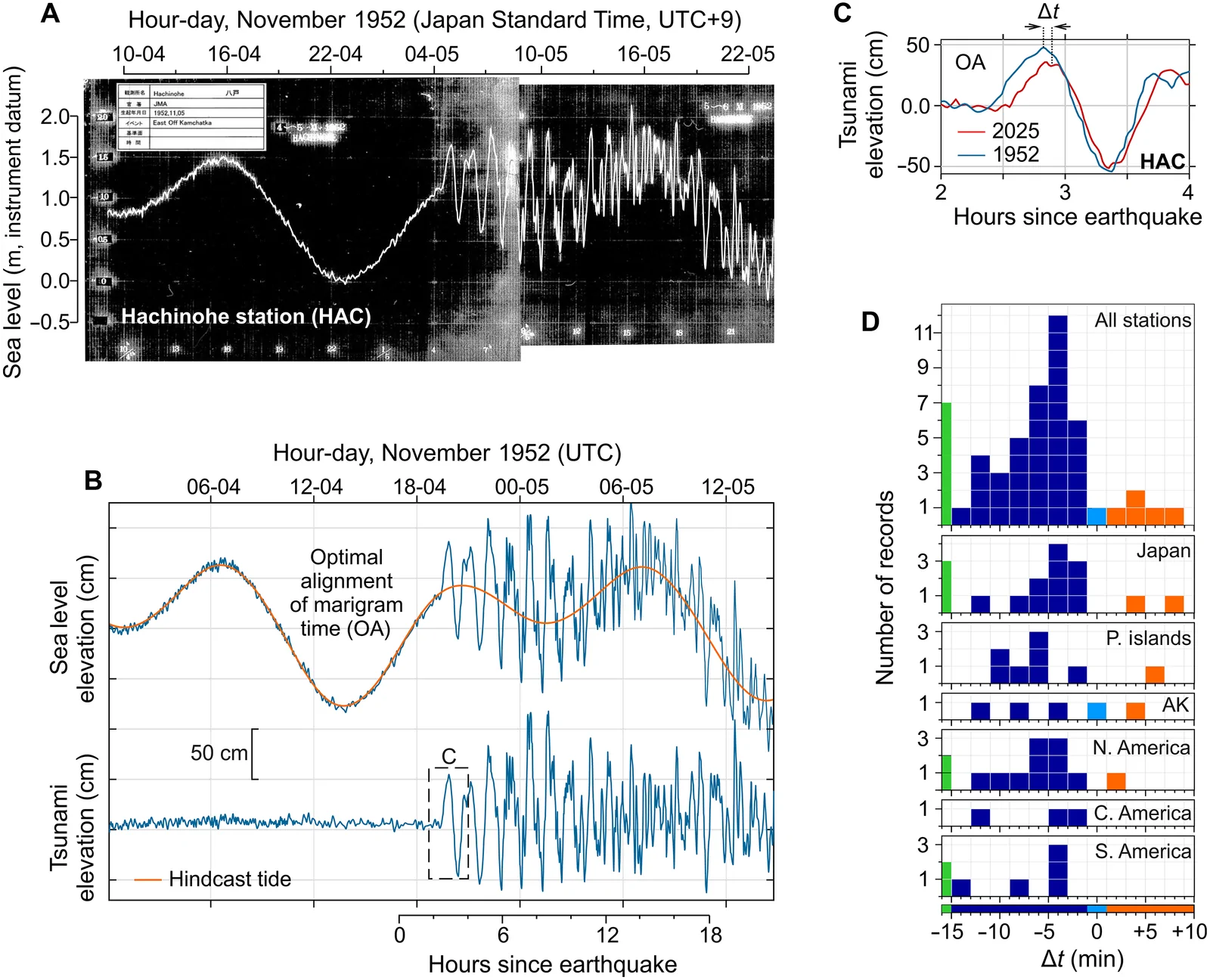
Correction procedure of 1952 marigrams and travel time difference between the 1952 and 2025 tsunamis. (**A**) Example of a raw analog paper marigram [Hachinohe station (HAC)]. (**B**) Corrected marigram at HAC by matching hindcast tide (top) and resultant tsunami waveforms (bottom). (**C**) Tsunami travel time difference ($\Delta t=T_{\text{1952}}-T_{\text{2025}}$) estimated at HAC. (**D**) Histograms of Δt for all the stations and by region. Green indicates Δt < −15 min.

Today, marigrams (tide gauge water level time series) are digitally recorded, such that the 2025 tsunami records shown in Fig. 1 (E to J) are accurate in both absolute time and amplitude. In contrast, the 1952 marigrams are analog paper traces and must be digitized. The digitized records suffer from instrument clock errors, digitization inaccuracies, and, in some cases, missing amplitude scales (Fig. 2A). These issues led to considerable challenges in properly distilling and making use of the travel time information. Therefore, Fujii and Satake (*21*) allowed the observed waveforms to be shifted in time as much as needed to optimize waveform fit in their inversion procedure. However, by aligning the digitized records with highly accurate hindcast tides (Fig. 2B), uncertainties can be greatly reduced (*22*). In this procedure, time, trend, offset, and amplitude are adjusted either manually or with an automated correlation algorithm until optimal alignment (OA) is visually identified. Clear tsunami records are obtained by subtracting the tidal signals (Fig. 2B). Amplitude uncertainties in the resultant records are negligibly small (*22*). We estimate that time uncertainties are typically less than 10 min. At most stations, with HAC being an excellent example, alignment with the hindcast tides is confident within several minutes (fig. S1). However, at a few stations, the records are too short to allow unambiguous matching with the tidal cycles, giving rise to errors in OA as large as 50 min (fig. S2). In rare cases, large uncertainties of up to ±25 min can be caused by desynchronization between the recording drum and clock (fig. S3) (*23*). If a human operator had timely marked the desynchronization, such as at MID, corrections can be made today (fig. S3).

At 45 of the 52 stations, the travel time difference between the two tsunamis is small, mostly within 10 min, and all are within 25 min (Fig. 2D). Most critical for understanding the relationship between the two rupture zones, the 1952 travel time $T_{1952}$ appears to be not longer, or even slightly shorter than, that of 2025 $T_{2025}$ (i.e., $∆t=T_{1952}-T_{2025}\leq 0$) at the vast majority of the stations (Figs. 1B and 2D and table S1). Arguably, ∆t for most stations (Fig. 2D) is within the 10-min typical time uncertainties and could be considered zero. However, its systematic occurrence in all azimuths (Fig. 2D) is unlikely an artifact because time errors in the corrected records should be random. In Fig. 1 (E to J), −∆t is added to the 1952 OA time to allow a tidy display of the records.

### Learning from 2025 and 1952 tsunami data with basic slip models

**Fig. 3.**
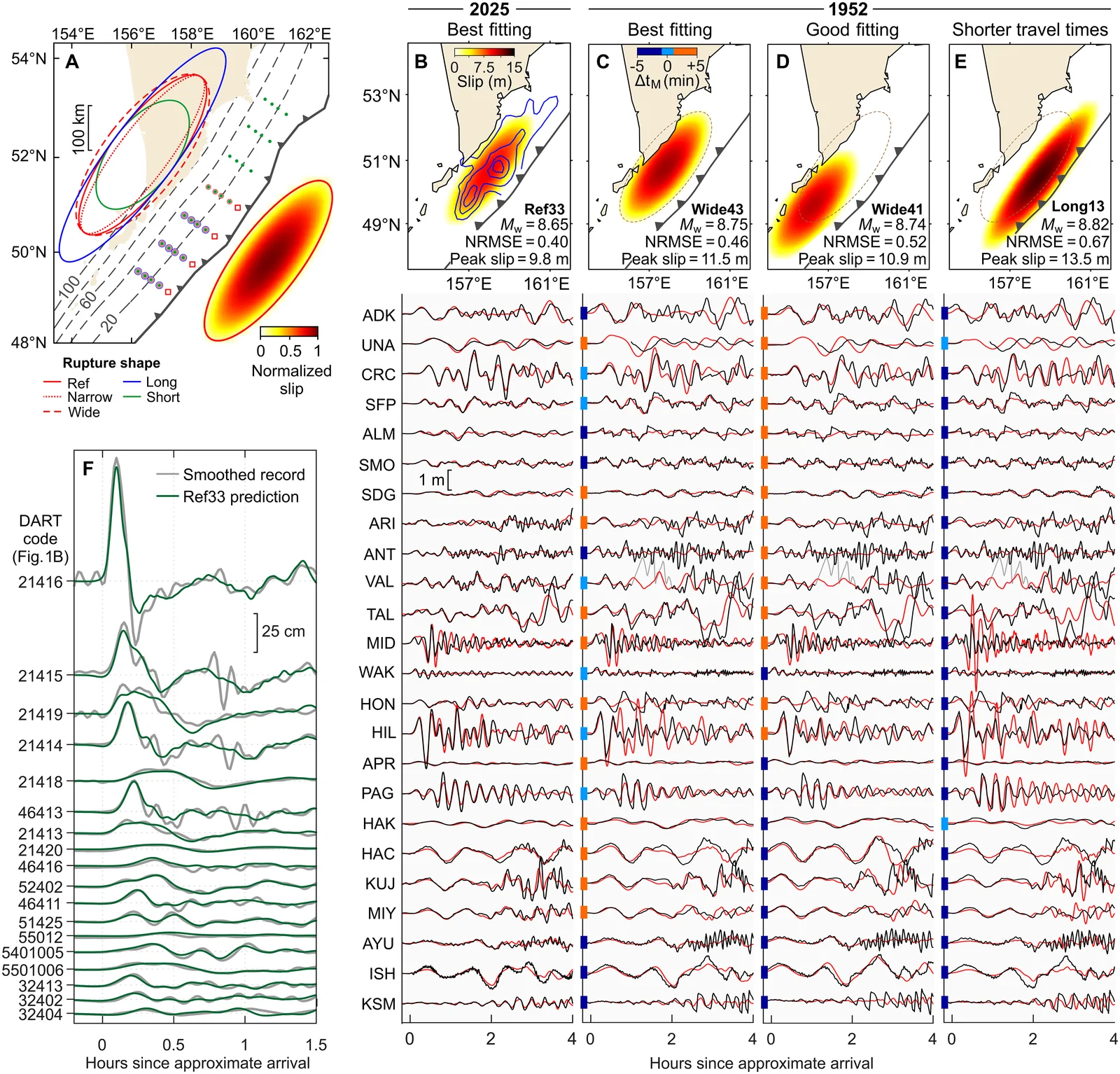
Exploring Pacific tsunami sensitivity to rupture location with basic slip models. (**A**) Definition of the basic models. Each shape group is shown to scale by the contour of its 10% peak slip. Slip distributions in all models are similar to the shown example. The Ref, Narrow, and Wide groups share a 4 × 4 centroid grid (red), and Short or Long has a 4 × 7 (green) or 4 × 3 (blue) grid, respectively. Squares mark TB1 to TB4 trench-breaching models from southwest to northeast. Dashed gray lines show depth (in km) contours of the subduction interface (*25*). (**B**) Preferred 2025 basic model and its predicted tsunami waveforms (red) compared with tide gauge records (black). Blue lines in map are 2-, 6-, 10-, and 12-m slip contours of the best model of Liu *et al.* (*13*). (**C** to **E**) Similar to (B) but for three 1952 basic models. Rectangles at the beginning of each waveform are color coded according to the model-predicted travel time difference ($∆t_{M}$). The VAL 1952 gray segment likely reflects a recording error. (**F**) Tsunami elevation predicted by Ref33 in (B) at DART stations shown in Fig. 1B in comparison with 1-min smoothed records.

In the far field, the amplitude of a tsunami due to a megathrust earthquake is determined largely by the *M*_w_ of the earthquake and the size, location, and orientation of its rupture zone (*22*). However, the travel time of the tsunami is determined only by the location. Bearing in mind that complexity in the slip distribution can also affect far-field tsunami waveforms (*24*), we begin by using simple models to explore what can be learned, primarily about rupture location, from the tide gauge data (Fig. 1). For each of the two earthquakes, we numerically model tsunamis for 92 basic slip models (see Materials and Methods). We consider five groups of elliptical rupture zones: Ref, Narrow, Wide, Short, and Long as defined in Fig. 3A; apply each group to a grid of centroid locations (Fig. 3A); and name the models by their dip-strike position in the grid. For example, Ref23 is the Ref model on the second row from the trench side and third column from the southwest. An additional group named TB1 to TB4 is placed at the four near-trench locations (Fig. 3A). They are Narrow slip distributions truncated by the trench, representing the possibility of moderate trench-breaching rupture. Accurate modeling of tsunami waveforms and travel times at tide gauge locations requires bathymetric data with finer resolution than provided by coarse global datasets (*21*). Because these data are inaccessible for some regions, we assemble detailed bathymetric grids to conduct detailed modeling at 24 of the 52 stations in this investigation. The 24 stations still provide excellent azimuth and distance coverage for inferring rupture location at Kamchatka (Fig. 1). The method of tsunami modeling follows Carvajal *et al.* (*22*) (see Materials and Methods).

For each basic slip model, we adjust the slip magnitude to optimize agreement with the observed tsunami waveforms simultaneously at all the stations. In fitting waveforms, we also need to shift the tide gauge records in time but in different ways for the two earthquakes. For 2025, the shift is up to only ±3 min to accommodate finite rupture propagation time and minor bathymetric inaccuracies. Under this tight time-shift constraint, wrong location of the rupture results in poor waveform fit, reflected in a higher normalized root mean square error (NRMSE) (see Materials and Methods). For 1952, we impose no limit on the shift because (i) uncertainties in $T_{1952}$ are much larger, and (ii) as will be subsequently discussed, we wish to examine $∆t_{M}=T_{M1952}-T_{2025}$ to gain information about the location of the 1952 rupture relative to 2025, where $T_{M1952}$ is model-predicted 1952 travel time. Another factor of consideration is that the direction of rupture propagation in a megathrust earthquake slightly affects the directivity of the beam of tsunami energy, which, in turn, affects the amplitude and travel time of far-field tsunami waves (*25*). However, both the 2025 and 1952 ruptures started from a similar location and propagated in the same southwest direction (Fig. 1A) (*13*, *26*). Therefore, in studying the relative locations of the two ruptures, the directivity effect can be ignored, and the use of static rupture models is justified. Tsunami waveforms predicted by all the basic slip models and their comparison with the tide gauge records are shown in figs. S5 and S6 for the 2025 and 1952 events, respectively. Some examples are shown in Fig. 3 (B to E).

Results from the basic models show that the observed tsunami travel times provide critical constraints for the rupture locations of the Kamchatka earthquakes, but the waveforms alone are inadequate for this purpose. This is best illustrated by the 2025 event, for which “ground truth” is provided by the rupture location of Liu *et al.* (*13*) constrained by seismic-geodetic observations. Our basic models that fit the 2025 tsunami observations (fig. S5) are centered around 50° to 51°N, consistent with the preferred solution of Liu *et al.* (*13*), despite their wide range of rupture shape (e.g., Short, Long, etc.) and peak slip (9 to 18 m). One of these models (*27*) is shown in Fig. 3B, exhibiting excellent agreement with Pacific DART records for the 2025 event (Fig. 3F). The good agreement in location with the seismic-geodetic model owes to the well-determined tsunami travel times, with allowable time shifts limited to only ±3 min. Model ruptures too far from the optimal location fail to reproduce the observed travel times, leading to poor waveform alignment and hence unacceptable fits.

For 1952, because larger time shifts are allowed to accommodate greater travel time uncertainties, a greater number of basic models can fit the observed waveforms (fig. S6). However, although the absolute location of the 1952 rupture is difficult to determine, its location relative to 2025 can be rather accurately constrained by the propensity for ∆t≤0 (Fig. 2D) in all azimuths. Inferring the relative location of these two events from their tsunami travel time differences is akin to determining the relative location of two earthquakes from their seismic-wave travel time differences (*28*). Each basic model for 1952 yields a $∆t_{M}$ for each station. In Fig. 3 (C to E), $∆t_{M}$ is represented by the color of the rectangle at the beginning of each waveform, whereas, in fig. S6, it is represented by the color of the station label. These results strongly demonstrate that good waveform agreement alone is not sufficient to constrain relative rupture location. Most of the basic models that fit waveforms very well predict $∆t_{M}>0$ in some azimuths, inconsistent with observations. For example, Wide43 provides the best overall fit to the waveforms (NRMSE = 0.46) but predicts $∆t_{M}>0$ at most Pacific Islands stations because its deeper location places it farther from those stations than the shallower 2025 rupture (Fig. 3C). Shifting the rupture 200 km southwest (Wide41 in Fig. 3D) only slightly degrades the waveform fit (NRMSE = 0.52) but predicts $∆t_{M}>0$ at northeastern stations instead (Fig. 3D). Obviously, if $T_{M1952}$ is to be shorter than $T_{2025}$ in all azimuths, assumed tsunamigenic slip should extend somewhat beyond the 2025 rupture zone in all directions except downdip. One example is Long13 (Fig. 3E), but it fits the waveforms less well than many other models (NRMSE = 0.67).

### More nuanced estimates of the two rupture zones

The basic models yield important insights but are obviously too simplistic and tend to underrepresent the magnitude of either earthquake. None of the optimal 1952 basic models in Fig. 3 can explain observed waveforms and travel times at the same time. It is clear that even the far-field tsunami records, which are not very sensitive to source details, require nuances beyond the simple bell-shape slip.

**Fig. 4.**
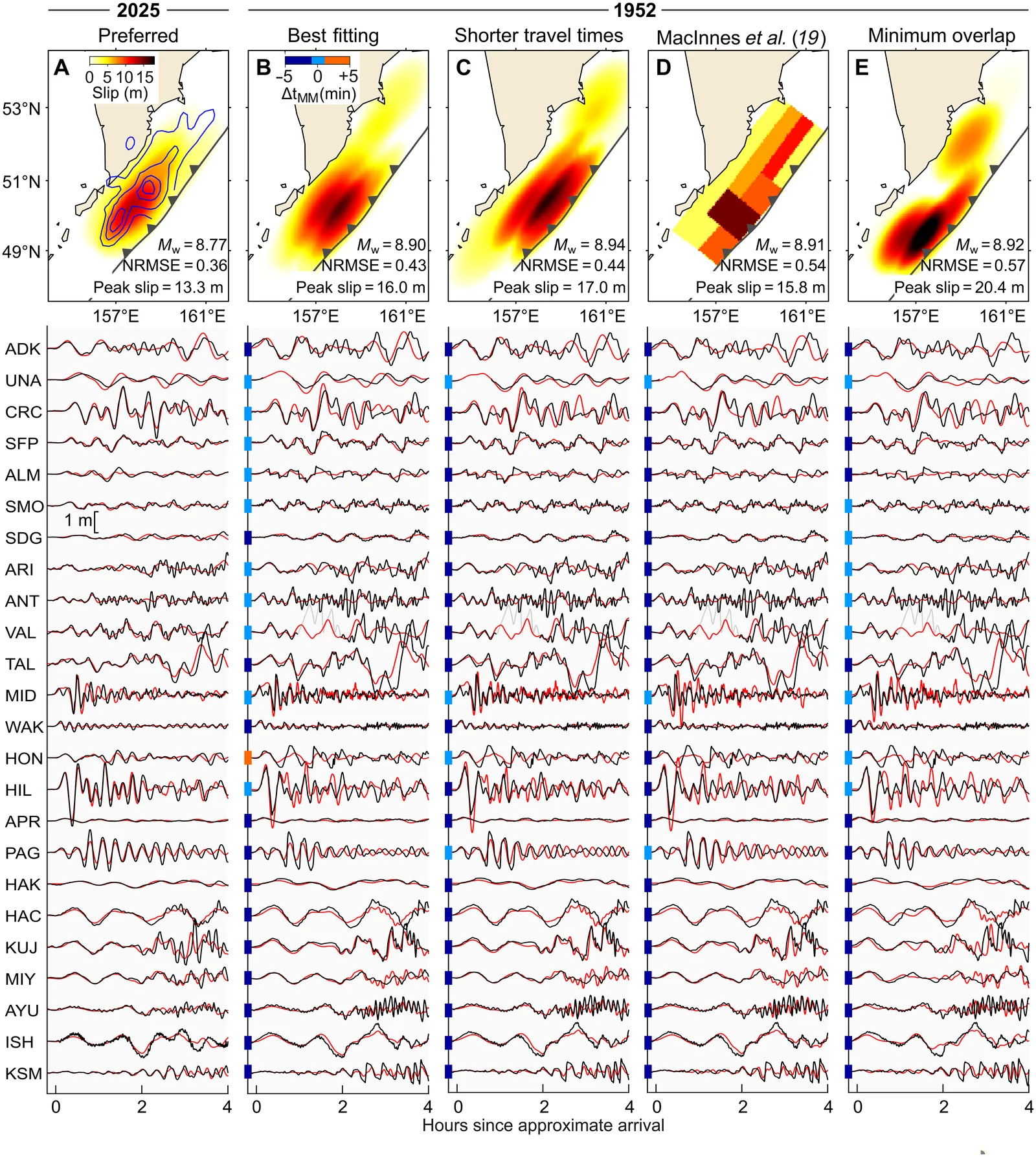
Inferred slip models for the great 2025 and 1952 Kamchatka earthquakes. (**A**) Preferred 2025 slip model as a weighted average of basic models, with waveform comparisons as in Fig. 3. Blue lines in map are 2-, 6-, 10-, and 12-m slip contours of the best model of Liu *et al.* (*13*). (**B** and **C**) Weighted-average 1952 models derived with time-shift windows of ±10 min and −10 to −3 min, respectively (see text). (**D**) One of the slip models of MacInnes *et al.* (*19*) based on local tsunami data. (**E**) A weighted-average 1952 model that is forced to minimize overlap with the 2025 rupture in (A) but still reasonably fits tide gauge data. The color of the rectangles in (B) to (E) is defined in (B). Contributing models for (A) to (C) and (E) are shown in fig. S7.

Therefore, we derive weighted averages of the 92 basic models to explain better the tsunami data. To determine the weights of such an average, we use a procedure similar to linear inversion of tsunami data (see Materials and Methods). A fault slip model derived from inversion is a weighted average of point or subfault sources distributed along the megathrust, and the inversion would minimize the difference between their tsunami-wave Green’s functions and tsunami observations. In our procedure, the tsunami wave fields predicted by our 92 slip models play the role of the Green’s functions. In deriving the 2025 average, we allow a small shift of tide gauge records from $T_{2025}$ up to ±3 min, similar to the basic models. In deriving the 1952 average, we invert 1952 tsunami records aligned with the 2025 records exactly as shown in Fig. 1 (E to J) but with two time-shift options. First, considering that the ∆t values (Fig. 2D) are mostly within typical time uncertainties of 10 min, we assume ∆t=0 on average but allow the records to shift up to ±10 min. Second, considering the tendency for ∆t to fall between −3 and −10 min (Fig. 2D), we impose a strong bias with an asymmetric time-shift window of −10 to −3 min to force $∆t_{\text{MM}}=T_{M1952}-T_{M2025}$ toward this window, where $T_{M2025}$ is travel time predicted by the model in Fig. 4A, which is very similar to $T_{2025}$.

The resultant weighted average models for 1952 (Fig. 4, B and C) are in almost the same location as that of 2025 (Fig. 4A) but cover a broader fault area and feature larger slip and a greater *M*_w_. Basic models that substantially contribute to the 2025 and 1952 weighted averages are summarized in fig. S7. Without an imposed travel-time bias, the model in Fig. 4B (NRMSE = 0.43) still predicts $∆t_{\text{MM}}\leq 0$ at nearly all the stations. With the imposed bias, the model in Fig. 4C (NRMSE = 0.44) predicts systematically $∆t_{\text{MM}}<0$ by placing more fault slip in all directions except downdip. Given uncertainties, we cannot tell which of the two models is better. For comparison, Fig. 4D shows the model most preferred by MacInnes *et al.* (*19*). This model was constrained only by local tsunami data, but it fits the far-field tsunami data unexpectedly well (NRMSE = 0.54) and bears some resemblance with the model in Fig. 4C.

Although the Pacific tsunami data can constrain the relative location of the ruptures, their inability to resolve slip details leads to some model ambiguity. For example, in Fig. 4 (A to C), the elliptical shape of high-slip patches inherited from contributing basic models should not be taken literally. To demonstrate the worst-case ambiguity for the 1952 rupture, we exclude the basic models that substantially overlap the best 2025 model shown in Fig. 4A by visual inspection and derive a weighted average of the remaining basic models. The basic models that contribute to this average are shown in fig. S7. The result still predicts acceptable tsunami waveforms (NRMSE = 0.57) and travel times but with a rupture zone that has less overlap with 2025 (Fig. 4E). Its >20-m peak slip probably would be inconsistent with the local tsunami observations used by MacInnes *et al.* (*19*).

## DISCUSSION

### Resolving the slip-budget conundrum

**Fig. 5.**
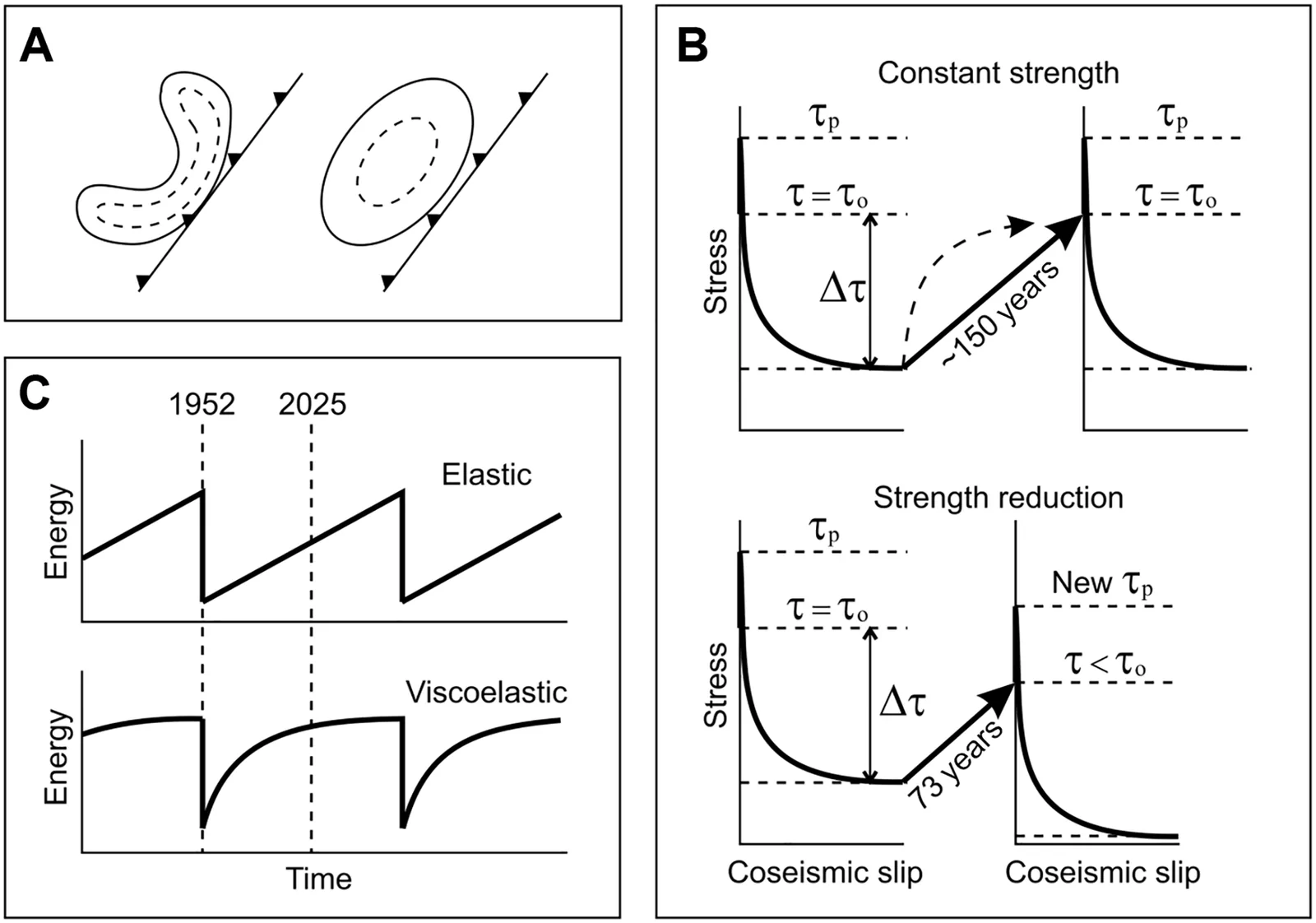
Schematic illustrations of three scenarios to resolve the slip-budget conundrum. (**A**) Hole filling. A low- or no-slip hole of one earthquake (left) hosts the peak slip of the next earthquake (right). (**B**) Fault strength reduction, illustrated using stress evolution of a fault patch during seismic slip [e.g., (*31*)]. At the propagating rupture front, fault stress τ is raised abruptly from the preearthquake value $\tau_{o}$ to yield stress (i.e., fault strength) $\tau_{p}$, followed by a sharp decrease due to dynamic weakening. The static stress drop ∆τ is the stress difference before and after the earthquake. Each panel shows two consecutive earthquakes separated by a period of stress buildup. Compared to the ideal situation (top), if $\tau_{p}$ endures a decrease after the first earthquake (bottom), the second earthquake may occur early, before τ returns to the previous value of $\tau_{o}$. (**C**) Early energy buildup (preferred), based on the conceptual model of Wang *et al.* (*27*). In an elastic Earth (top), the accumulation of elastic strain energy around a locked fault is linear with time and hence proportional to fault slip deficit. Long enough time is needed to build up sufficient slip deficit for a large rupture (vertical part of the curve). In viscoelastic Earth (bottom), energy accumulation is fastest right after the preceding earthquake (e.g., 1952), such that the next earthquake may occur earlier (e.g., 2025). Accordingly, fault stress increase is also faster early in the cycle, as illustrated by the dashed path in the top panel of (B).

A recurrence time as short as 73 years for two *M*_w_ 8.8 to 9.0 megathrust events is unprecedented in the instrumental era of seismology. There have been efforts to argue that the 1952 event might have ruptured predominantly either a deeper (*29*) or a shallower (*21*, *30*) part of the Kamchatka megathrust than did the 2025 event. While our findings demonstrate that the two rupture zones were almost colocated, they also demonstrate that the relative locations of the high-slip patches cannot be uniquely constrained by the Pacific tsunami records. If the 2025 rupture did not fill a gap left by the 1952 rupture, then it raises a conundrum about slip budget. Peak slip of either event (Fig. 4) greatly exceeds the 6-m total plate convergence over 73 years. Three possibilities can be entertained (Fig. 5). The first is “hole filling” (Fig. 5A), that is, a low- or no-slip “hole” in 1952 later accommodated peak slip in 2025. This interpretation is proposed in (*21*, *29*, *30*) and is represented by the model of Fig. 4E. However, this may be difficult to accomplish mechanically. If the hole had arrested the large seismic slip from all directions in 1952, then it would be the least likely place for the 2025 rupture to peak.

The second possibility is “fault strength reduction” (Fig. 5B). According to standard theory for earthquake dynamics (*31*, *32*), a key condition for rupture propagation is that fault stress $\tau_{o}$ just before the earthquake should not be too far below fault strength $\tau_{p}$. During the earthquake, the propagating rupture front would raise the stress from $\tau_{o}$ to $\tau_{p}$, and the ensuing dynamic weakening would lead to stress drop ∆τ. The gap-filling expectation is based on the recognition that it takes some time for tectonic loading to bring the postearthquake stress $\tau_{o}-∆\tau$ back to $\tau_{o}$, as schematically illustrated with an arrow showing stress buildup over ∼150 years in Fig. 5B. In this hypothetical ideal situation, if the time elapsed from the previous earthquake is short, then the fault stress is much lower than $\tau_{o}$ and hence too far below $\tau_{p}$. However, if somehow $\tau_{p}$ endured substantial decrease since 1952, then it would take less time such as 73 years for the fault stress to become close enough to the new $\tau_{p}$ to enable a rupture. Nonetheless, it is difficult to identify a geological process to reduce $\tau_{p}$ over such a large fault area during this time.

The third, and our preferred, possibility is “early energy buildup” (Fig. 5C). In a model of elastic Earth, slip deficit of a locked fault is a direct measure of energy buildup for the next earthquake (and fault stress increase toward $\tau_{o}$), which is linear with time. However, as explained by Wang *et al.* (*27*), in a viscoelastic Earth, the energy buildup (and fault stress increase) is the fastest right after the earthquake and slows down with time in the interseismic period. Although Wang *et al.* (*27*) focused on strike-slip faults, they showed that the same physical principle applies to subduction faults. A direct indicator of energy accumulation for future megathrust earthquakes is margin-normal shortening of the subduction zone forearc. In the postseismic to early-interseismic periods, the viscoelastic relaxation of mantle stress induced by the previous earthquake causes fast margin-normal shortening (*33*, *34*). As the effect of relaxation diminishes with time, the shortening slows down to approach the slower rate maintained by the locking of the megathrust (*33*). Therefore, 73 years after the 1952 earthquake, about halfway through the typical rupture recurrence interval of the southern Kamchatka megathrust, there can be sufficient energy in the system (with stress very near $\tau_{o}$). If a rupture is initiated by some triggering mechanism, then it may be able to propagate to become a large earthquake. This mechanism requires the mantle wedge viscosity to be very low (*27*). Kamchatka is the most productive in arc volcanism worldwide (*35*) due to its recent tectonic history (*36*, *37*), and seismological imaging suggests an abundance of partial melt in the mantle wedge (*38*, *39*). Therefore, it is plausible that the mantle wedge viscosity beneath Kamchatka is quite low compared to other subduction zones. Observations of postseismic deformation in the next few years combined with viscoelastic earthquake cycle modeling will provide geodetic tests for the low-viscosity hypothesis.

As discussed in Wang *et al.* (*27*), the overspending of slip deficit by one or a few earthquakes does not violate the long-term slip budget imposed by regional tectonics. Rather, it implies a more irregular earthquake recurrence pattern over time, with a mixture of recurrence intervals much shorter or longer than the average of numerous earthquake cycles. In subduction zones where multiple cycles are well documented such as Cascadia, recurrence times are hugely variable (*40*, *41*), and viscoelastic (rapid) recovery provides a mechanism to explain the relatively short intervals in the recurrence population. For assessing seismic and tsunami risks, the short interval between the 1952 and 2025 great Kamchatka earthquakes challenges the conventional thinking based on linear accumulation of slip deficit but supports the notion of time-dependent rate of energy accumulation in a viscoelastic Earth and consequently very irregular recurrence times.

## MATERIALS AND METHODS

### Rupture and deformation modeling

We construct a suite of 92 basic megathrust slip models covering the southern Kamchatka subduction zone (Fig. 3A). The rupture zone for each basic model is of an elliptical shape, and the slip distribution is of a “bell” shape, with slip peaking at the center and tapering to zero at the edge (*42*). The models vary systematically in location and shape (Fig. 3A), and the slip magnitude is adjusted to fit the tsunami waveforms (see the section below).

For each slip model, we calculate coseismic deformation of the seafloor using a three-dimensional dislocation model in a uniform elastic half-space. With kinematically prescribed fault slip, neglecting rigidity heterogeneities causes little error in modeling tsunamigenic seafloor deformation (*43*). The geometry of the megathrust is defined according to the Slab2 model (*45*) and discretized into a dense mesh of triangular fault elements each representing a point-source dislocation following Okada (*45*). The surface deformation is obtained by integrating the contributions from all the elements.

To address the effect of the sloping seafloor on tsunami generation, we applied the method of Wang *et al.* (*46*) to adjust the geometry of the shallow megathrust so that the fault depth below the flat model surface is approximately the same as the actual fault depth below seafloor. The additional uplift produced by this adjustment accurately compensates for the missing contribution of horizontal motion of the sloping seafloor (*44*).

The resultant vertical seafloor displacement is mapped into sea surface displacement by applying the Kajiura (*47*) transfer function, which accounts for the attenuation of short-wavelength components through the water column. This sea surface displacement provides the initial condition for the tsunami simulations described below.

### Tsunami modeling and quantification of basic model performance

We model tsunami propagation for each of the 92 basic slip models described above using the Tsunami-HySEA code (*48*), which solves the two-dimensional nonlinear shallow-water equations with a high-order path-conservative finite volume method.

The computational domain covers the entire Pacific Ocean through a four-level nested grid system with increasing spatial resolution toward the coastal areas where tide gauges are located. Grid spacing ranges from 4 arc min in the open ocean to 1.875 arc sec at the tide gauge sites, with intermediate transitional grids of 1 arc min and 15 arc sec resolution. This grid configuration was defined after numerous tests to ensure numerical stability, convergence of results, and computational efficiency. Bathymetric data are compiled from GEBCO (*49*) and regional sources. Regional data for Chilean sites (ARI, ANT, VAL, and TAL) are provided by the Servicio Hidrográfico y Oceanográfico de la Armada de Chile (www.shoa.cl). Data for US Pacific territory, including the Pacific Islands, Alaska, and North America sites (HIL, PAG, APR, WAK, MID, HON, UNA, ADK, ALM, SFP, SMO, SDG, and CRC), are from National Oceanic and Atmospheric Administration’s National Centers for Environmental Information (www.ngdc.noaa.gov/thredds/catalog/catalog.html). Data for Japanese sites (HAK, HAC, KUJ, MIY, ISH, AYU, and KSM) are provided by the Global Tsunami Terrain Model (*50*). To account for seawater compressibility, elastic response of solid Earth, and associated gravity effects that cause travel time delays and initial phase reversals in distant tsunamis, we apply the phase correction method of Watada *et al.* (*51*).

We linearly scale the simulated tsunami amplitude with fault slip to optimize the fit to tsunami waveforms (Fig. 3, B to E, and figs. S5 and S6). Linearity is theoretically valid in the far field, where tsunami amplitudes are small compared to the water depth and wave propagation remains within the linear regime. The performance of each basic model with optimized slip magnitude and time shifts is quantified using the NRMSE between the observed and modeled waveform segments corresponding to the leading tsunami wave. To prevent stations with larger tsunami amplitudes from dominating the misfit, the observed and modeled waveforms at each station are normalized by the root mean square amplitude of the corresponding observed waveform before computing the NRMSE. This normalization gives comparable weight to all stations. The NRMSE is then calculated as the root mean square difference between the normalized observed and modeled waveforms. The NRMSE is therefore dimensionless, with lower values indicating better agreement between the observed and modeled tsunami waveforms.

### Weighted averages of basic models

Each of the four slip models in Fig. 4 (A to C and E) is a weighted average of the 92 basic models for the relevant earthquake. The tsunami waveforms predicted by each basic model at each station were treated as Green’s functions, and the corresponding weights were solved using a coupled, three-step iterative nonnegative least-squares (NNLS) inversion implemented with MATLAB’s lsqnonneg algorithm (*52*).

The goal of the first step is to narrow down uncertainties in the travel time of the tsunami records, particularly for the 1952 event. This step is essential because tsunami waveform inversion is sensitive to phase alignment: Even small time shifts can strongly affect the correlation between observed and simulated waveforms, leading to erroneous slip estimates. Each of the 24 stations (shown in bold in Fig. 1, E to J) is analyzed independently by performing an NNLS fit for a series of trial time shifts using the 92 model waveforms. The trial shifts are tested within a predefined window (e.g., −10 to 10 min), which specifies the allowed range for aligning the observed and modeled records. The choice of window limits depends on the assumptions regarding ∆t= 0 or ∆t≤0 as described in the “More nuanced estimates of the two rupture zones” section. For each trial shift, an NNLS fit is solved to produce a modeled waveform for that alignment, and the shift yielding the highest correlation with the observed waveform is selected. This process provides an initial, consistent “best–time shift” alignment for all stations but does not eliminate all timing uncertainty; these best time shifts are further refined in the next step.

In the second step, the 24 stations are combined, using their current best time shifts, in a single global NNLS inversion to obtain an initial slip model as the optimal linear combination of the 92 basic models. However, this initial model still inherits residual timing errors from the first step, as the best time shifts were estimated independently at each station rather than jointly with one global solution.

In the third step, we iteratively refine both the slip model and the time shifts in a coupled manner by comparing each observed waveform with the synthetic waveform predicted by the global model and adjusting the shifts to maximize correlation. The global inversion and time-shift refinement are repeated a few times (typically two or three) until the overall fit stabilizes.

For the models in Fig. 4 (A to C), although 92 basic models are used in each average, only a small number of them actually acquire nonnegligible weights and contribute to the final solution (fig. S7). These models are automatically selected by the inversion for their specific attributes to account for various features of the tsunami data, and the selection does not reflect their performance as standalone models. For example, TB3 as a standalone model fits the 1952 tsunami waveforms very poorly (fig. S6), but it contributes 39% to the 1952 “shorter travel times” weighted average (Fig. 4C). For the “minimum overlap” model in Fig. 4E, not all 92 basic models are used as explained in the “More nuanced estimates of the two rupture zones” section. The NRMSE values reported in Fig. 4 are computed in the same manner as those for the basic models.

## Supplementary Materials

### The PDF file includes:

Figs. S1 to S7 Table S1 Legend for data S1 References
