# Supplementary material for: Pacific tsunami records suggest similar rupture locations of two great Kamchatka earthquakes only 73 years apart
Source: Sci Adv. 2026 Sep 23;12(39):eaee5294. doi: 10.1126/sciadv.aee5294 (PMC13600182; doi:10.1126/sciadv.aee5294)
Supplement: Supplementary file 1 [file sciadv.aee5294_sm.pdf]

Science Advances
AAAS

Supplementary Materials for

Pacific tsunami records suggest similar rupture locations of two great Kamchatka earthquakes only 73 years apart

Matías Carvajal *et al.*

Corresponding author: Matías Carvajal, matias.carvajal@pucv.cl

Sci. Adv. **12**, eaee5294 (2026)
DOI: 10.1126/sciadv.aee5294

The PDF file includes:

Figs. S1 to S7
Table S1
Legend for data S1
References

Other Supplementary Material for this manuscript includes the following:

Data S1

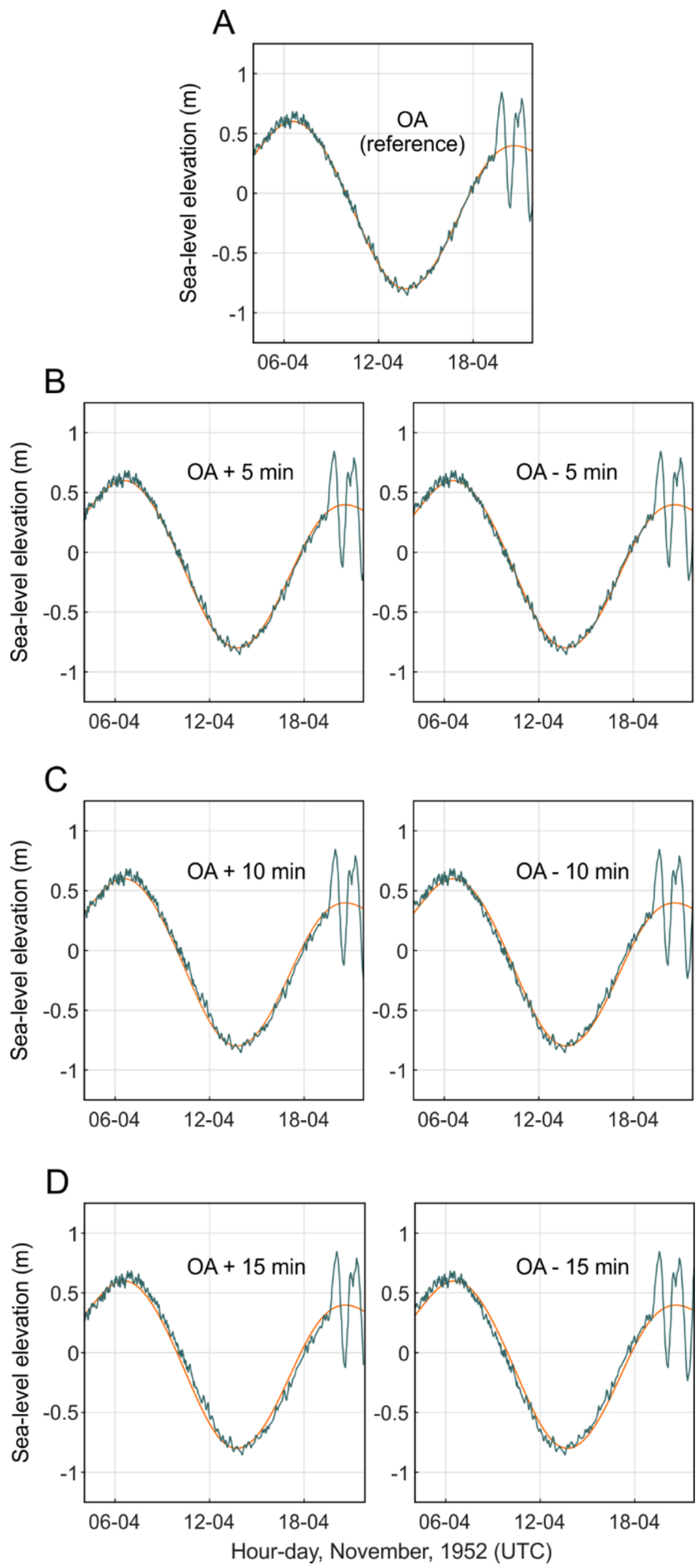

Fig. S1. Example of time-shift tests illustrating the presence of travel-time uncertainties in the 1952 records optimally aligned with hindcast tides. (A) Optimal alignment (OA) at station HAC as in Fig. 2B. (B) OA shifted by ±5 min, still showing acceptable alignment. (C) OA shifted by ±10 min, and misalignment is visually identifiable. (D) OA shifted by ±15 min, and the alignment is clearly unacceptable.

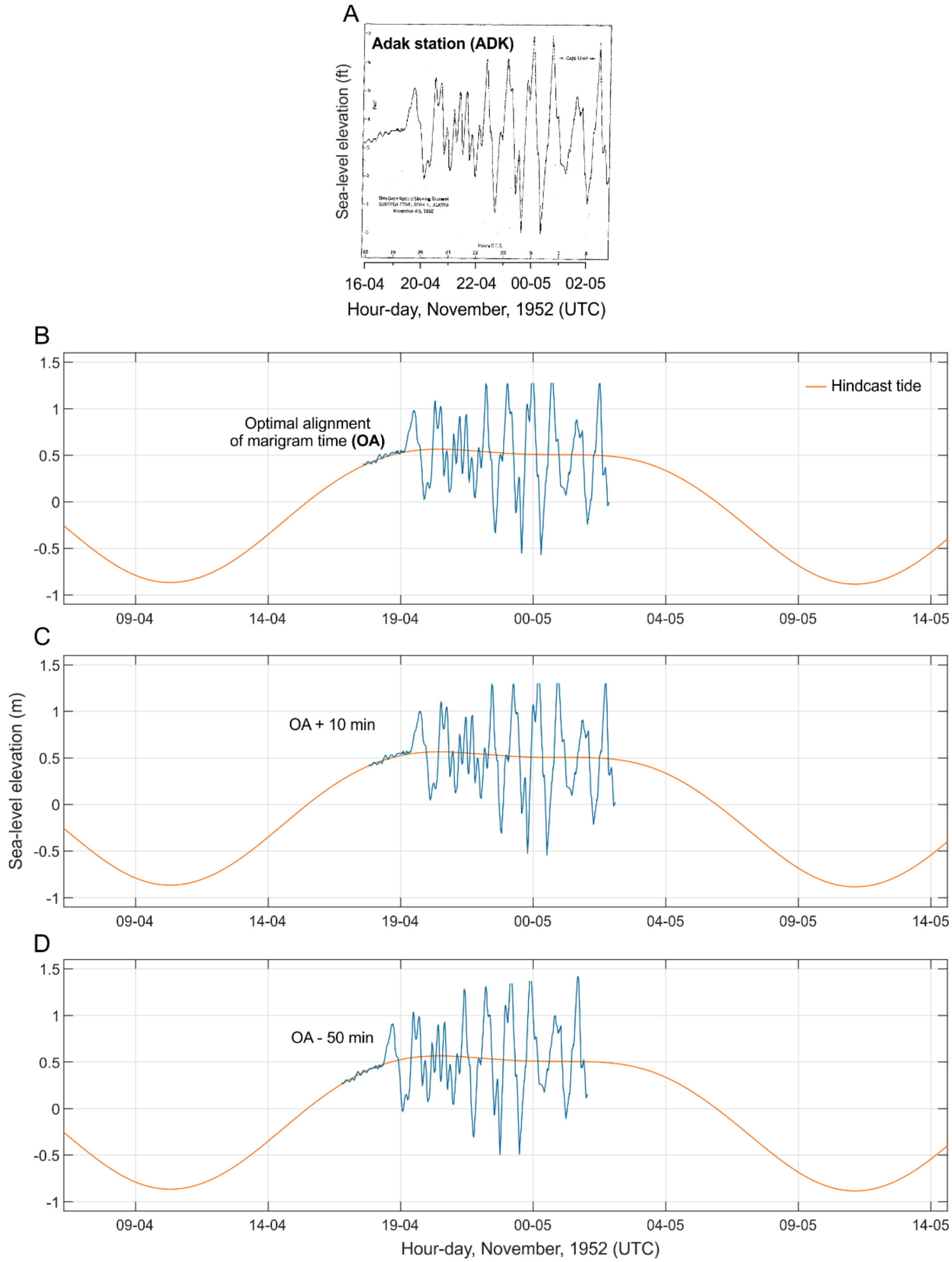

Fig. S2. Example illustrating how very large time uncertainty arises in some 1952 records if the marigram is too short. (A) Marigram at station ADK provided in digitized form by Zerbe (46). (B) Digitized record with optimal alignment (OA) with hindcast tides. (C and D) OA shifted by +10 min and -50 min, respectively, with some vertical shift and rotation of the entire marigram segment, which is still visually acceptable. Shifts larger than +10 min or −50 min become visually unacceptable.

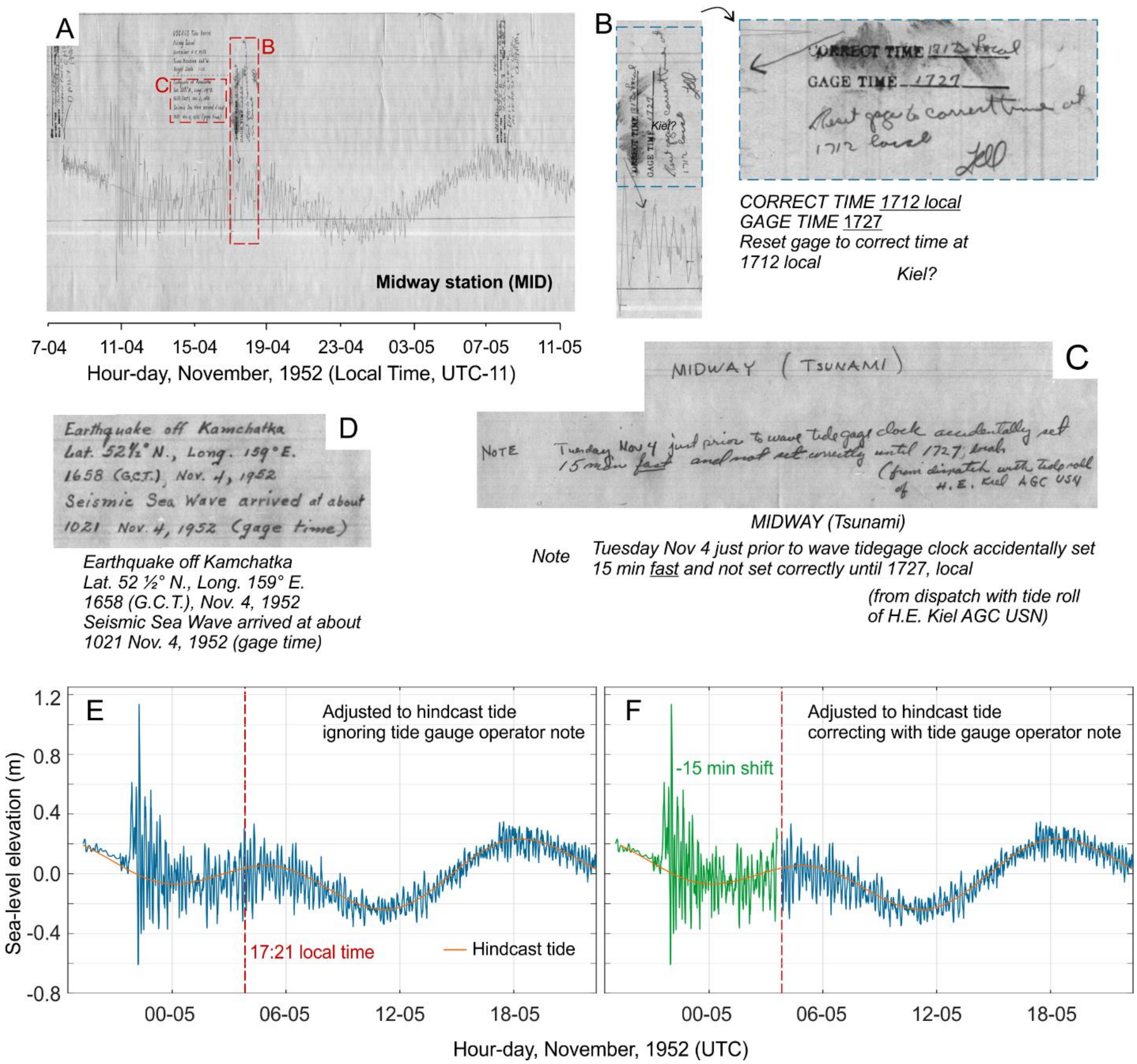

Fig. S3. Example showing how handwritten notes in original paper marigrams can help correct instrument clock errors in the 1952 records. (A) Original paper marigram at station MID with handwritten marks indicating a 15-min desynchronization of the tide-gauge clock before 17:27 local time and its correction by a clock reset. (B) Enlarged view of the mark for the time of the clock reset. (C) Enlarged view of the note of correction. (D) Operator’s handwritten note on the arrival of the tsunami. Given the 1952 earthquake origin time (16:58 UTC, Nov 4) and the 15-minute clock correction, if we assume that the arrival of the “seismic sea wave” corresponds to the leading wave crest, the tsunami arrival at 10:21 gives a travel time of 4 h 08 min to MID. This is 6 min shorter than that of 2025. (E) Raw digitized record before the clock reset showing misalignment with the hindcast tide. (F) Same record corrected by shifting the first third of the record (green) by −15 min based on the operator’s note, showing improved alignment.

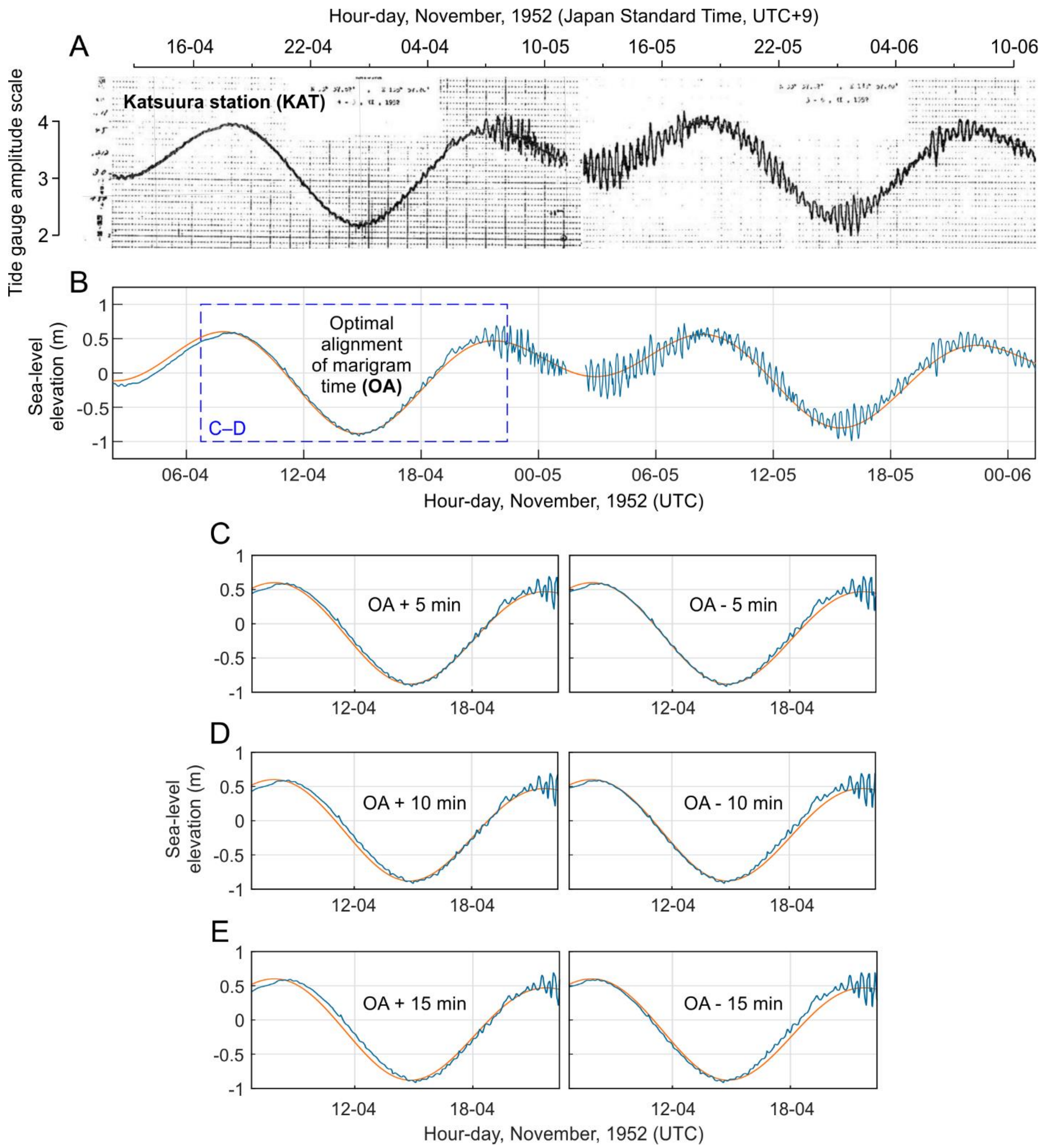

Fig. S4. Example illustrating the effect of poor quality of original paper marigrams. (A) Original paper marigram at station KAT. (B) Digitized record with optimal alignment (OA) with hindcast tides. The tidal period before the tsunami in the record is shorter than the hindcast tide for unknown reason, which makes the OA more ambiguous. (C – E) OA shifted by different amounts in comparison with hindcast tides. By focusing on the tidal cycle containing the tsunami arrival, the time error is still deemed to be small.

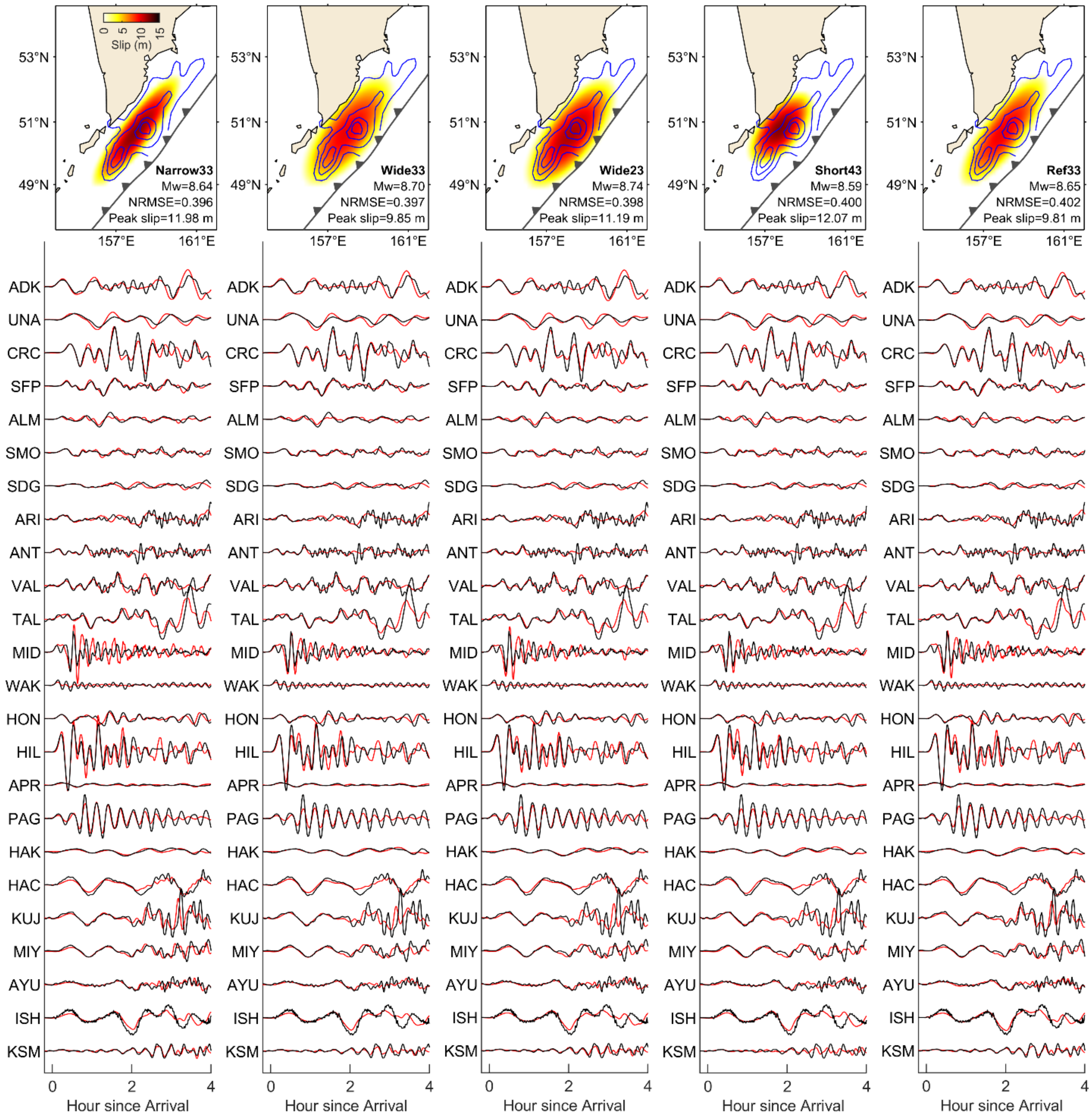

Fig. S5. Comparison between observed 2025 tsunami waveforms and predictions from 92 basic slip models. Black and red lines are observed and model-predicted waveforms, respectively. For each basic model, the upper panel shows the moment magnitude (Mw), Normalized root-mean-square error (NRMSE) of fit, and peak slip. Blue lines in maps are 2, 6, 10, and 12 m slip contours of the best model of Liu et al. (13). The vertical spacing between stations is 1.5 m and provides a reference scale for tsunami amplitude.

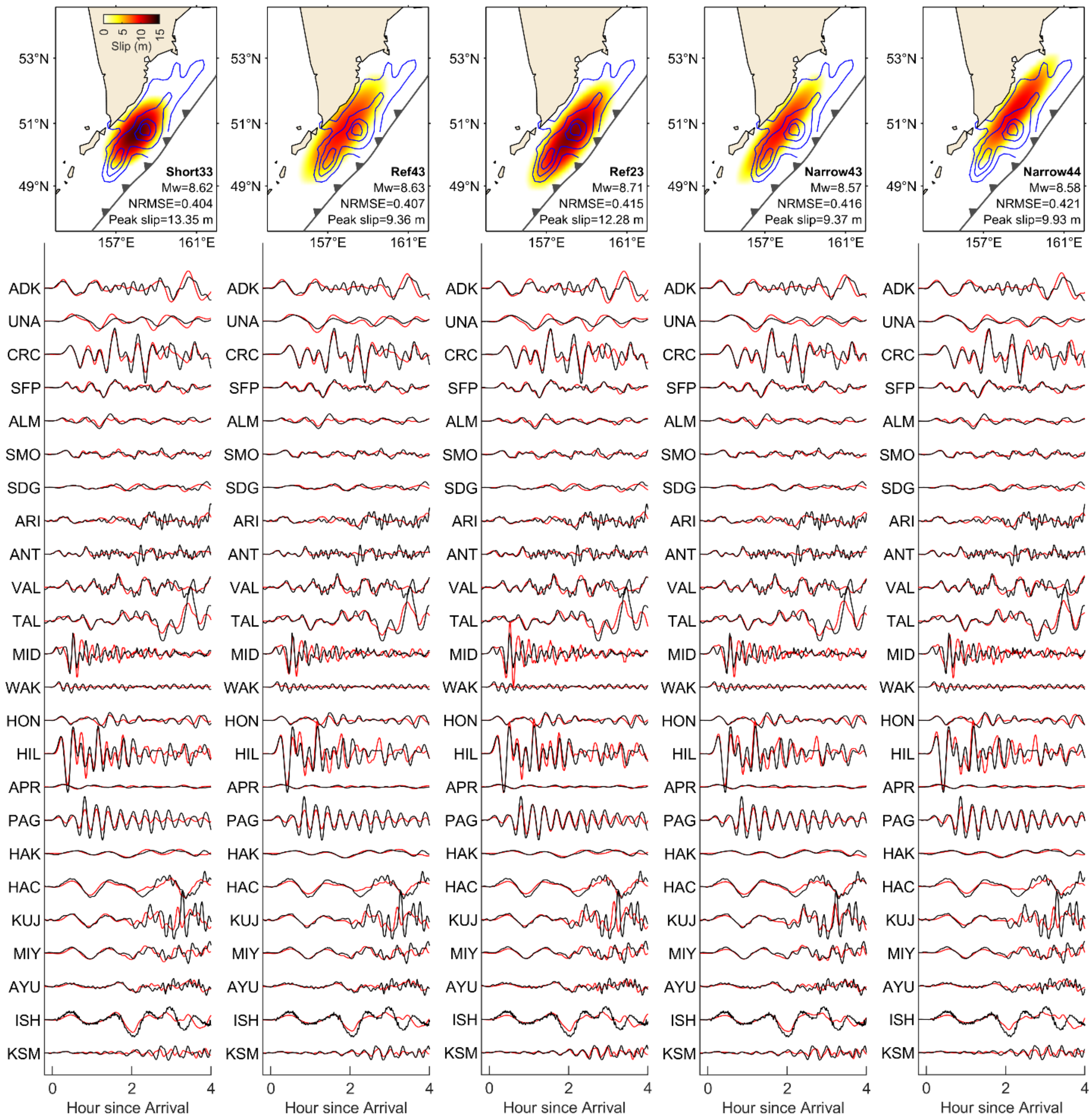
Slip (m)
0 5 10 15
53°N
51°N
49°N
157°E
161°E
Short33
Mw=8.62
NRMSE=0.404
Peak slip=13.35 m
Ref43
Mw=8.63
NRMSE=0.407
Peak slip=9.36 m
Ref23
Mw=8.71
NRMSE=0.415
Peak slip=12.28 m
Narrow43
Mw=8.57
NRMSE=0.416
Peak slip=9.37 m
Narrow44
Mw=8.58
NRMSE=0.421
Peak slip=9.93 m
ADK
UNA
CRC
SFP
ALM
SMO
SDG
ARI
ANT
VAL
TAL
MID
WAK
HON
HIL
APR
PAG
HAK
HAC
KUJ
MIY
AYU
ISH
KSM
0
2
4
Hour since Arrival

Fig. S5 (cont.).

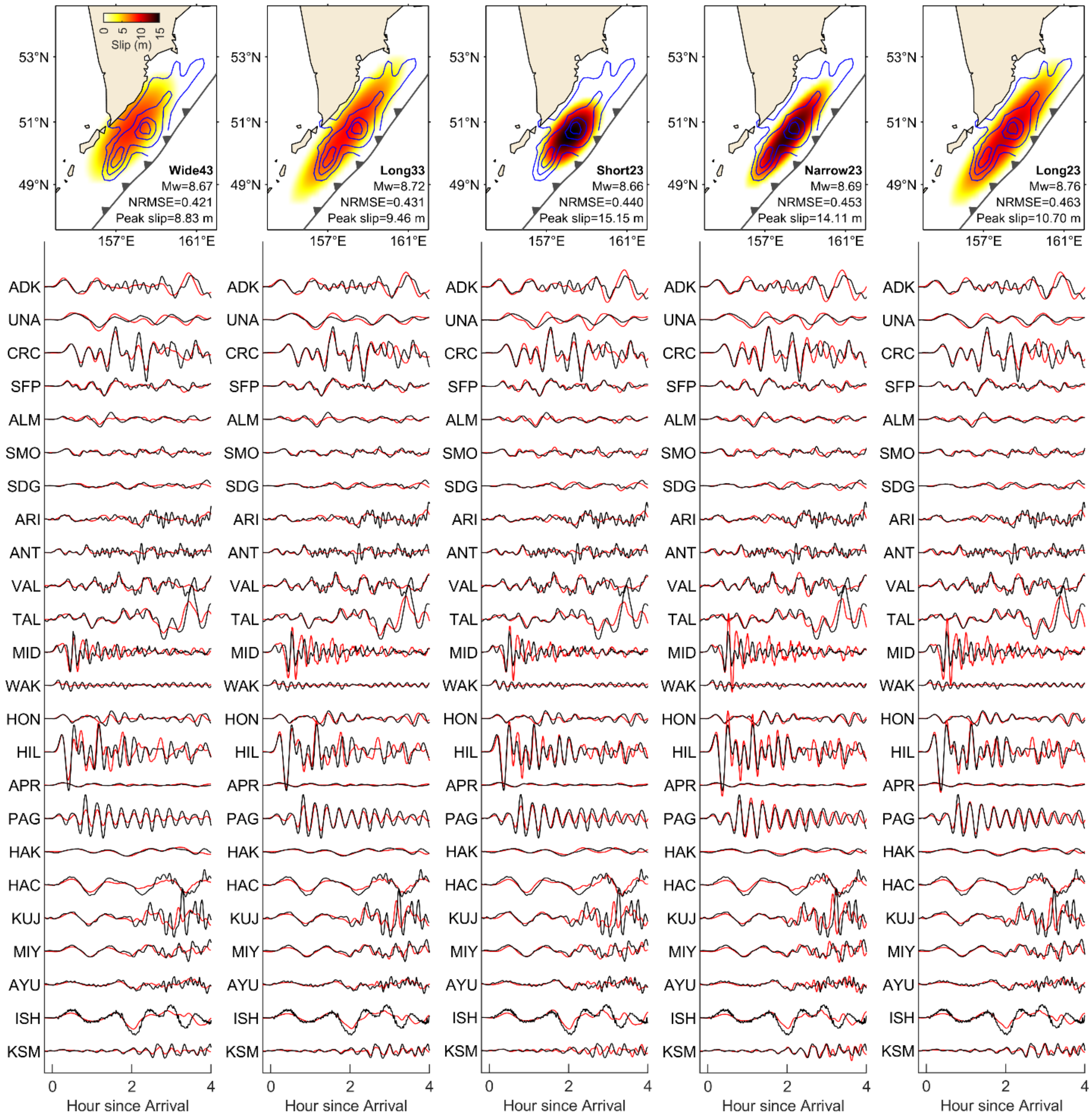

Slip (m)
0 5 10 15
53°N
51°N
49°N
157°E
161°E
Wide43
Mw=8.67
NRMSE=0.421
Peak slip=8.83 m
Long33
Mw=8.72
NRMSE=0.431
Peak slip=9.46 m
Short23
Mw=8.66
NRMSE=0.440
Peak slip=15.15 m
Narrow23
Mw=8.69
NRMSE=0.453
Peak slip=14.11 m
Long23
Mw=8.76
NRMSE=0.463
Peak slip=10.70 m
ADK
UNA
CRC
SFP
ALM
SMO
SDG
ARI
ANT
VAL
TAL
MID
WAK
HON
HIL
APR
PAG
HAK
HAC
KUJ
MIY
AYU
ISH
KSM
0
2
4
Hour since Arrival

Fig. S5 (cont.).

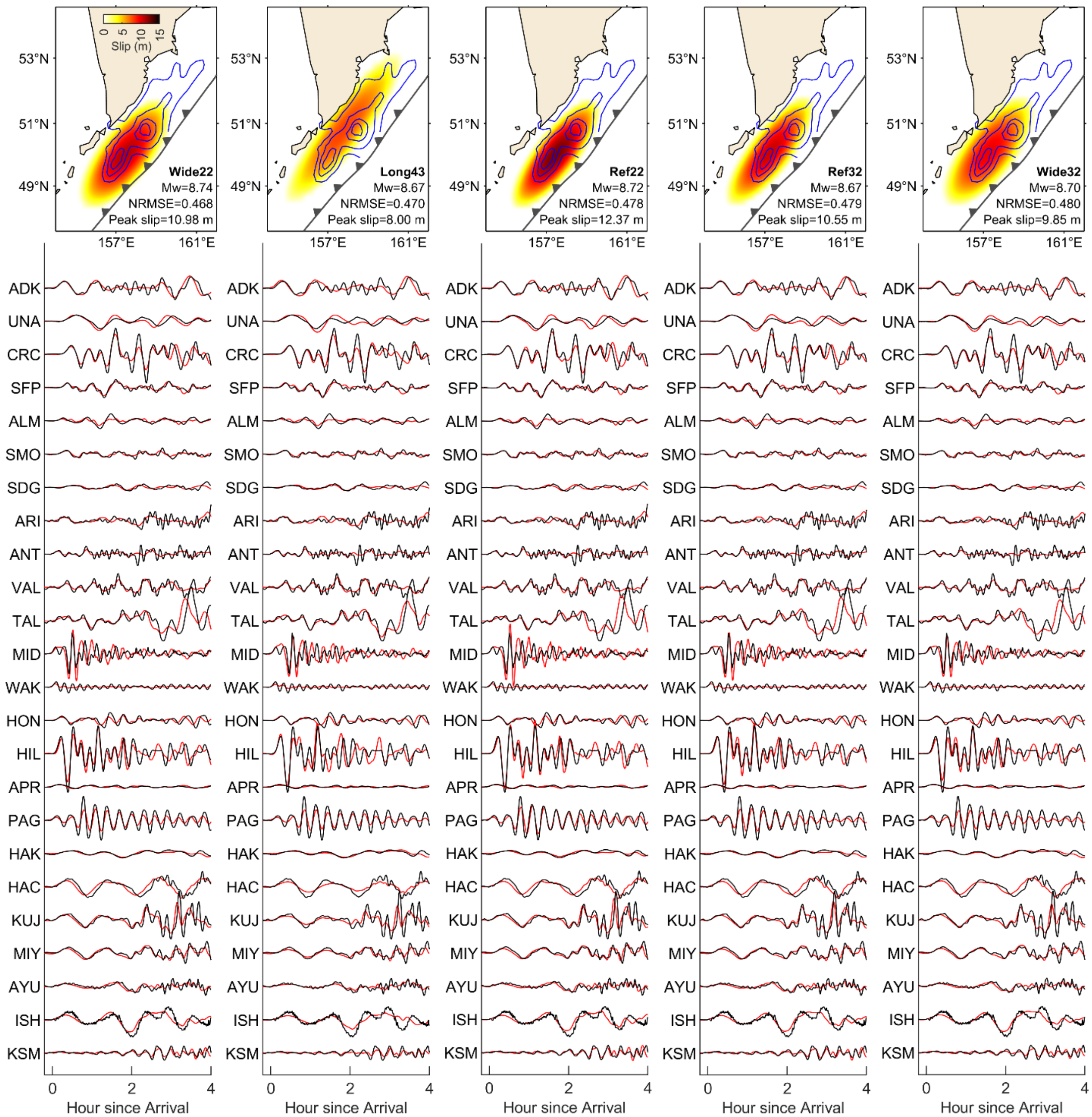

Slip (m)
0
5
10
15
53°N
51°N
49°N
157°E
161°E
Wide22
Mw=8.74
NRMSE=0.468
Peak slip=10.98 m
Long43
Mw=8.67
NRMSE=0.470
Peak slip=8.00 m
Ref22
Mw=8.72
NRMSE=0.478
Peak slip=12.37 m
Ref32
Mw=8.67
NRMSE=0.479
Peak slip=10.55 m
Wide32
Mw=8.70
NRMSE=0.480
Peak slip=9.85 m
ADK
UNA
CRC
SFP
ALM
SMO
SDG
ARI
ANT
VAL
TAL
MID
WAK
HON
HIL
APR
PAG
HAK
HAC
KUJ
MIY
AYU
ISH
KSM
0
2
4
Hour since Arrival

Fig. S5 (cont.).

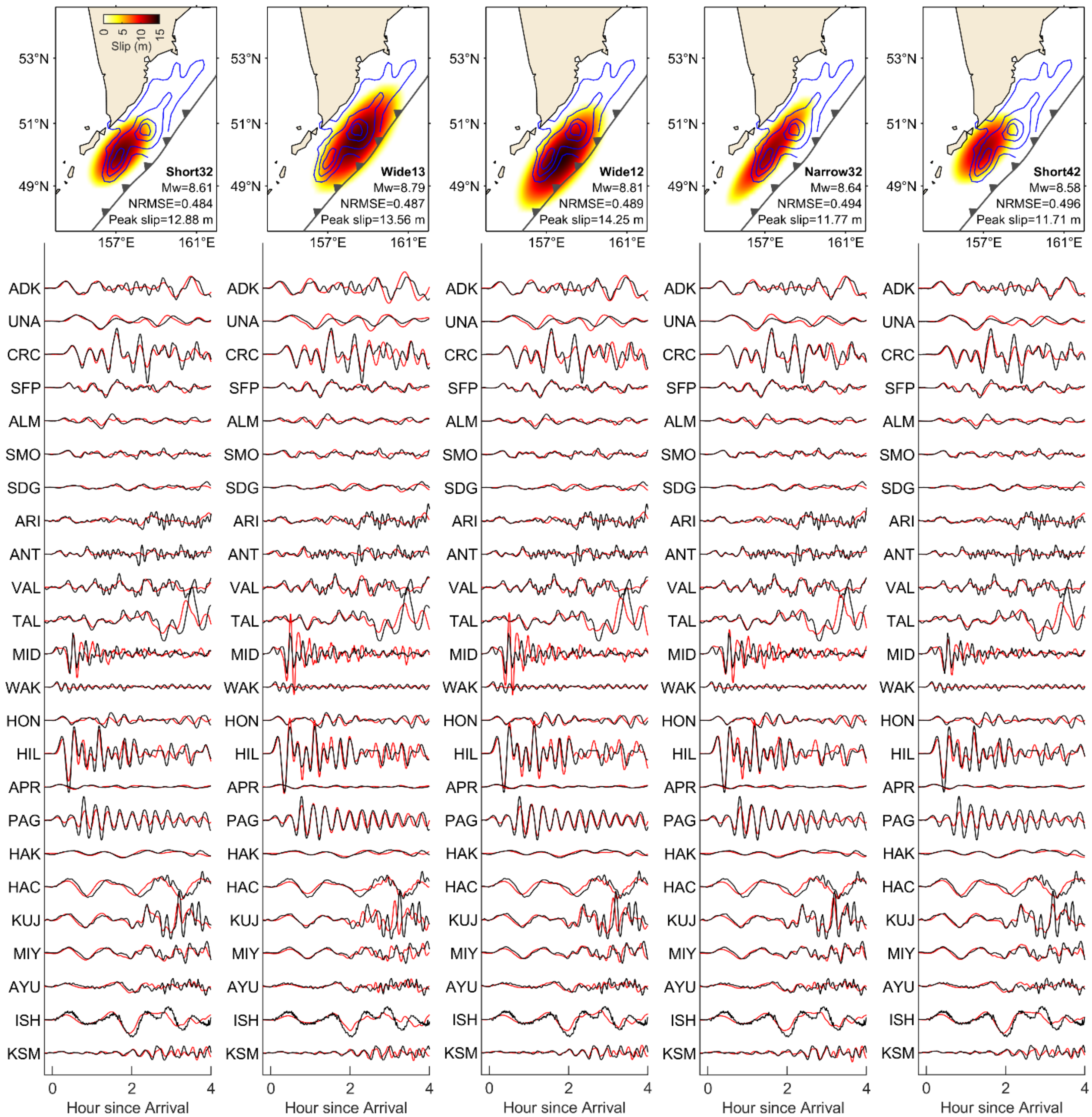

Slip (m)
0 5 10 15
53°N
51°N
49°N
157°E
161°E
Short32
Mw=8.61
NRMSE=0.484
Peak slip=12.88 m
Wide13
Mw=8.79
NRMSE=0.487
Peak slip=13.56 m
Wide12
Mw=8.81
NRMSE=0.489
Peak slip=14.25 m
Narrow32
Mw=8.64
NRMSE=0.494
Peak slip=11.77 m
Short42
Mw=8.58
NRMSE=0.496
Peak slip=11.71 m
ADK
UNA
CRC
SFP
ALM
SMO
SDG
ARI
ANT
VAL
TAL
MID
WAK
HON
HIL
APR
PAG
HAK
HAC
KUJ
MIY
AYU
ISH
KSM
0
2
4
Hour since Arrival

Fig. S5 (cont.).

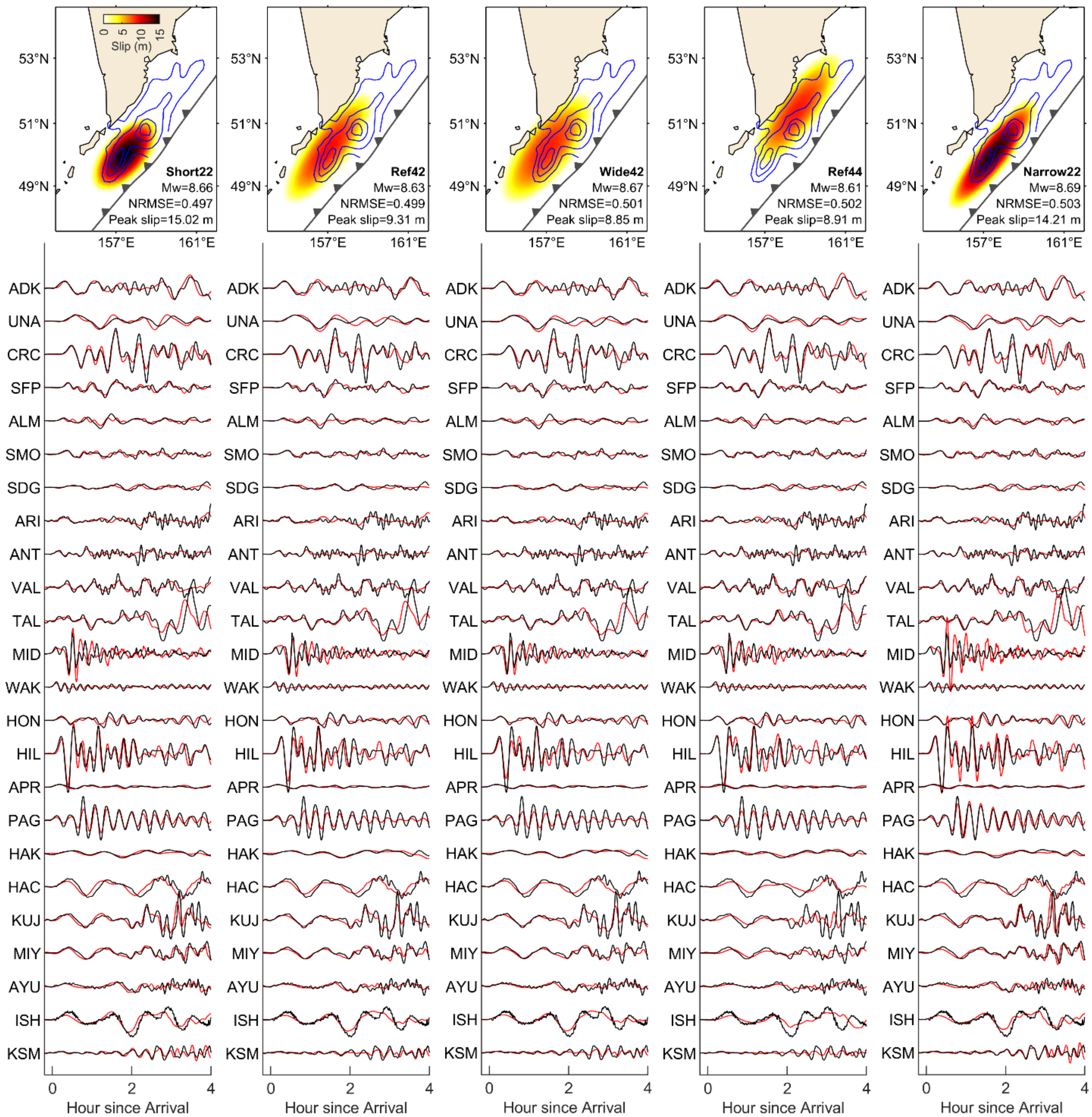

Slip (m)
0 5 10 15
53°N
51°N
49°N
157°E
161°E
Short22
Mw=8.66
NRMSE=0.497
Peak slip=15.02 m
Ref42
Mw=8.63
NRMSE=0.499
Peak slip=9.31 m
Wide42
Mw=8.67
NRMSE=0.501
Peak slip=8.85 m
Ref44
Mw=8.61
NRMSE=0.502
Peak slip=8.91 m
Narrow22
Mw=8.69
NRMSE=0.503
Peak slip=14.21 m
ADK
UNA
CRC
SFP
ALM
SMO
SDG
ARI
ANT
VAL
TAL
MID
WAK
HON
HIL
APR
PAG
HAK
HAC
KUJ
MIY
AYU
ISH
KSM
0 2 4
Hour since Arrival

Fig. S5 (cont.).

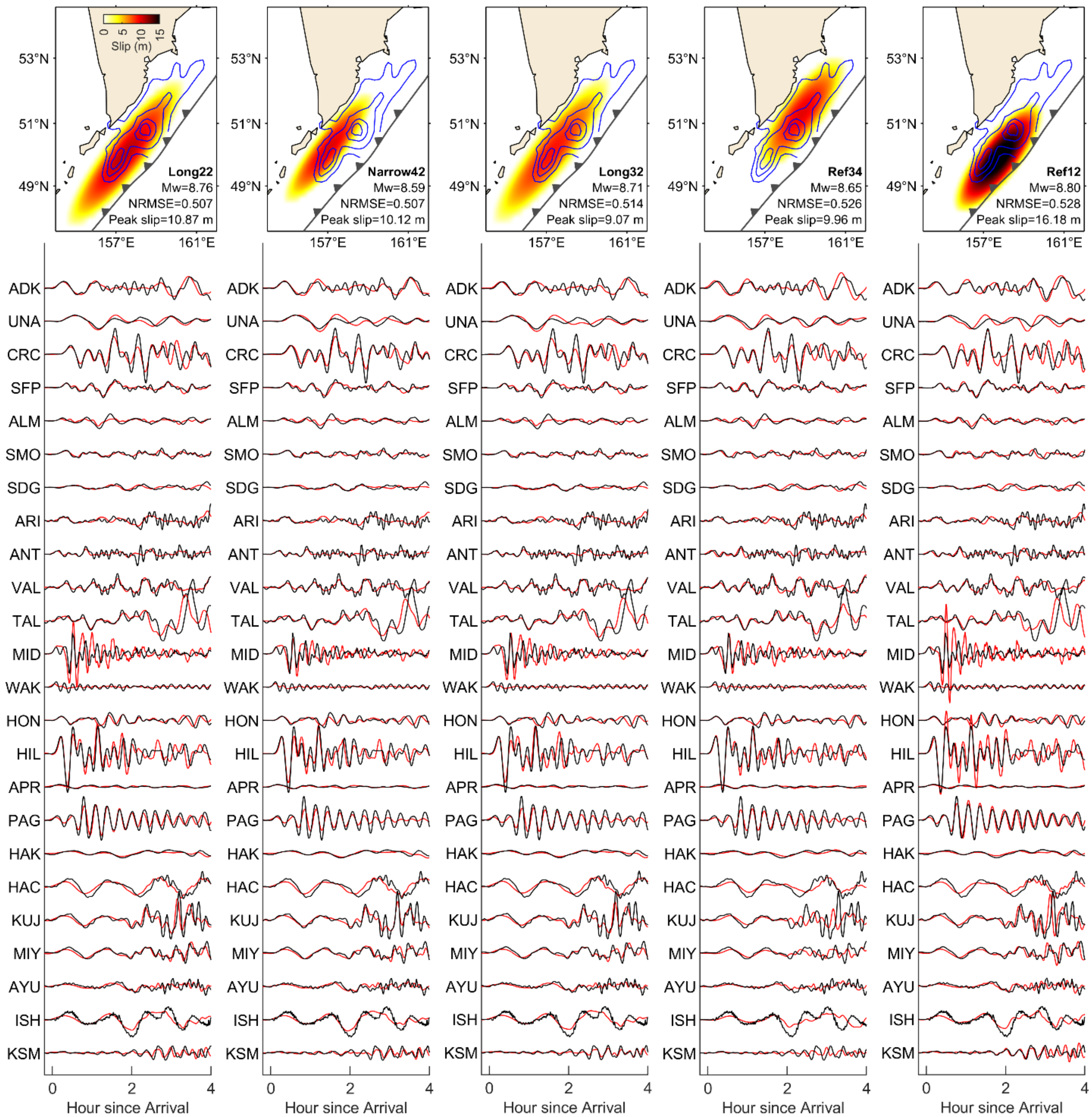
Slip (m)
0 5 10 15
53°N
51°N
49°N
157°E
161°E
Long22
Mw=8.76
NRMSE=0.507
Peak slip=10.87 m
Narrow42
Mw=8.59
NRMSE=0.507
Peak slip=10.12 m
Long32
Mw=8.71
NRMSE=0.514
Peak slip=9.07 m
Ref34
Mw=8.65
NRMSE=0.526
Peak slip=9.96 m
Ref12
Mw=8.80
NRMSE=0.528
Peak slip=16.18 m
ADK
UNA
CRC
SFP
ALM
SMO
SDG
ARI
ANT
VAL
TAL
MID
WAK
HON
HIL
APR
PAG
HAK
HAC
KUJ
MIY
AYU
ISH
KSM
0
2
4
Hour since Arrival

Fig. S5 (cont.).

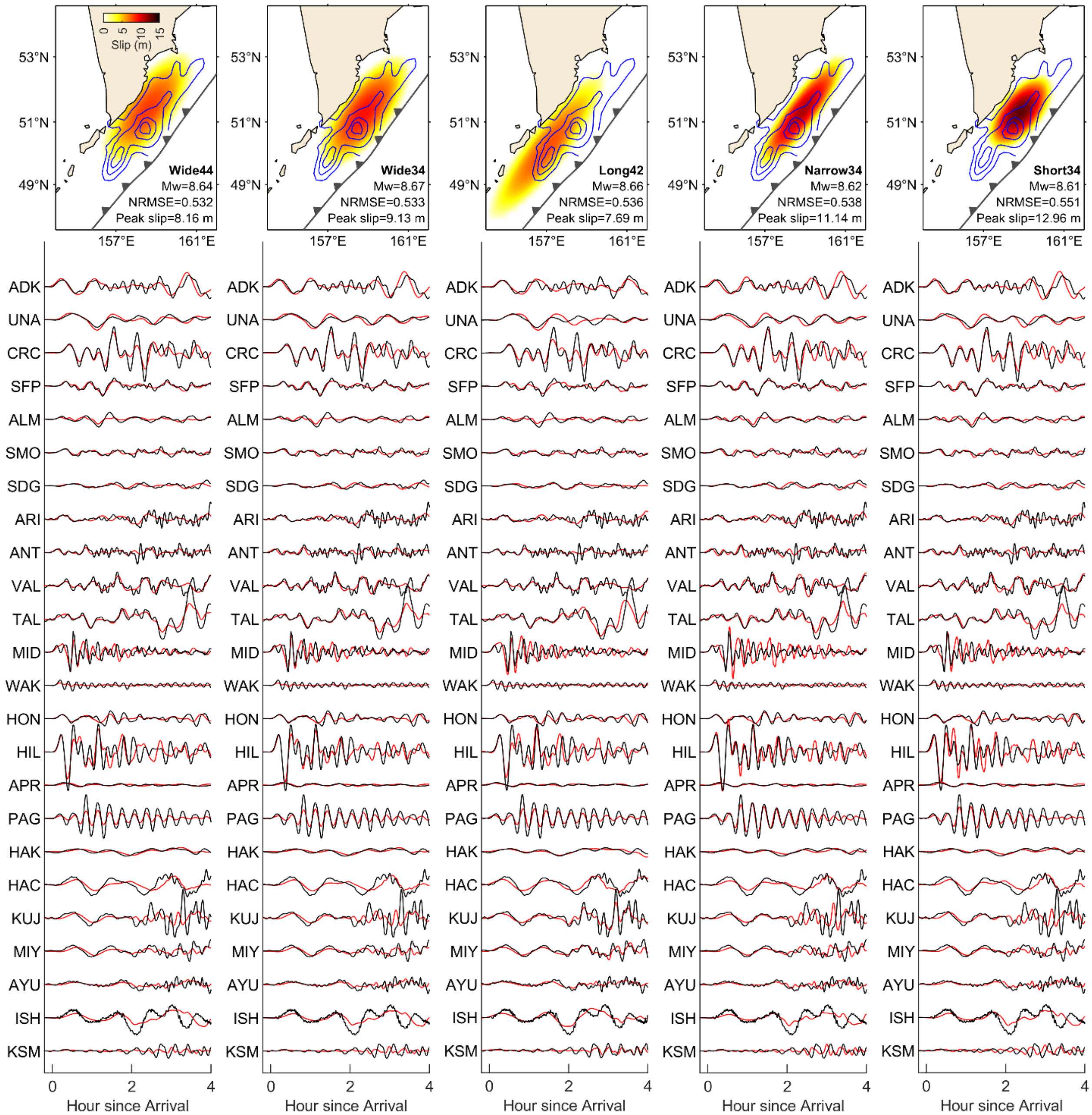

Slip (m)
0 5 10 15
53°N
51°N
49°N
157°E
161°E
Wide44
Mw=8.64
NRMSE=0.532
Peak slip=8.16 m
Wide34
Mw=8.67
NRMSE=0.533
Peak slip=9.13 m
Long42
Mw=8.66
NRMSE=0.536
Peak slip=7.69 m
Narrow34
Mw=8.62
NRMSE=0.538
Peak slip=11.14 m
Short34
Mw=8.61
NRMSE=0.551
Peak slip=12.96 m
ADK
UNA
CRC
SFP
ALM
SMO
SDG
ARI
ANT
VAL
TAL
MID
WAK
HON
HIL
APR
PAG
HAK
HAC
KUJ
MIY
AYU
ISH
KSM
0 2 4
Hour since Arrival

Fig. S5 (cont.).

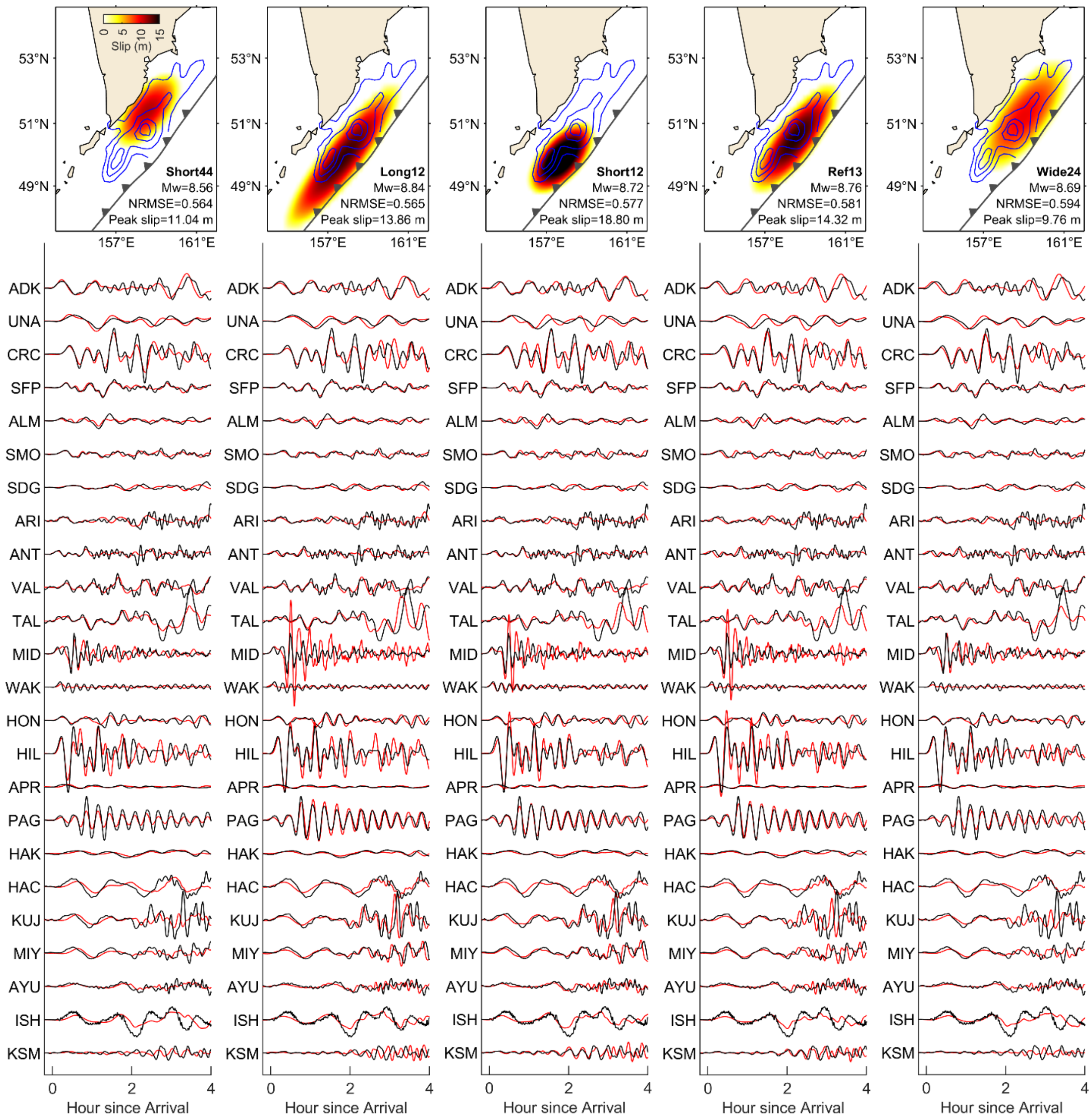
Slip (m)
0 5 10 15
53°N
51°N
49°N
157°E
161°E
Short44
Mw=8.56
NRMSE=0.564
Peak slip=11.04 m
Long12
Mw=8.84
NRMSE=0.565
Peak slip=13.86 m
Short12
Mw=8.72
NRMSE=0.577
Peak slip=18.80 m
Ref13
Mw=8.76
NRMSE=0.581
Peak slip=14.32 m
Wide24
Mw=8.69
NRMSE=0.594
Peak slip=9.76 m
ADK
UNA
CRC
SFP
ALM
SMO
SDG
ARI
ANT
VAL
TAL
MID
WAK
HON
HIL
APR
PAG
HAK
HAC
KUJ
MIY
AYU
ISH
KSM
0 2 4
Hour since Arrival

Fig. S5 (cont.).

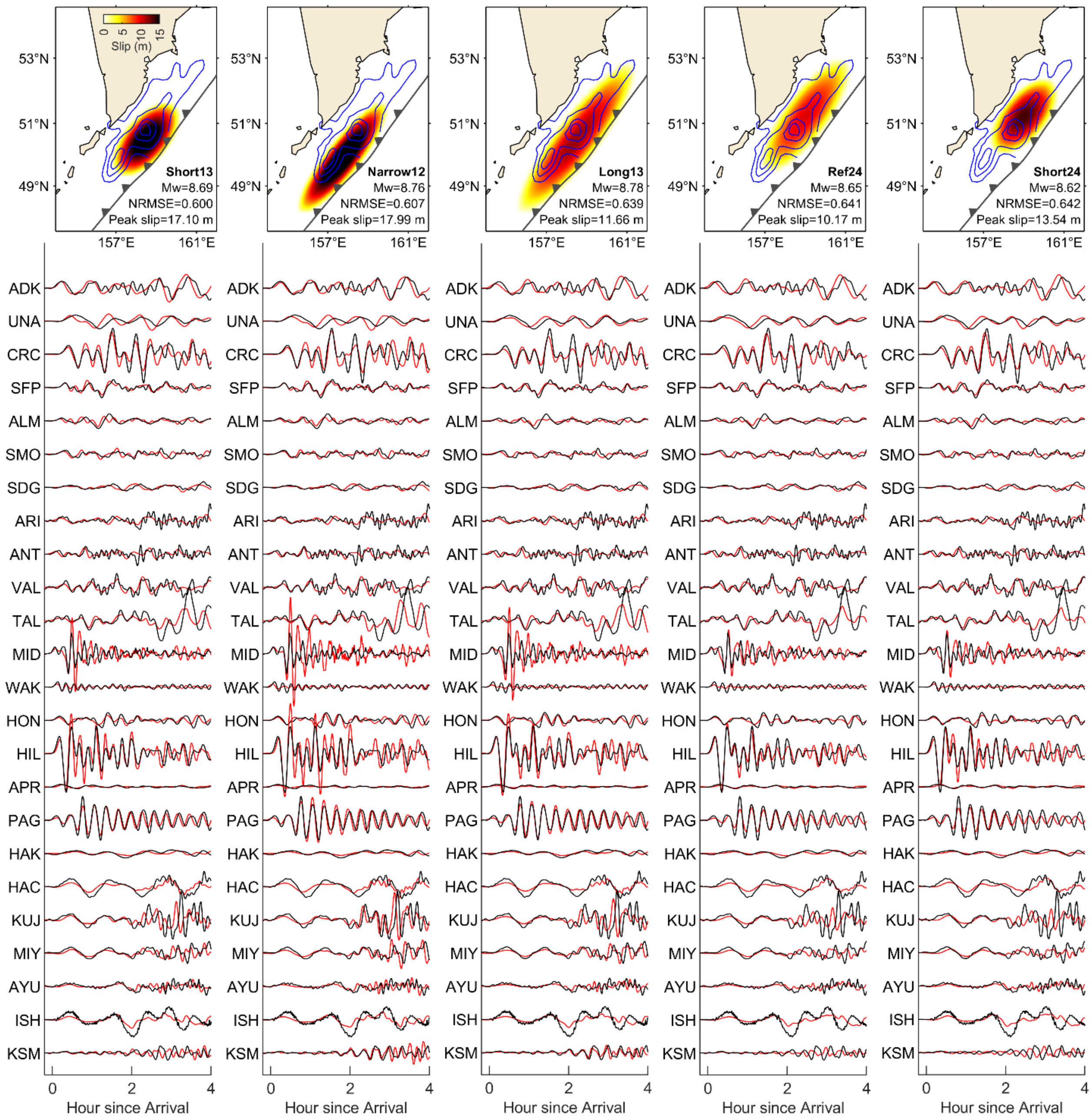

Slip (m)
0 5 10 15
53°N
51°N
49°N
157°E
161°E
Short13
Mw=8.69
NRMSE=0.600
Peak slip=17.10 m
Narrow12
Mw=8.76
NRMSE=0.607
Peak slip=17.99 m
Long13
Mw=8.78
NRMSE=0.639
Peak slip=11.66 m
Ref24
Mw=8.65
NRMSE=0.641
Peak slip=10.17 m
Short24
Mw=8.62
NRMSE=0.642
Peak slip=13.54 m
ADK
UNA
CRC
SFP
ALM
SMO
SDG
ARI
ANT
VAL
TAL
MID
WAK
HON
HIL
APR
PAG
HAK
HAC
KUJ
MIY
AYU
ISH
KSM
0 2 4
Hour since Arrival

Fig. S5 (cont.).

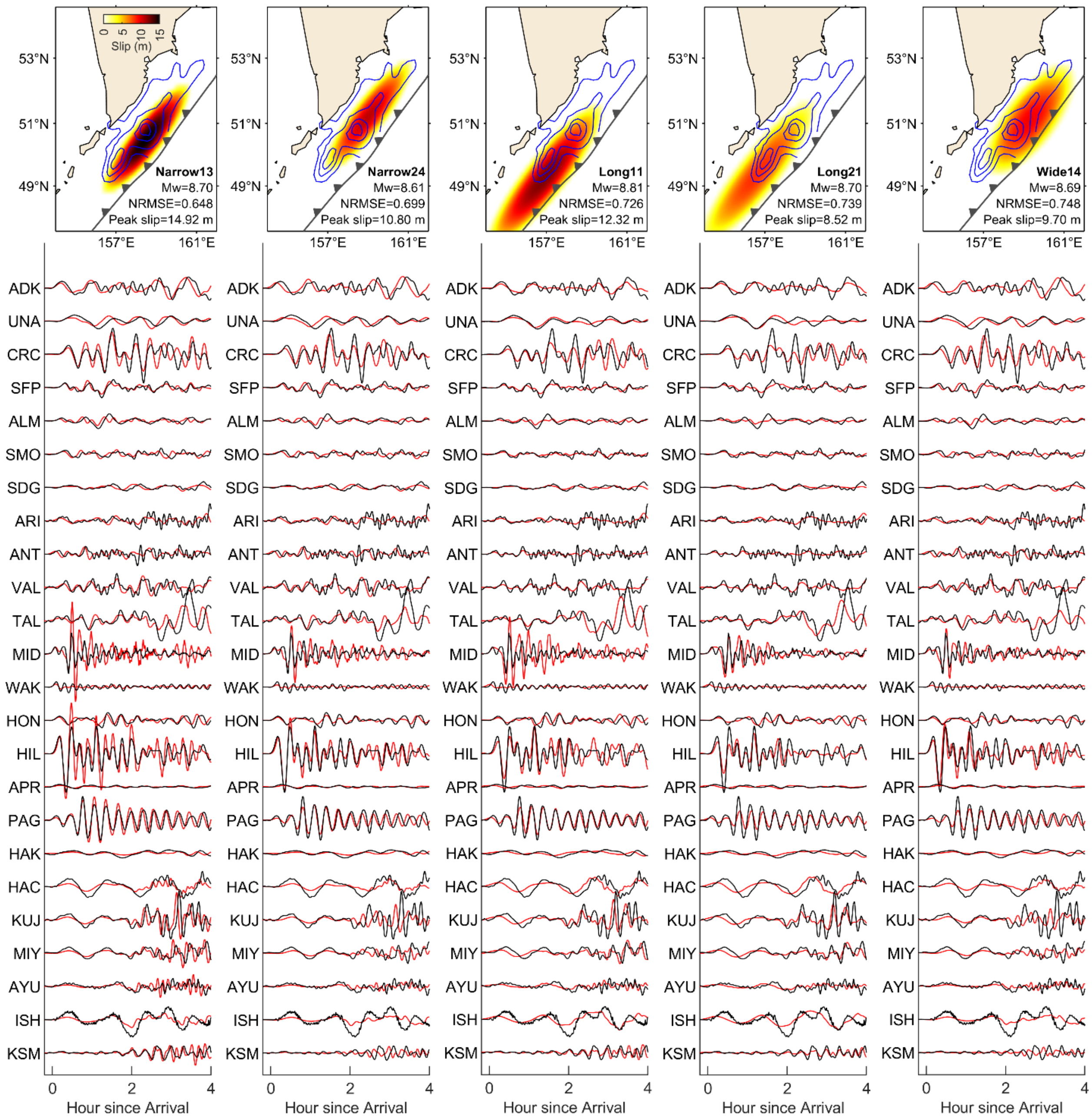

Slip (m)
0
5
10
15
53°N
51°N
49°N
157°E
161°E
Narrow13
Mw=8.70
NRMSE=0.648
Peak slip=14.92 m
Narrow24
Mw=8.61
NRMSE=0.699
Peak slip=10.80 m
Long11
Mw=8.81
NRMSE=0.726
Peak slip=12.32 m
Long21
Mw=8.70
NRMSE=0.739
Peak slip=8.52 m
Wide14
Mw=8.69
NRMSE=0.748
Peak slip=9.70 m
ADK
UNA
CRC
SFP
ALM
SMO
SDG
ARI
ANT
VAL
TAL
MID
WAK
HON
HIL
APR
PAG
HAK
HAC
KUJ
MIY
AYU
ISH
KSM
0
2
4
Hour since Arrival

Fig. S5 (cont.).

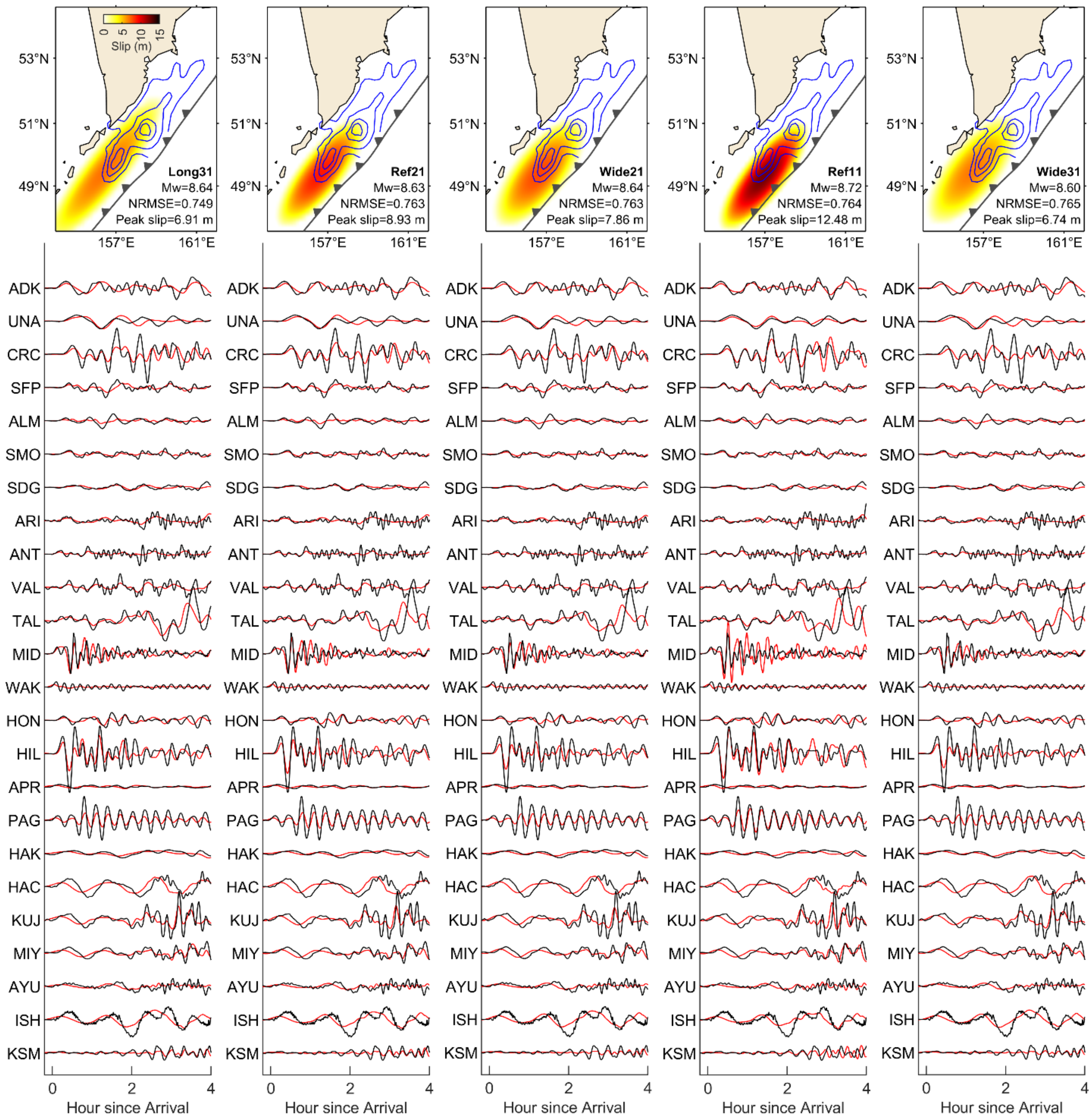

Slip (m)
0 5 10 15
53°N
51°N
49°N
157°E 161°E
Long31
Mw=8.64
NRMSE=0.749
Peak slip=6.91 m
Ref21
Mw=8.63
NRMSE=0.763
Peak slip=8.93 m
Wide21
Mw=8.64
NRMSE=0.763
Peak slip=7.86 m
Ref11
Mw=8.72
NRMSE=0.764
Peak slip=12.48 m
Wide31
Mw=8.60
NRMSE=0.765
Peak slip=6.74 m
ADK
UNA
CRC
SFP
ALM
SMO
SDG
ARI
ANT
VAL
TAL
MID
WAK
HON
HIL
APR
PAG
HAK
HAC
KUJ
MIY
AYU
ISH
KSM
0 2 4
Hour since Arrival

Fig. S5 (cont.).

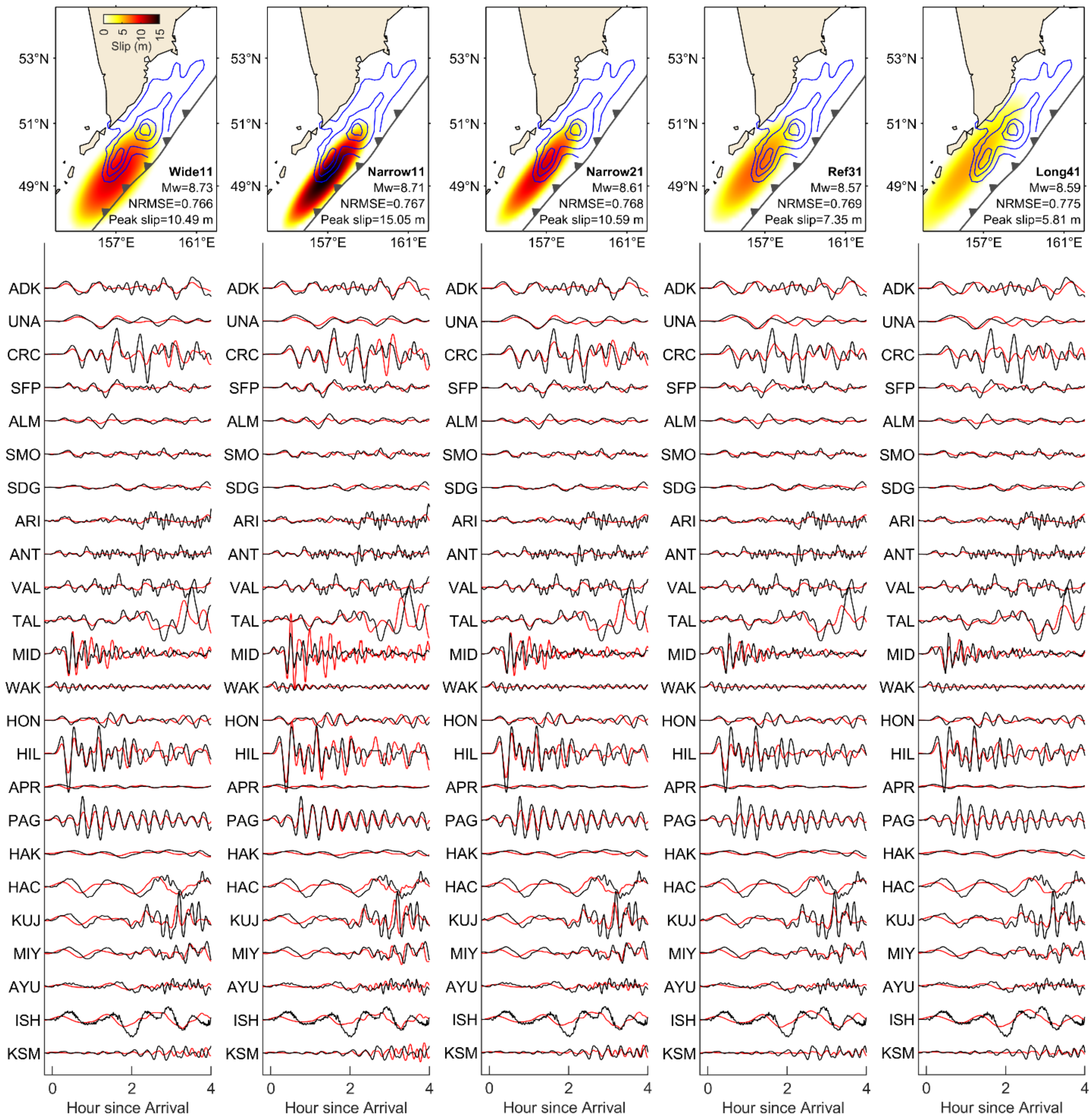

Slip (m)
0 5 10 15
53°N
51°N
49°N
157°E
161°E
Wide11
Mw=8.73
NRMSE=0.766
Peak slip=10.49 m
Narrow11
Mw=8.71
NRMSE=0.767
Peak slip=15.05 m
Narrow21
Mw=8.61
NRMSE=0.768
Peak slip=10.59 m
Ref31
Mw=8.57
NRMSE=0.769
Peak slip=7.35 m
Long41
Mw=8.59
NRMSE=0.775
Peak slip=5.81 m
ADK
UNA
CRC
SFP
ALM
SMO
SDG
ARI
ANT
VAL
TAL
MID
WAK
HON
HIL
APR
PAG
HAK
HAC
KUJ
MIY
AYU
ISH
KSM
0 2 4
Hour since Arrival

Fig. S5 (cont.).

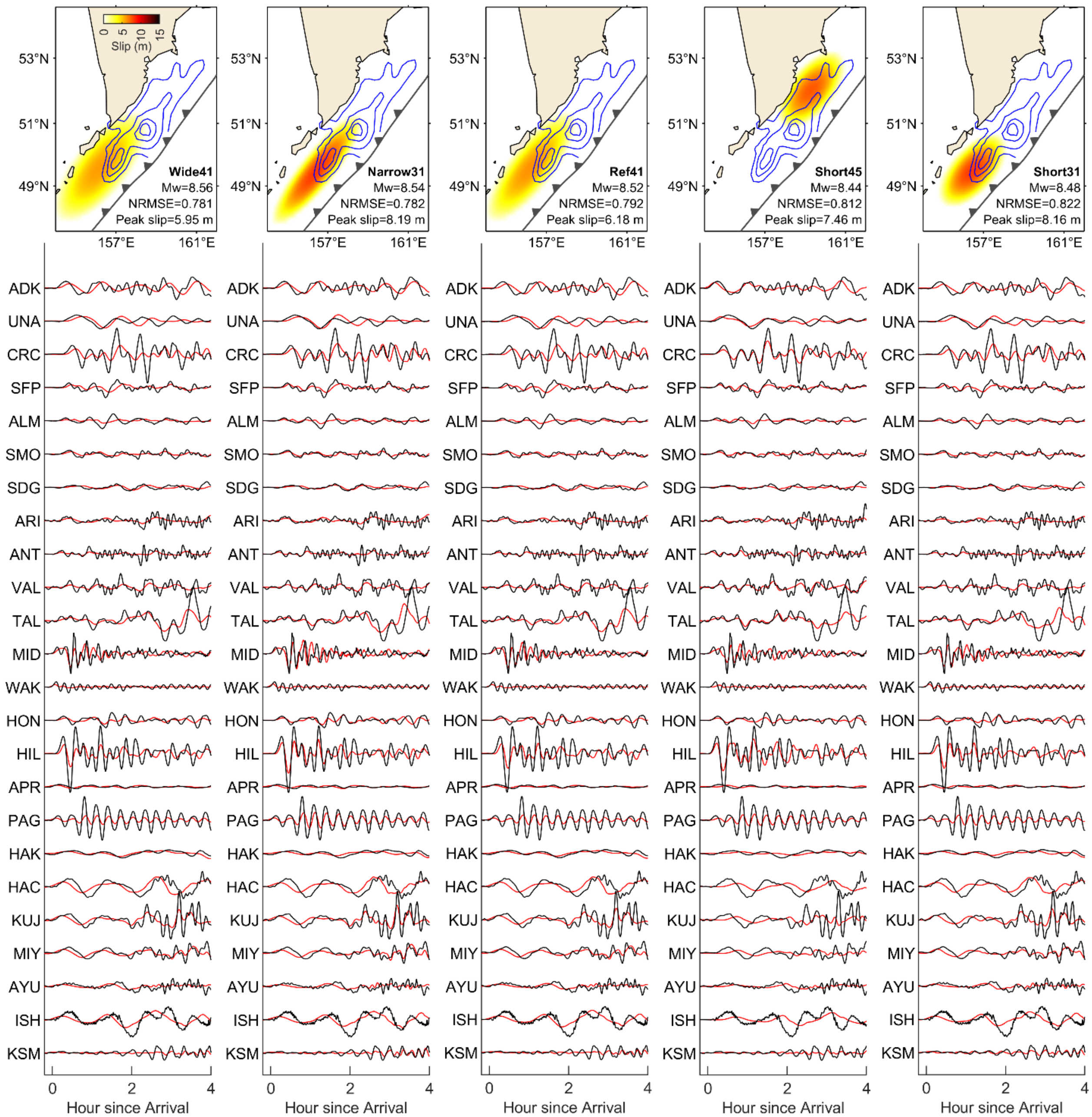
Slip (m)
0 5 10 15
53°N
51°N
49°N
157°E
161°E
Wide41
Mw=8.56
NRMSE=0.781
Peak slip=5.95 m
Narrow31
Mw=8.54
NRMSE=0.782
Peak slip=8.19 m
Ref41
Mw=8.52
NRMSE=0.792
Peak slip=6.18 m
Short45
Mw=8.44
NRMSE=0.812
Peak slip=7.46 m
Short31
Mw=8.48
NRMSE=0.822
Peak slip=8.16 m
ADK
UNA
CRC
SFP
ALM
SMO
SDG
ARI
ANT
VAL
TAL
MID
WAK
HON
HIL
APR
PAG
HAK
HAC
KUJ
MIY
AYU
ISH
KSM
0
2
4
Hour since Arrival

Fig. S5 (cont.).

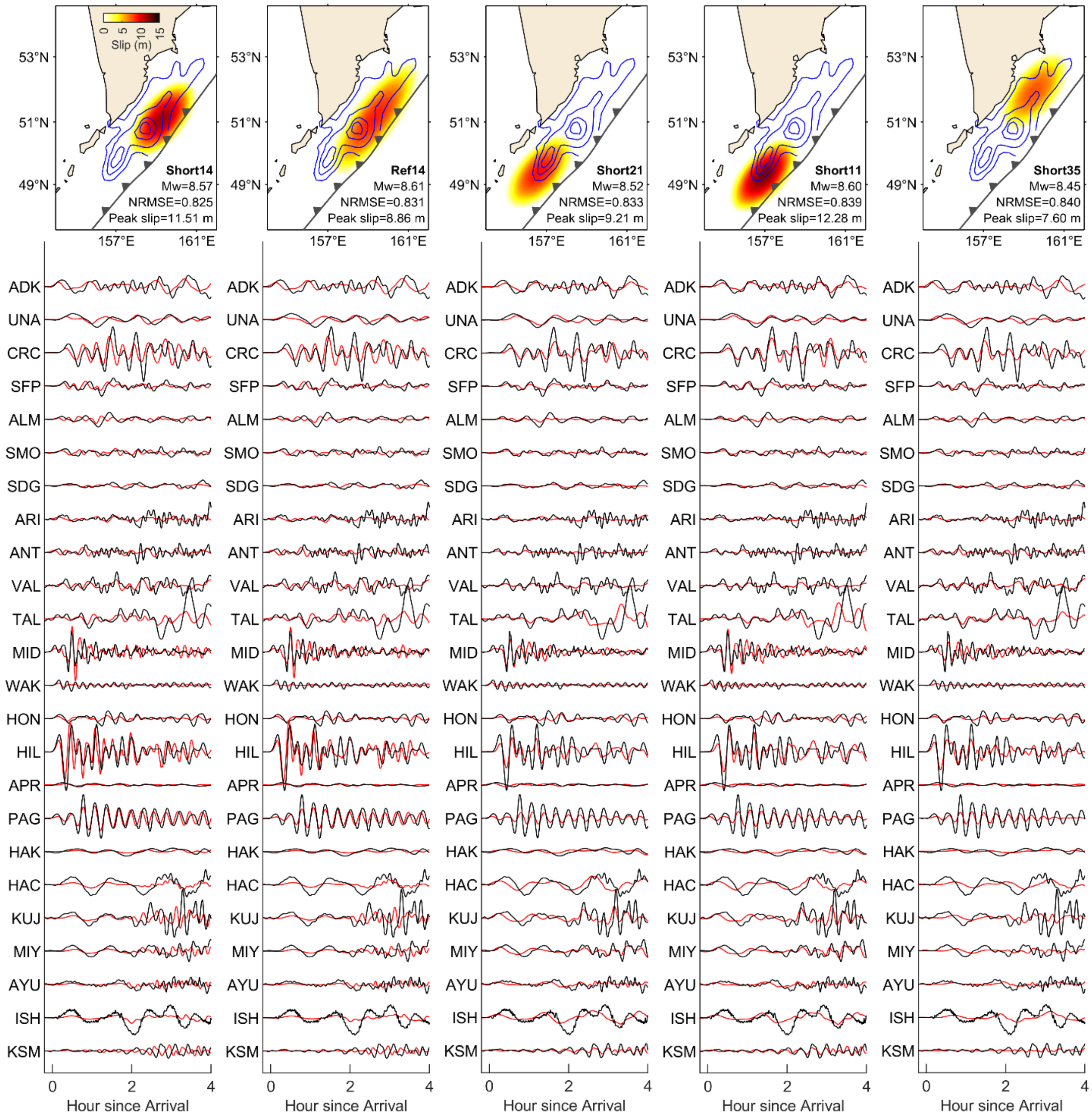

Slip (m)
0
5
10
15
53°N
51°N
49°N
157°E
161°E
Short14
Mw=8.57
NRMSE=0.825
Peak slip=11.51 m
Ref14
Mw=8.61
NRMSE=0.831
Peak slip=8.86 m
Short21
Mw=8.52
NRMSE=0.833
Peak slip=9.21 m
Short11
Mw=8.60
NRMSE=0.839
Peak slip=12.28 m
Short35
Mw=8.45
NRMSE=0.840
Peak slip=7.60 m
ADK
UNA
CRC
SFP
ALM
SMO
SDG
ARI
ANT
VAL
TAL
MID
WAK
HON
HIL
APR
PAG
HAK
HAC
KUJ
MIY
AYU
ISH
KSM
0
2
4
Hour since Arrival

Fig. S5 (cont.).

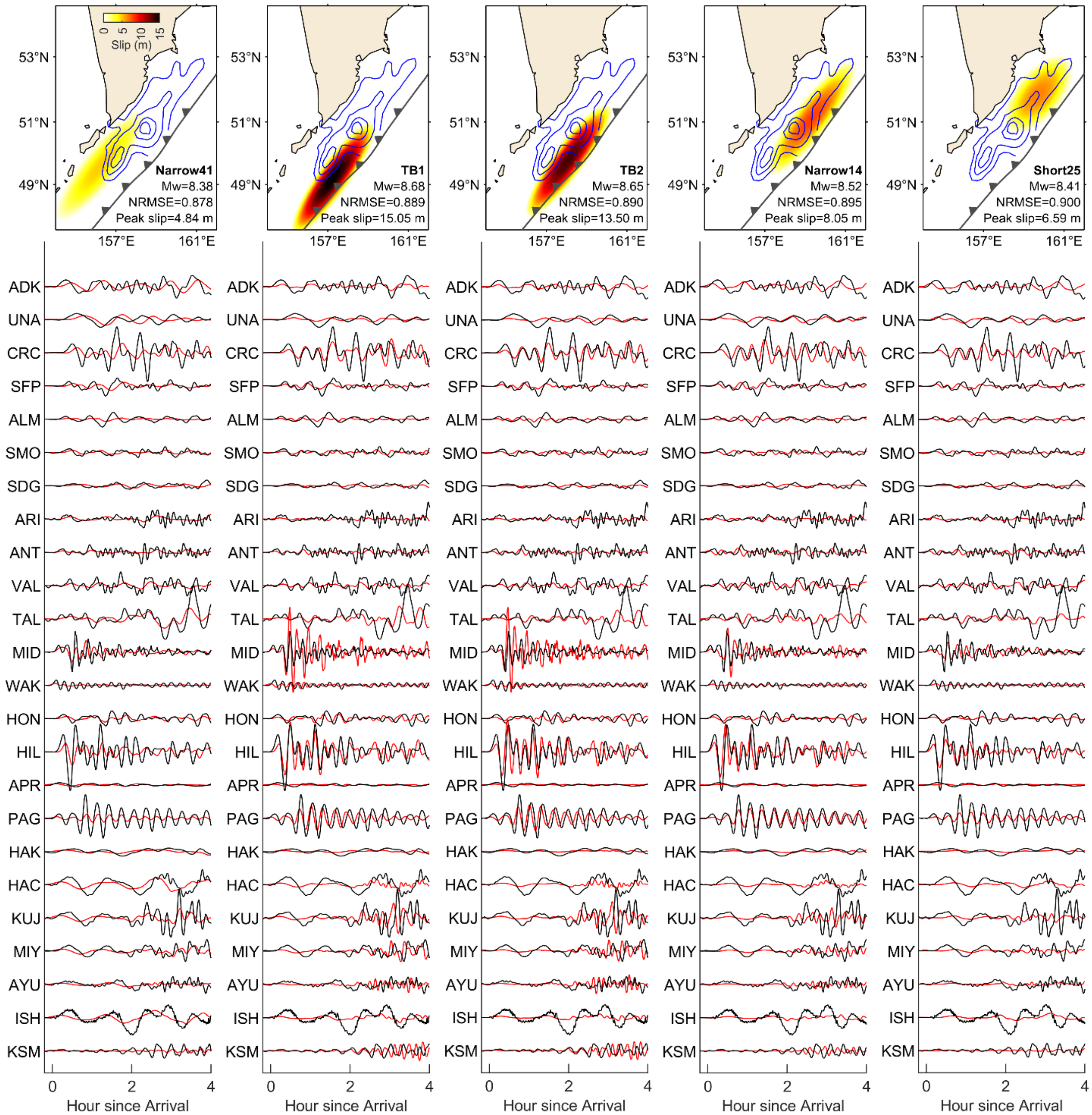
Slip (m)
0 5 10 15
53°N
51°N
49°N
157°E 161°E
Narrow41
Mw=8.38
NRMSE=0.878
Peak slip=4.84 m
TB1
Mw=8.68
NRMSE=0.889
Peak slip=15.05 m
TB2
Mw=8.65
NRMSE=0.890
Peak slip=13.50 m
Narrow14
Mw=8.52
NRMSE=0.895
Peak slip=8.05 m
Short25
Mw=8.41
NRMSE=0.900
Peak slip=6.59 m
ADK
UNA
CRC
SFP
ALM
SMO
SDG
ARI
ANT
VAL
TAL
MID
WAK
HON
HIL
APR
PAG
HAK
HAC
KUJ
MIY
AYU
ISH
KSM
0 2 4
Hour since Arrival

Fig. S5 (cont.).

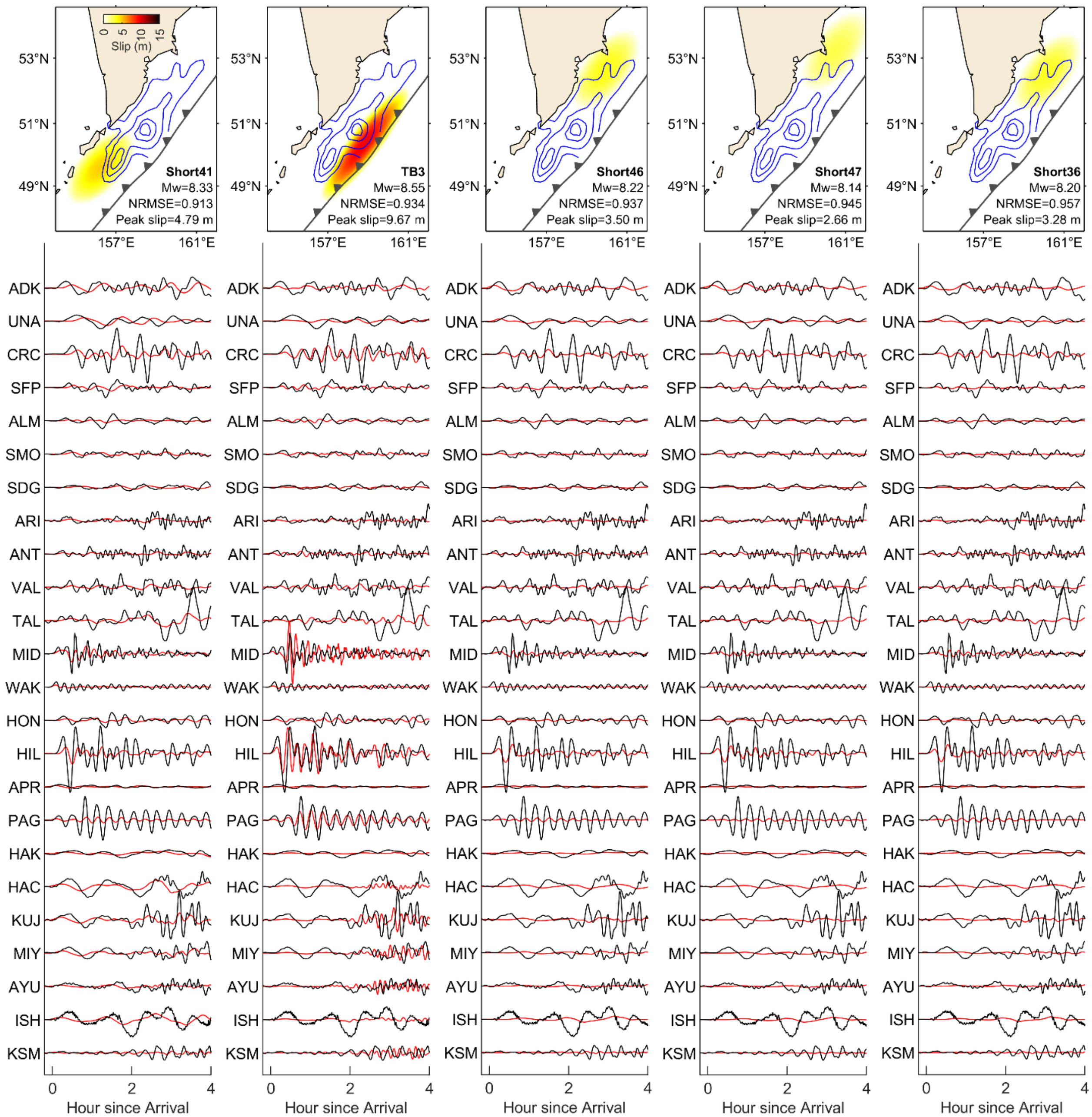
Slip (m)
0
5
10
15
53°N
51°N
49°N
157°E
161°E
Short41
Mw=8.33
NRMSE=0.913
Peak slip=4.79 m
TB3
Mw=8.55
NRMSE=0.934
Peak slip=9.67 m
Short46
Mw=8.22
NRMSE=0.937
Peak slip=3.50 m
Short47
Mw=8.14
NRMSE=0.945
Peak slip=2.66 m
Short36
Mw=8.20
NRMSE=0.957
Peak slip=3.28 m
ADK
UNA
CRC
SFP
ALM
SMO
SDG
ARI
ANT
VAL
TAL
MID
WAK
HON
HIL
APR
PAG
HAK
HAC
KUJ
MIY
AYU
ISH
KSM
0
2
4
Hour since Arrival

Fig. S5 (cont.).

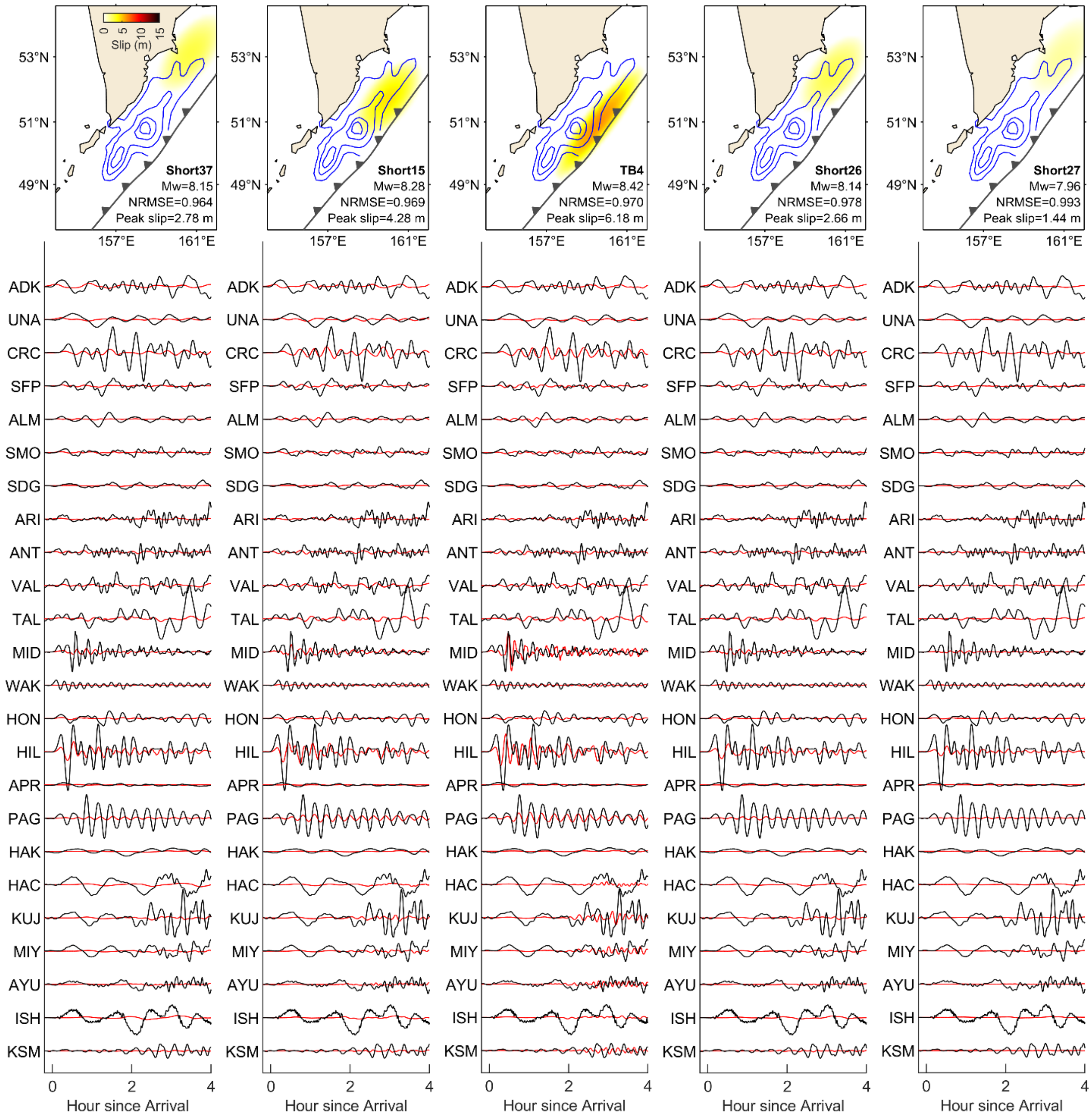

Slip (m)
0 5 10 15
Short37
Mw=8.15
NRMSE=0.964
Peak slip=2.78 m
Short15
Mw=8.28
NRMSE=0.969
Peak slip=4.28 m
TB4
Mw=8.42
NRMSE=0.970
Peak slip=6.18 m
Short26
Mw=8.14
NRMSE=0.978
Peak slip=2.66 m
Short27
Mw=7.96
NRMSE=0.993
Peak slip=1.44 m
53°N
51°N
49°N
157°E
161°E
ADK
UNA
CRC
SFP
ALM
SMO
SDG
ARI
ANT
VAL
TAL
MID
WAK
HON
HIL
APR
PAG
HAK
HAC
KUJ
MIY
AYU
ISH
KSM
0
2
4
Hour since Arrival

Fig. S5 (cont.).

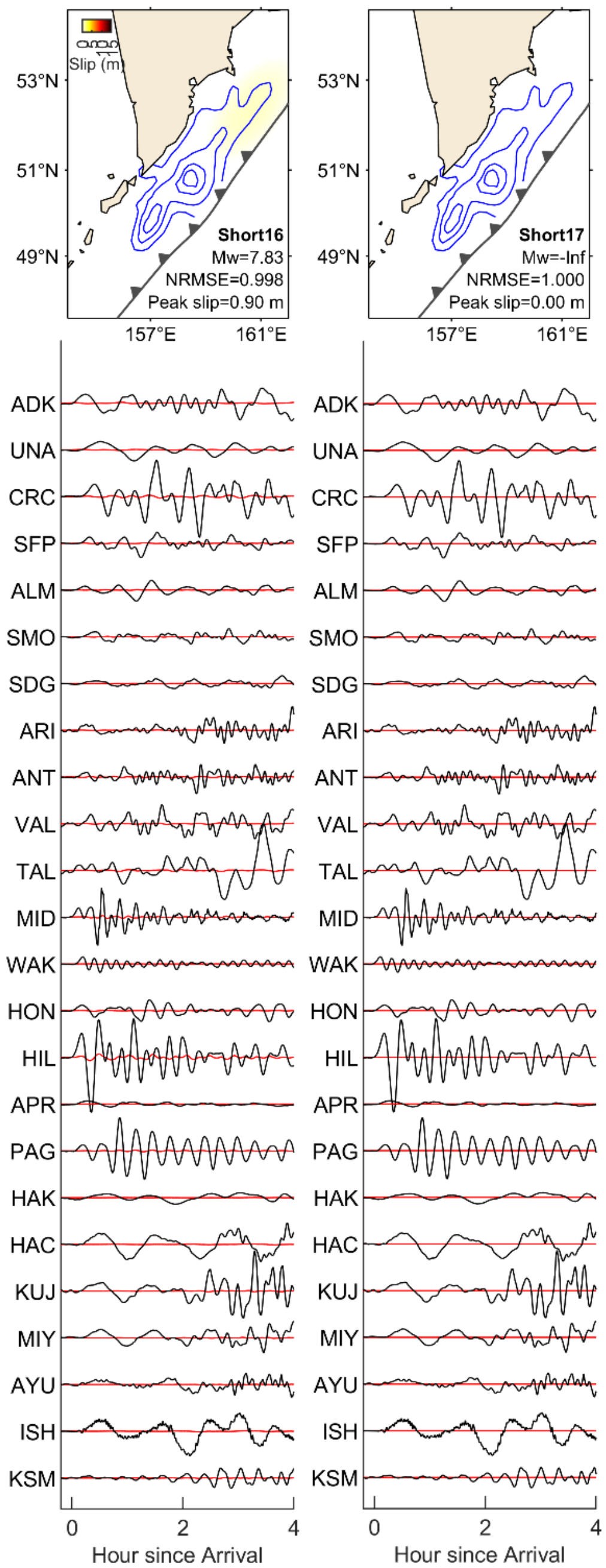
Slip (m)
Short16
Mw=7.83
NRMSE=0.998
Peak slip=0.90 m
Short17
Mw=-Inf
NRMSE=1.000
Peak slip=0.00 m
53°N
51°N
49°N
157°E
161°E
ADK
UNA
CRC
SFP
ALM
SMO
SDG
ARI
ANT
VAL
TAL
MID
WAK
HON
HIL
APR
PAG
HAK
HAC
KUJ
MIY
AYU
ISH
KSM
0
2
4
Hour since Arrival

Fig. S5 (cont.).

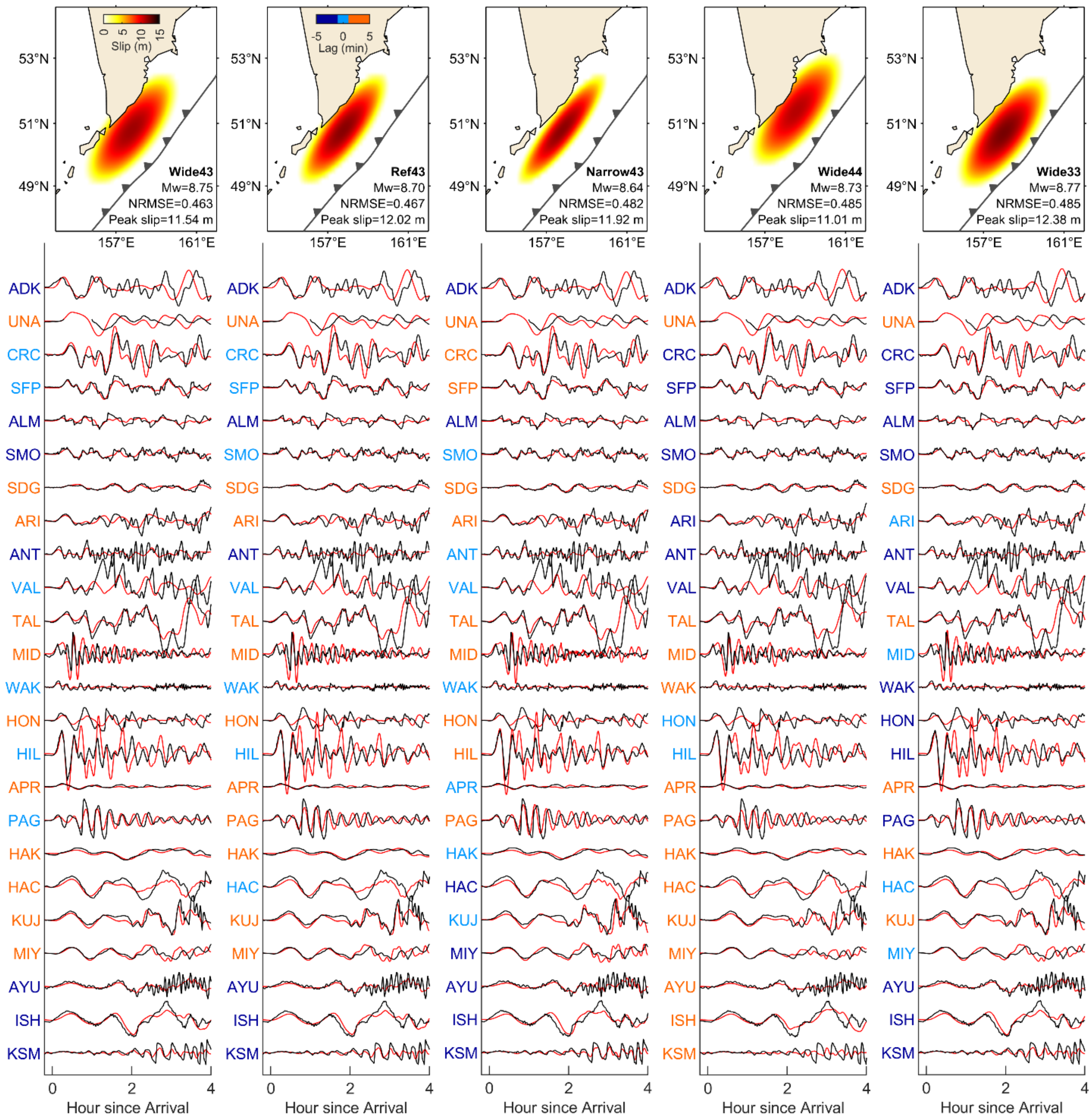

Fig. S6. Comparison between observed 1952 tsunami waveforms and predictions from 92 basic slip models. Black and red lines are observed and model-predicted waveforms, respectively. For each model, the upper panel shows the moment magnitude (Mw), Normalized root-mean-square error (NRMSE) of fit, and peak slip. The color of station names indicates the model-predicted travel-time difference (Δt_{MM}). The vertical spacing between stations is 1.5 m and provides a reference scale for tsunami amplitude.

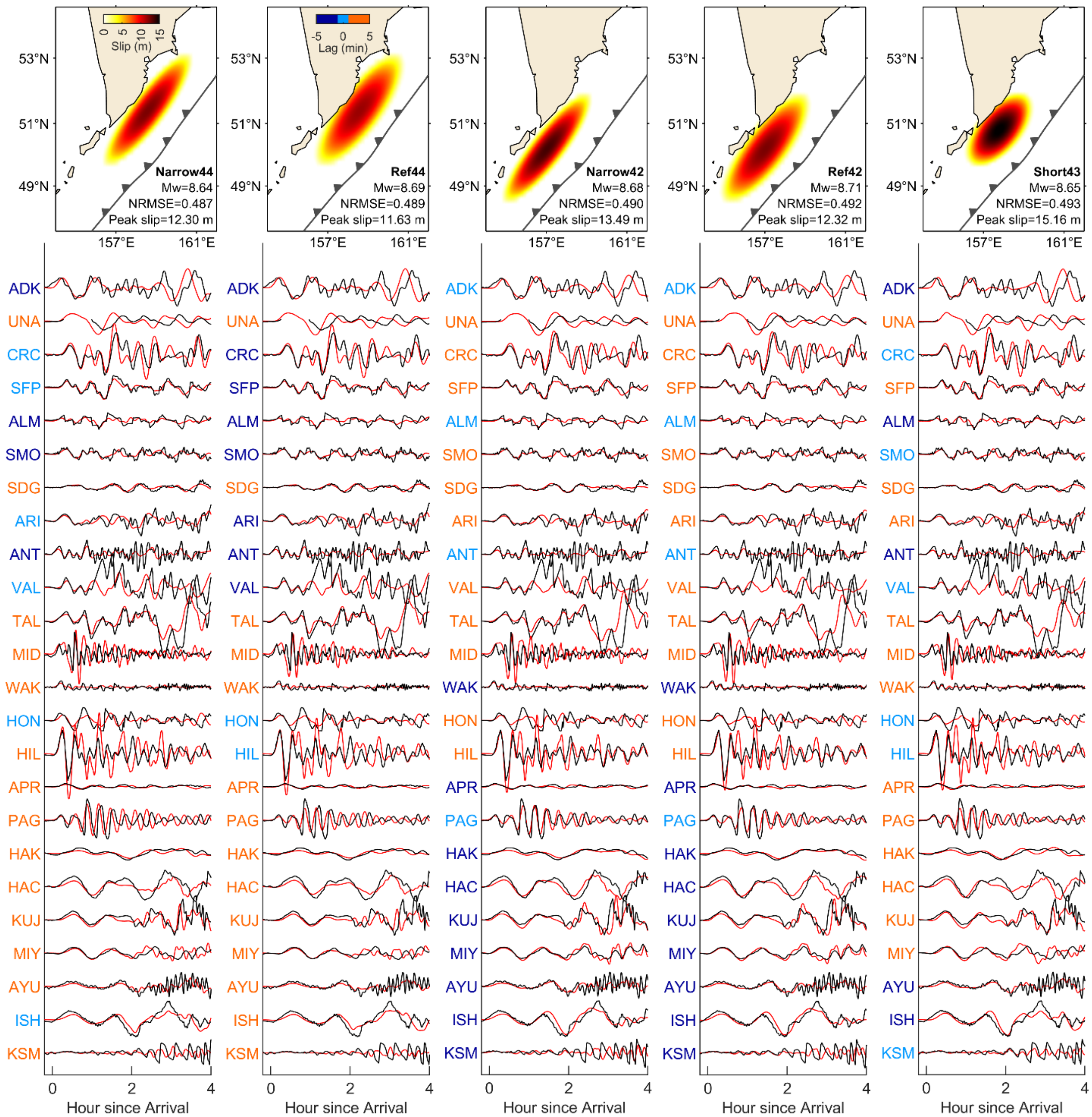

Slip (m)
0 5 10 15
Lag (min)
-5 0 5
53°N
51°N
49°N
157°E
161°E
Narrow44
Mw=8.64
NRMSE=0.487
Peak slip=12.30 m
Ref44
Mw=8.69
NRMSE=0.489
Peak slip=11.63 m
Narrow42
Mw=8.68
NRMSE=0.490
Peak slip=13.49 m
Ref42
Mw=8.71
NRMSE=0.492
Peak slip=12.32 m
Short43
Mw=8.65
NRMSE=0.493
Peak slip=15.16 m
ADK
UNA
CRC
SFP
ALM
SMO
SDG
ARI
ANT
VAL
TAL
MID
WAK
HON
HIL
APR
PAG
HAK
HAC
KUJ
MIY
AYU
ISH
KSM
0
2
4
Hour since Arrival

Fig. S6 (cont.).

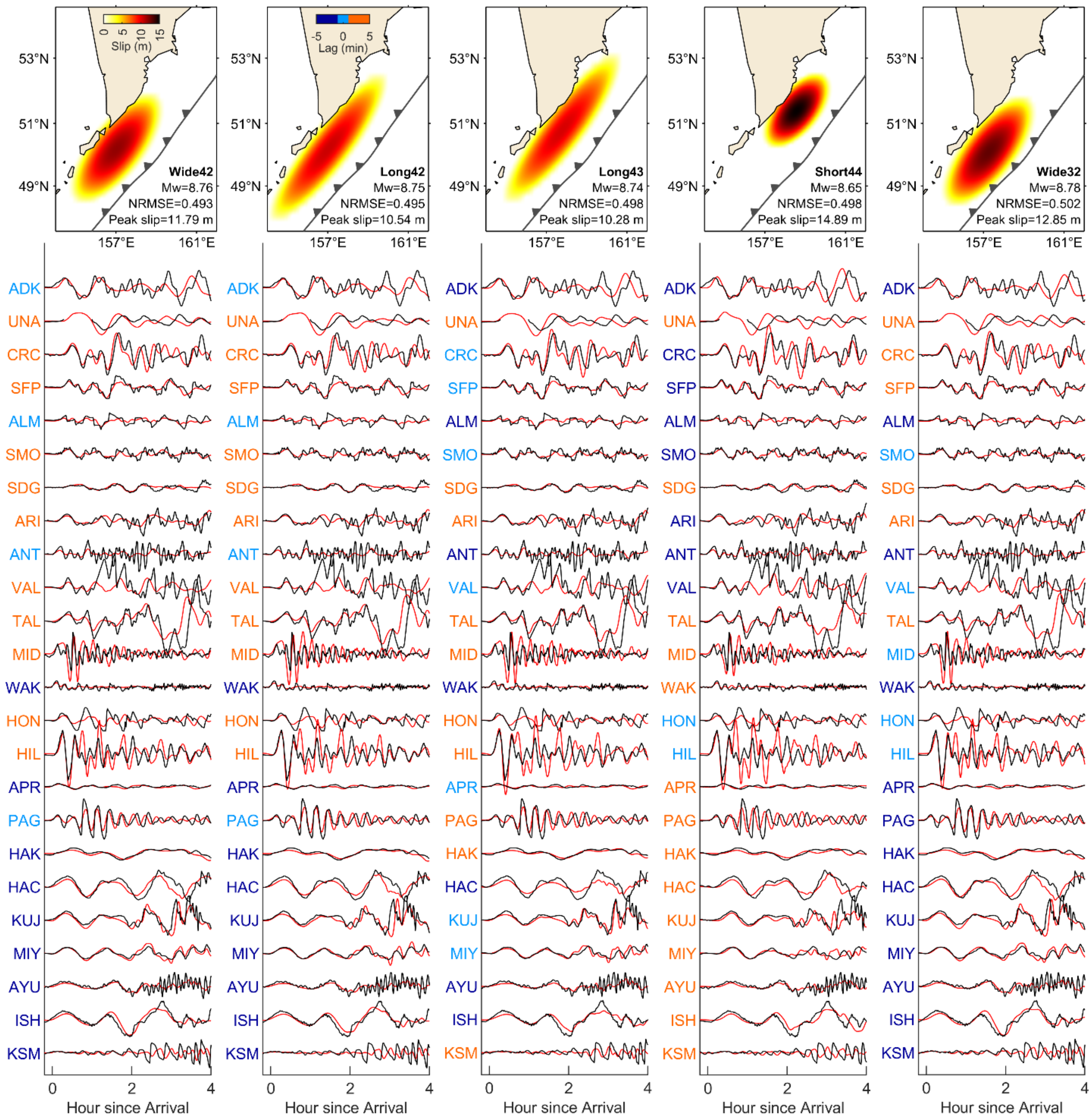

Slip (m)
Lag (min)
53°N
51°N
49°N
157°E
161°E
Wide42
Mw=8.76
NRMSE=0.493
Peak slip=11.79 m
Long42
Mw=8.75
NRMSE=0.495
Peak slip=10.54 m
Long43
Mw=8.74
NRMSE=0.498
Peak slip=10.28 m
Short44
Mw=8.65
NRMSE=0.498
Peak slip=14.89 m
Wide32
Mw=8.78
NRMSE=0.502
Peak slip=12.85 m
ADK
UNA
CRC
SFP
ALM
SMO
SDG
ARI
ANT
VAL
TAL
MID
WAK
HON
HIL
APR
PAG
HAK
HAC
KUJ
MIY
AYU
ISH
KSM
Hour since Arrival

Fig. S6 (cont.).

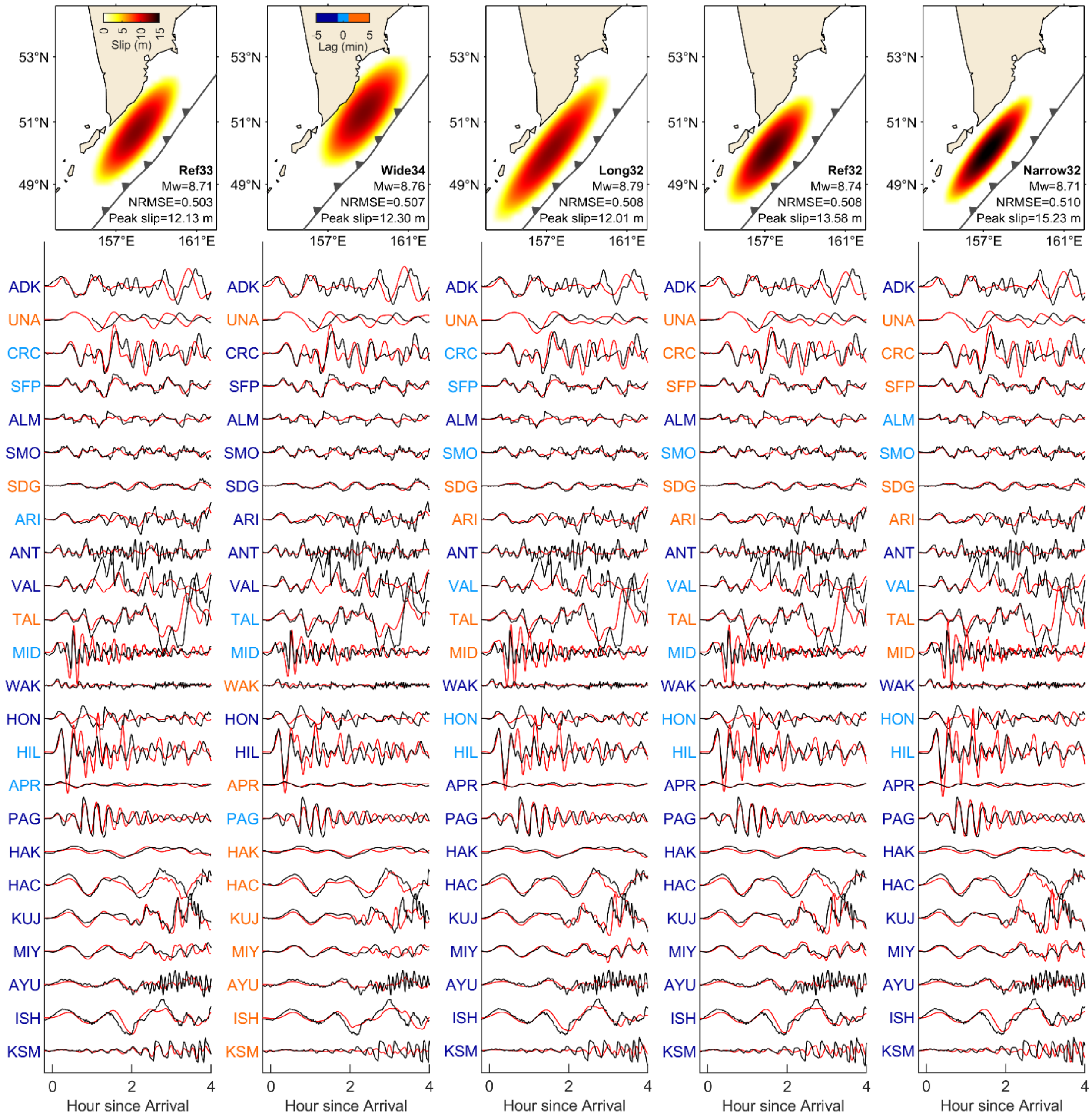

Slip (m)
Lag (min)
53°N
51°N
49°N
157°E
161°E
Ref33
Mw=8.71
NRMSE=0.503
Peak slip=12.13 m
Wide34
Mw=8.76
NRMSE=0.507
Peak slip=12.30 m
Long32
Mw=8.79
NRMSE=0.508
Peak slip=12.01 m
Ref32
Mw=8.74
NRMSE=0.508
Peak slip=13.58 m
Narrow32
Mw=8.71
NRMSE=0.510
Peak slip=15.23 m
ADK
UNA
CRC
SFP
ALM
SMO
SDG
ARI
ANT
VAL
TAL
MID
WAK
HON
HIL
APR
PAG
HAK
HAC
KUJ
MIY
AYU
ISH
KSM
Hour since Arrival

Fig. S6 (cont.).

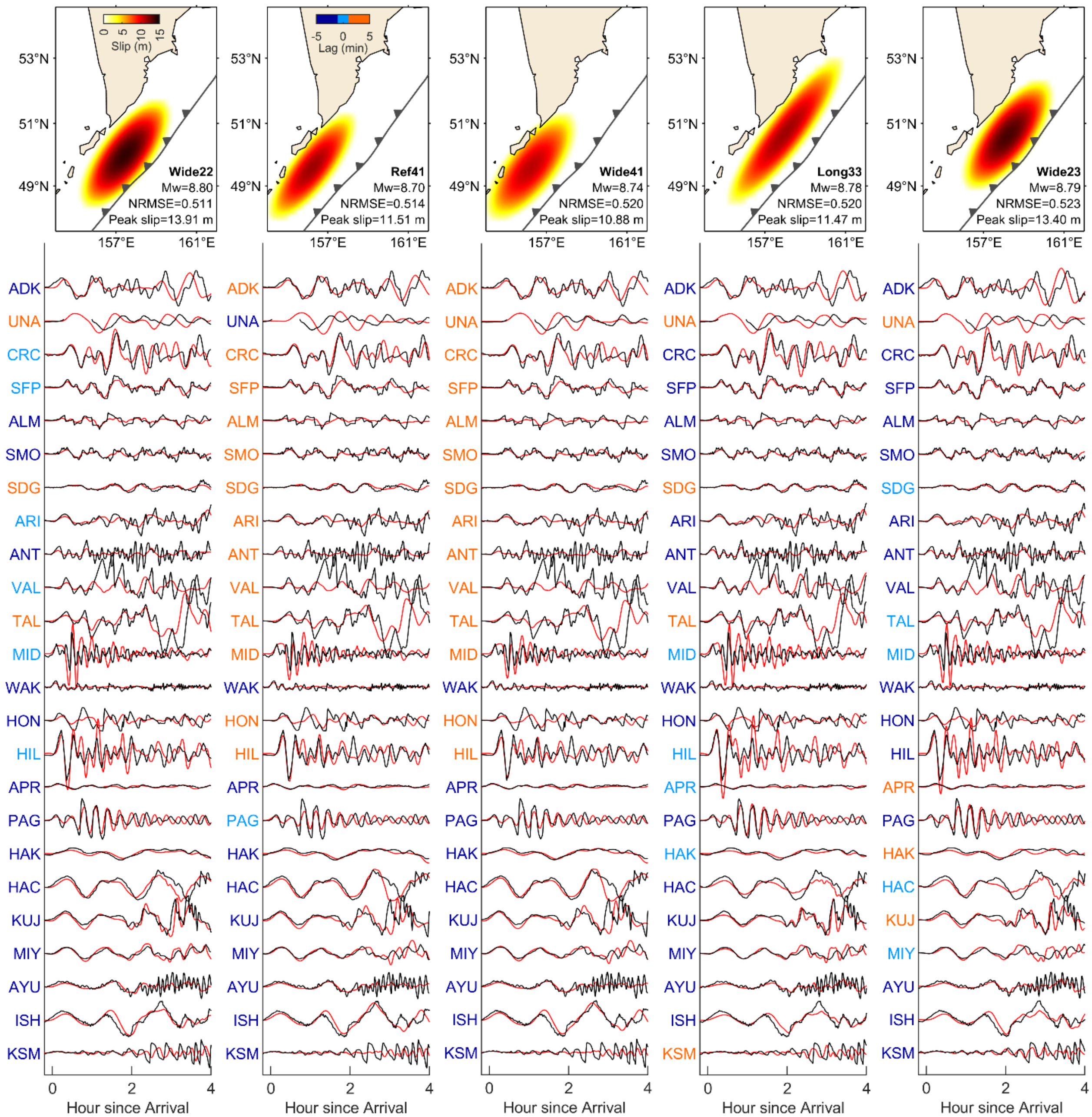

Slip (m)
Lag (min)
53°N
51°N
49°N
157°E
161°E
Wide22
Mw=8.80
NRMSE=0.511
Peak slip=13.91 m
Ref41
Mw=8.70
NRMSE=0.514
Peak slip=11.51 m
Wide41
Mw=8.74
NRMSE=0.520
Peak slip=10.88 m
Long33
Mw=8.78
NRMSE=0.520
Peak slip=11.47 m
Wide23
Mw=8.79
NRMSE=0.523
Peak slip=13.40 m
ADK
UNA
CRC
SFP
ALM
SMO
SDG
ARI
ANT
VAL
TAL
MID
WAK
HON
HIL
APR
PAG
HAK
HAC
KUJ
MIY
AYU
ISH
KSM
Hour since Arrival

Fig. S6 (cont.).

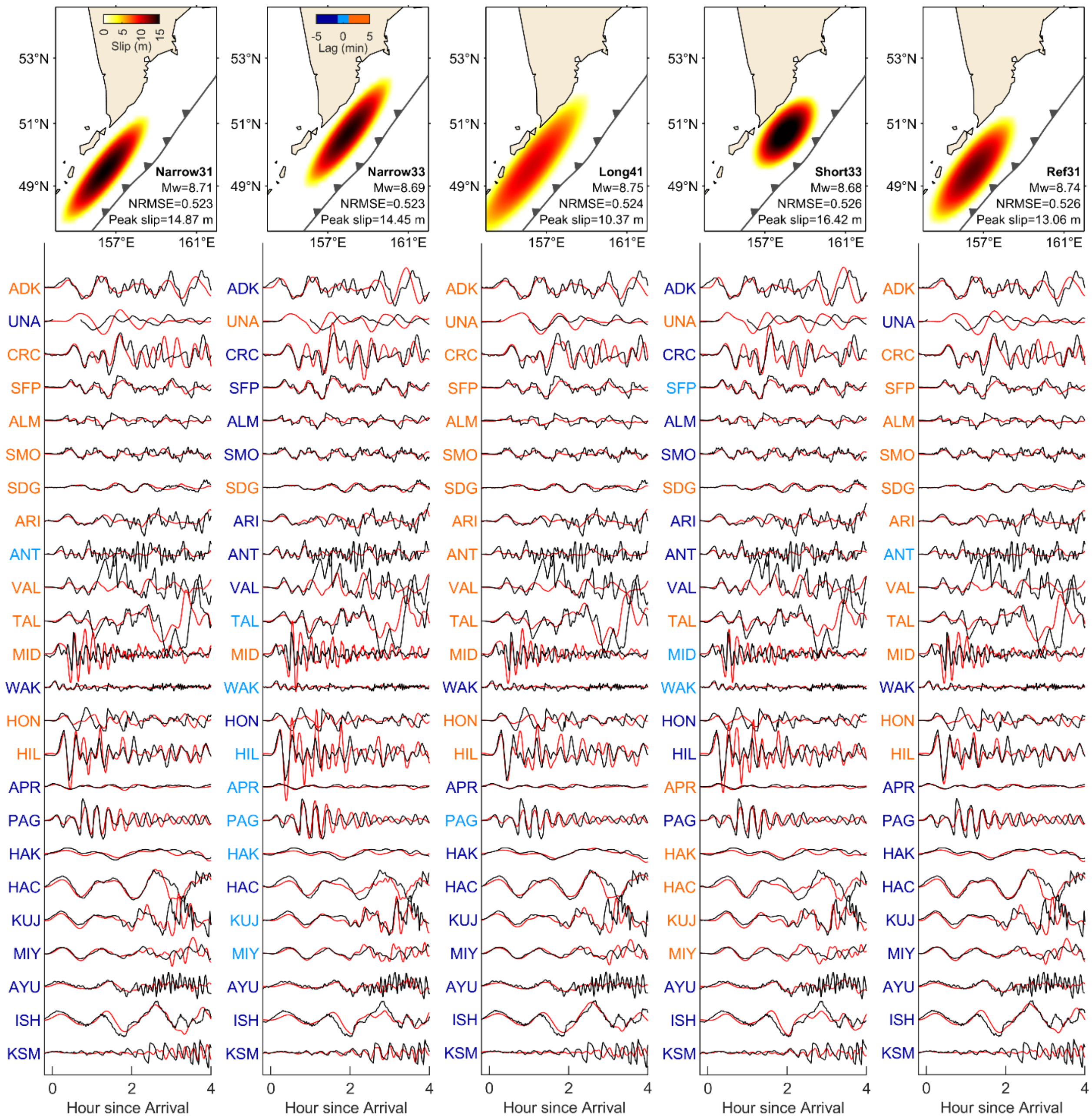

Slip (m)
0 5 10 15
Lag (min)
-5 0 5
53°N
51°N
49°N
157°E
161°E
Narrow31
Mw=8.71
NRMSE=0.523
Peak slip=14.87 m
Narrow33
Mw=8.69
NRMSE=0.523
Peak slip=14.45 m
Long41
Mw=8.75
NRMSE=0.524
Peak slip=10.37 m
Short33
Mw=8.68
NRMSE=0.526
Peak slip=16.42 m
Ref31
Mw=8.74
NRMSE=0.526
Peak slip=13.06 m
ADK
UNA
CRC
SFP
ALM
SMO
SDG
ARI
ANT
VAL
TAL
MID
WAK
HON
HIL
APR
PAG
HAK
HAC
KUJ
MIY
AYU
ISH
KSM
0 2 4
Hour since Arrival

Fig. S6 (cont.).

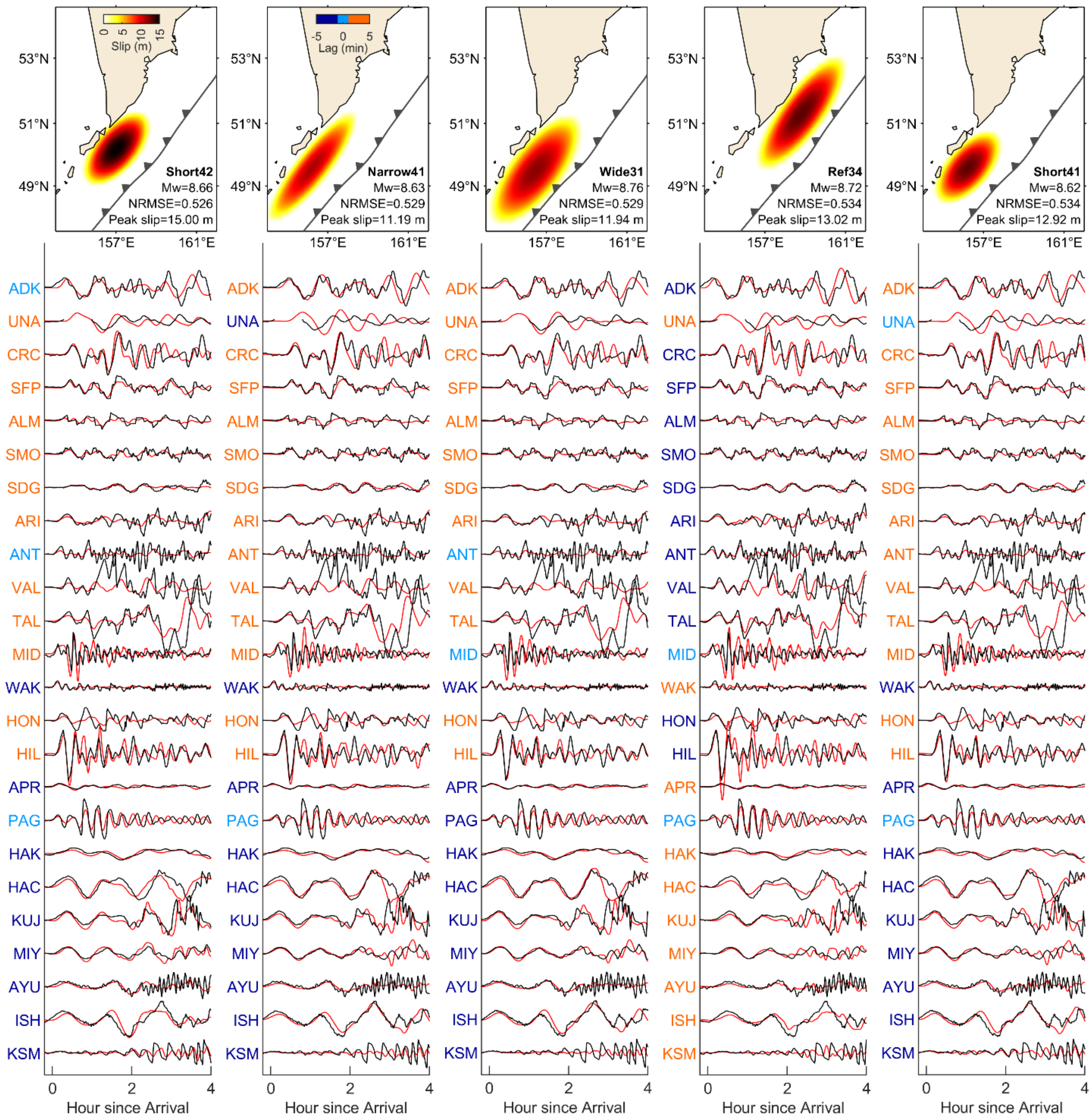

Slip (m)
0 5 10 15
Lag (min)
-5 0 5
53°N
51°N
49°N
157°E
161°E
Short42
Mw=8.66
NRMSE=0.526
Peak slip=15.00 m
Narrow41
Mw=8.63
NRMSE=0.529
Peak slip=11.19 m
Wide31
Mw=8.76
NRMSE=0.529
Peak slip=11.94 m
Ref34
Mw=8.72
NRMSE=0.534
Peak slip=13.02 m
Short41
Mw=8.62
NRMSE=0.534
Peak slip=12.92 m
ADK
UNA
CRC
SFP
ALM
SMO
SDG
ARI
ANT
VAL
TAL
MID
WAK
HON
HIL
APR
PAG
HAK
HAC
KUJ
MIY
AYU
ISH
KSM
0 2 4
Hour since Arrival

Fig. S6 (cont.).

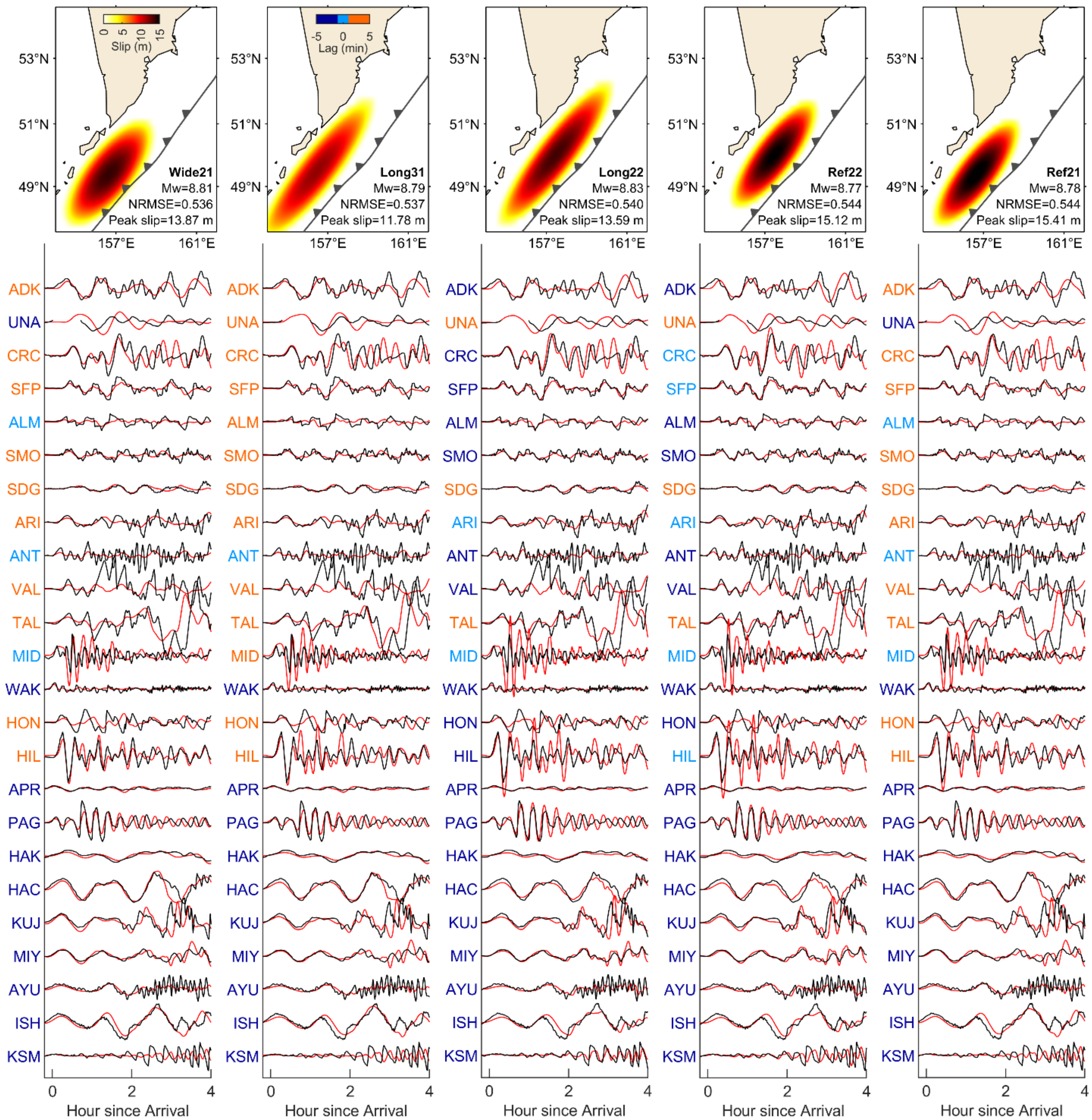
Slip (m)
Lag (min)
53°N
51°N
49°N
157°E
161°E
Wide21
Mw=8.81
NRMSE=0.536
Peak slip=13.87 m
Long31
Mw=8.79
NRMSE=0.537
Peak slip=11.78 m
Long22
Mw=8.83
NRMSE=0.540
Peak slip=13.59 m
Ref22
Mw=8.77
NRMSE=0.544
Peak slip=15.12 m
Ref21
Mw=8.78
NRMSE=0.544
Peak slip=15.41 m
ADK
UNA
CRC
SFP
ALM
SMO
SDG
ARI
ANT
VAL
TAL
MID
WAK
HON
HIL
APR
PAG
HAK
HAC
KUJ
MIY
AYU
ISH
KSM
Hour since Arrival

Fig. S6 (cont.).

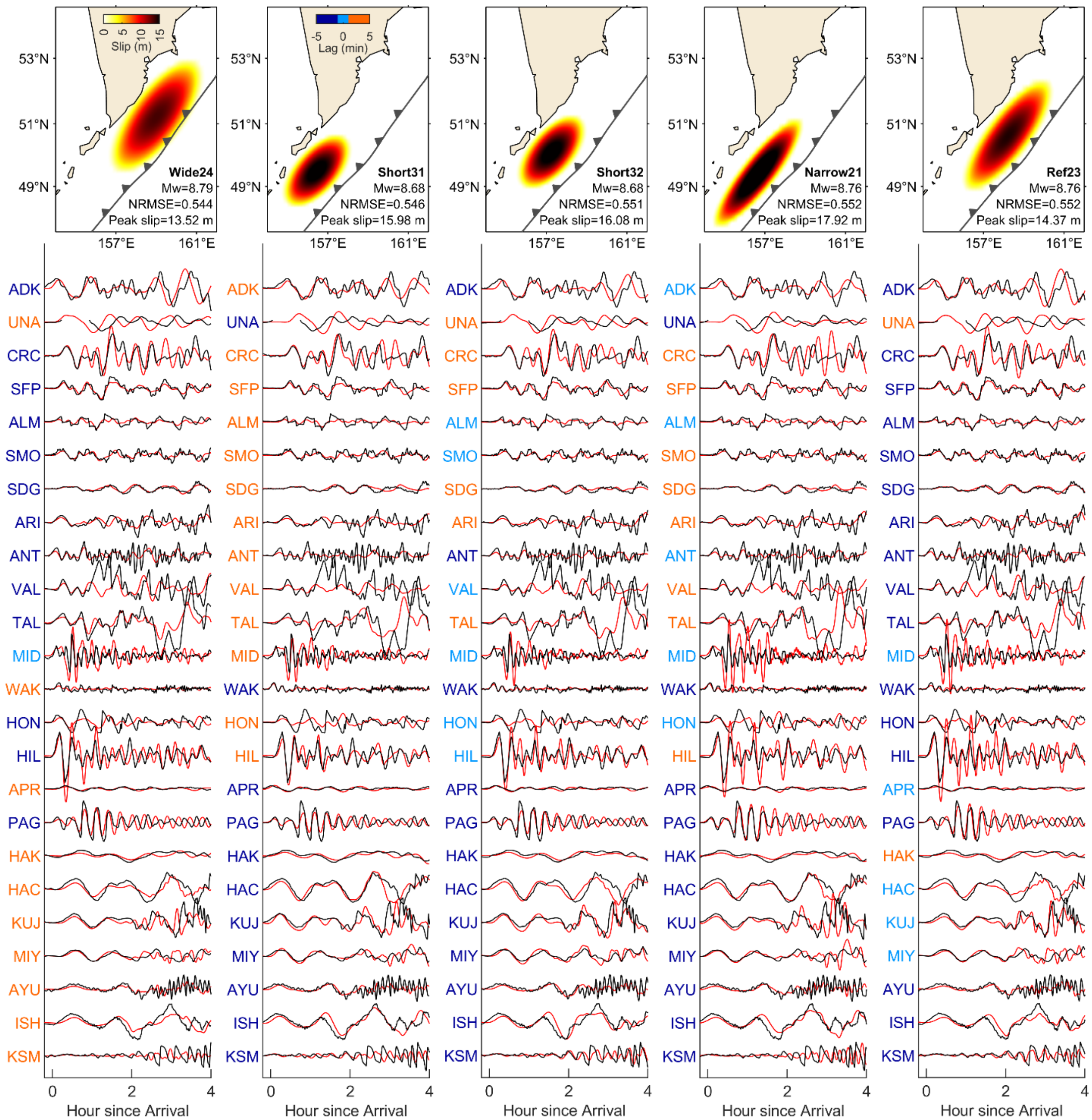

Slip (m)
0 5 10 15
Lag (min)
-5 0 5
53°N
51°N
49°N
157°E
161°E
Wide24
Mw=8.79
NRMSE=0.544
Peak slip=13.52 m
Short31
Mw=8.68
NRMSE=0.546
Peak slip=15.98 m
Short32
Mw=8.68
NRMSE=0.551
Peak slip=16.08 m
Narrow21
Mw=8.76
NRMSE=0.552
Peak slip=17.92 m
Ref23
Mw=8.76
NRMSE=0.552
Peak slip=14.37 m
ADK
UNA
CRC
SFP
ALM
SMO
SDG
ARI
ANT
VAL
TAL
MID
WAK
HON
HIL
APR
PAG
HAK
HAC
KUJ
MIY
AYU
ISH
KSM
0
2
4
Hour since Arrival

Fig. S6 (cont.).

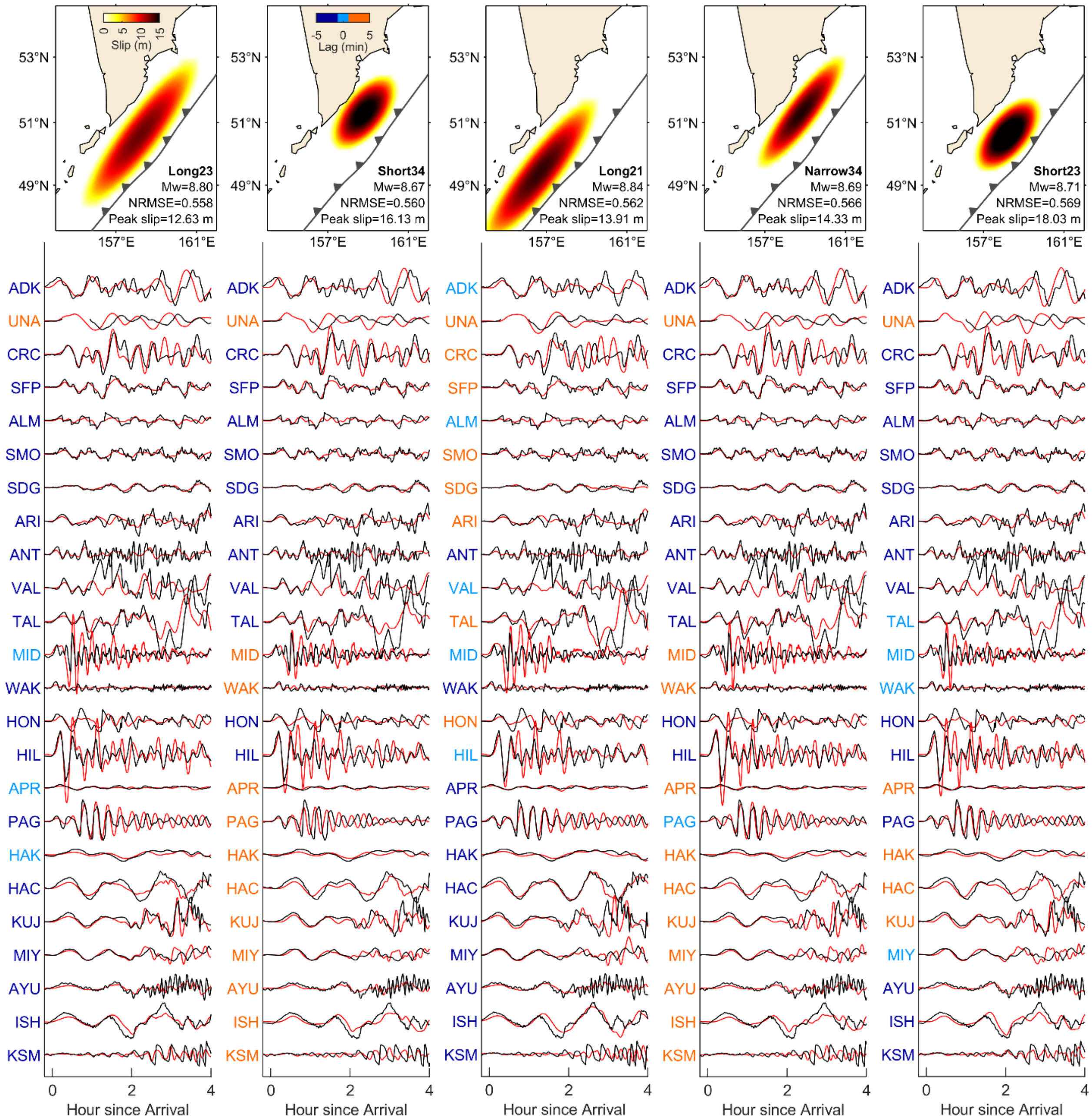

Slip (m)
0 5 10 15
Lag (min)
-5 0 5
53°N
51°N
49°N
157°E
161°E
Long23
Mw=8.80
NRMSE=0.558
Peak slip=12.63 m
Short34
Mw=8.67
NRMSE=0.560
Peak slip=16.13 m
Long21
Mw=8.84
NRMSE=0.562
Peak slip=13.91 m
Narrow34
Mw=8.69
NRMSE=0.566
Peak slip=14.33 m
Short23
Mw=8.71
NRMSE=0.569
Peak slip=18.03 m
ADK
UNA
CRC
SFP
ALM
SMO
SDG
ARI
ANT
VAL
TAL
MID
WAK
HON
HIL
APR
PAG
HAK
HAC
KUJ
MIY
AYU
ISH
KSM
0 2 4
Hour since Arrival

Fig. S6 (cont.).

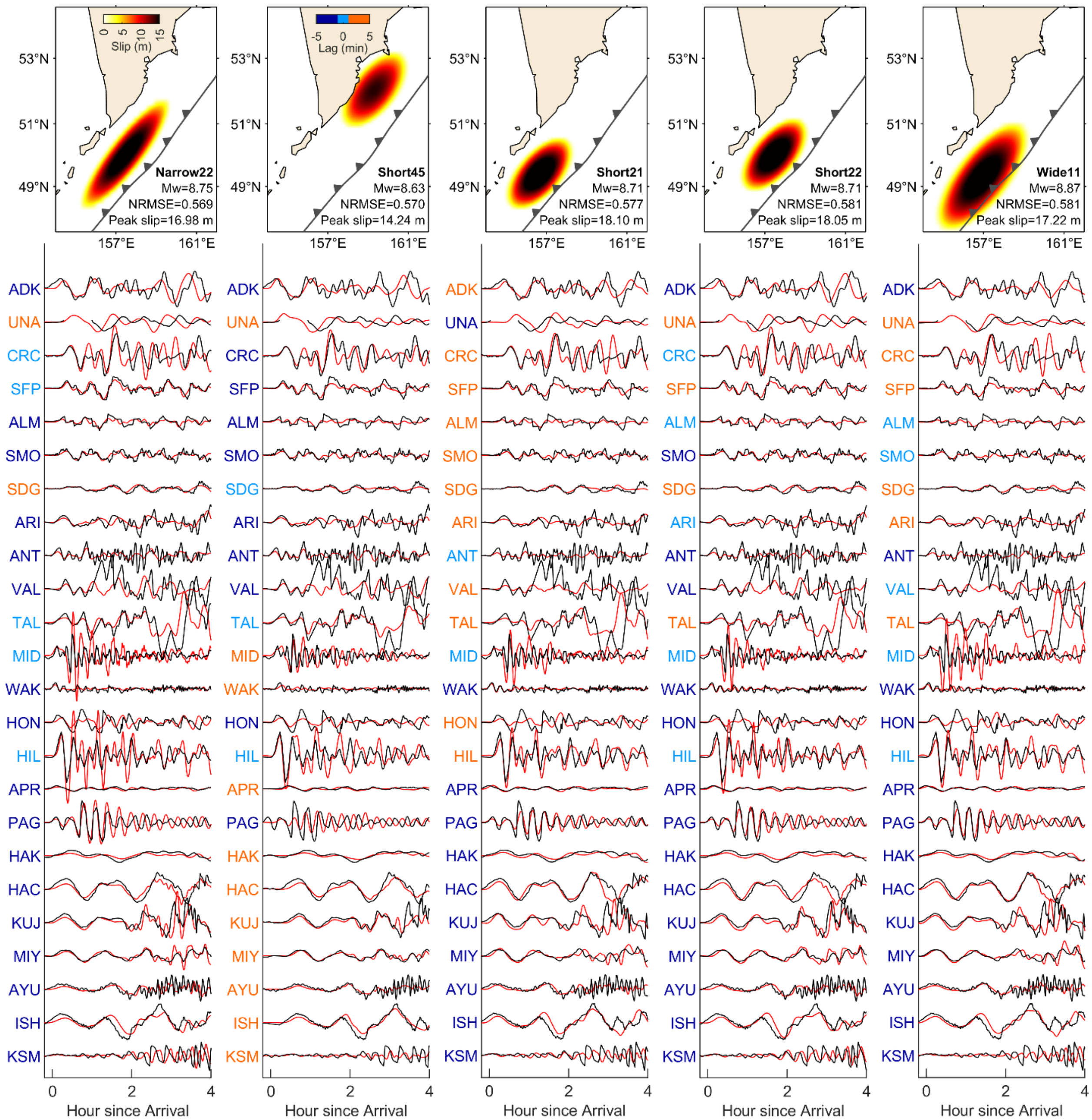

Slip (m)
Lag (min)
53°N
51°N
49°N
157°E
161°E
Narrow22
Mw=8.75
NRMSE=0.569
Peak slip=16.98 m
Short45
Mw=8.63
NRMSE=0.570
Peak slip=14.24 m
Short21
Mw=8.71
NRMSE=0.577
Peak slip=18.10 m
Short22
Mw=8.71
NRMSE=0.581
Peak slip=18.05 m
Wide11
Mw=8.87
NRMSE=0.581
Peak slip=17.22 m
ADK
UNA
CRC
SFP
ALM
SMO
SDG
ARI
ANT
VAL
TAL
MID
WAK
HON
HIL
APR
PAG
HAK
HAC
KUJ
MIY
AYU
ISH
KSM
Hour since Arrival

Fig. S6 (cont.).

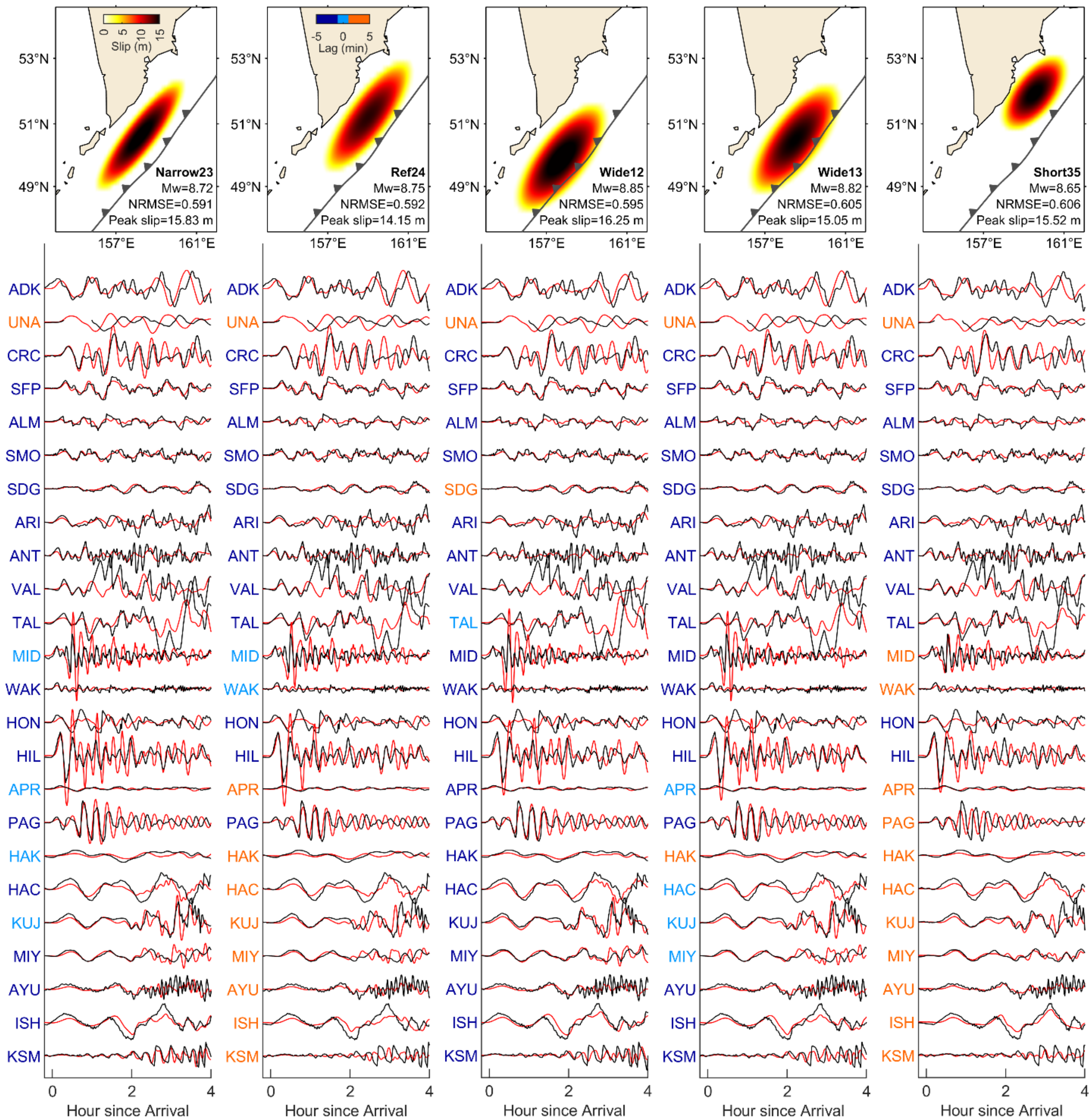

Slip (m)
0 5 10 15
Lag (min)
-5 0 5
53°N
51°N
49°N
157°E
161°E
Narrow23
Mw=8.72
NRMSE=0.591
Peak slip=15.83 m
Ref24
Mw=8.75
NRMSE=0.592
Peak slip=14.15 m
Wide12
Mw=8.85
NRMSE=0.595
Peak slip=16.25 m
Wide13
Mw=8.82
NRMSE=0.605
Peak slip=15.05 m
Short35
Mw=8.65
NRMSE=0.606
Peak slip=15.52 m
ADK
UNA
CRC
SFP
ALM
SMO
SDG
ARI
ANT
VAL
TAL
MID
WAK
HON
HIL
APR
PAG
HAK
HAC
KUJ
MIY
AYU
ISH
KSM
0 2 4
Hour since Arrival

Fig. S6 (cont.).

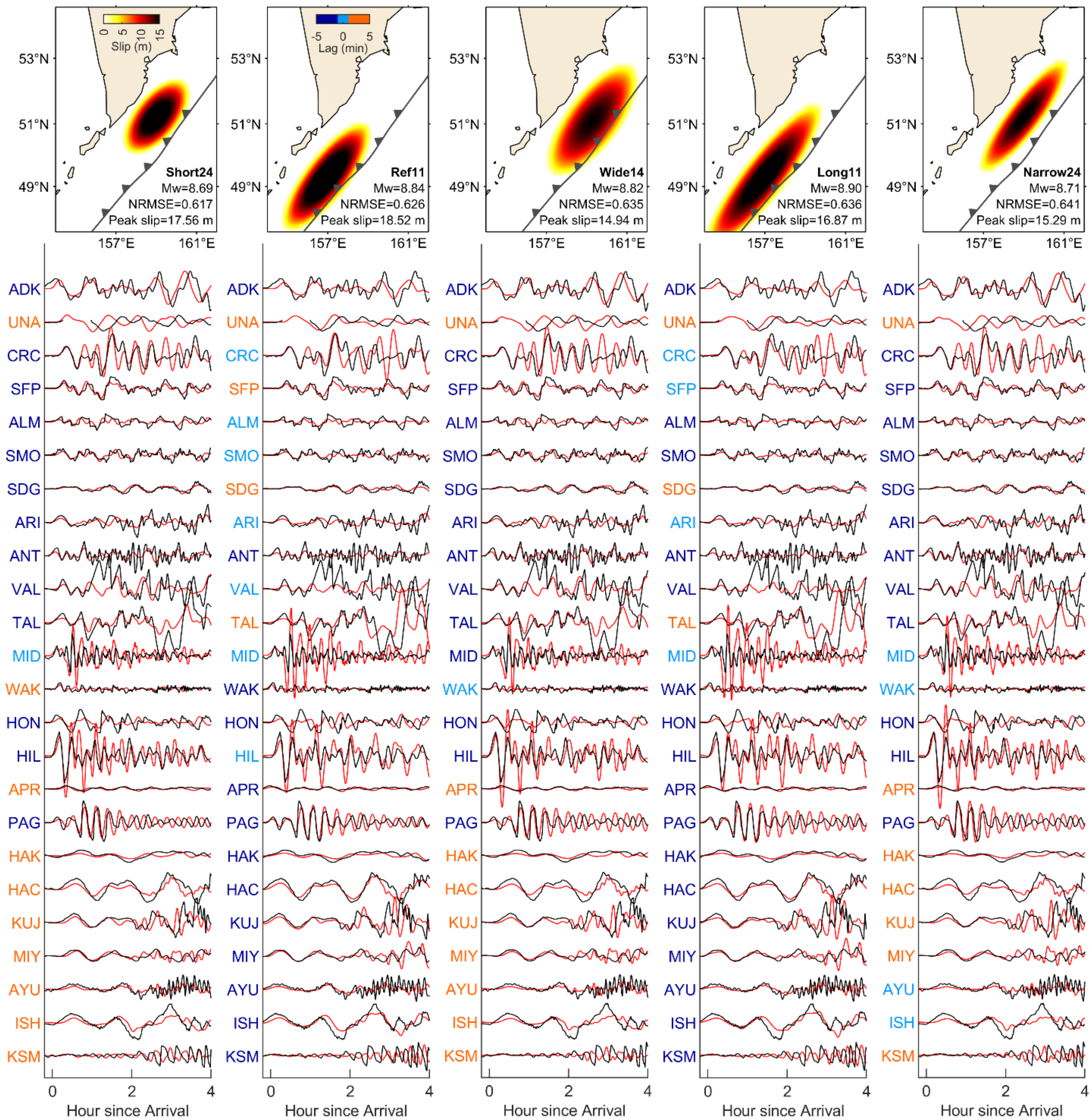

Slip (m)
Lag (min)
Short24
Mw=8.69
NRMSE=0.617
Peak slip=17.56 m
Ref11
Mw=8.84
NRMSE=0.626
Peak slip=18.52 m
Wide14
Mw=8.82
NRMSE=0.635
Peak slip=14.94 m
Long11
Mw=8.90
NRMSE=0.636
Peak slip=16.87 m
Narrow24
Mw=8.71
NRMSE=0.641
Peak slip=15.29 m
53°N
51°N
49°N
157°E
161°E
ADK
UNA
CRC
SFP
ALM
SMO
SDG
ARI
ANT
VAL
TAL
MID
WAK
HON
HIL
APR
PAG
HAK
HAC
KUJ
MIY
AYU
ISH
KSM
Hour since Arrival

Fig. S6 (cont.).

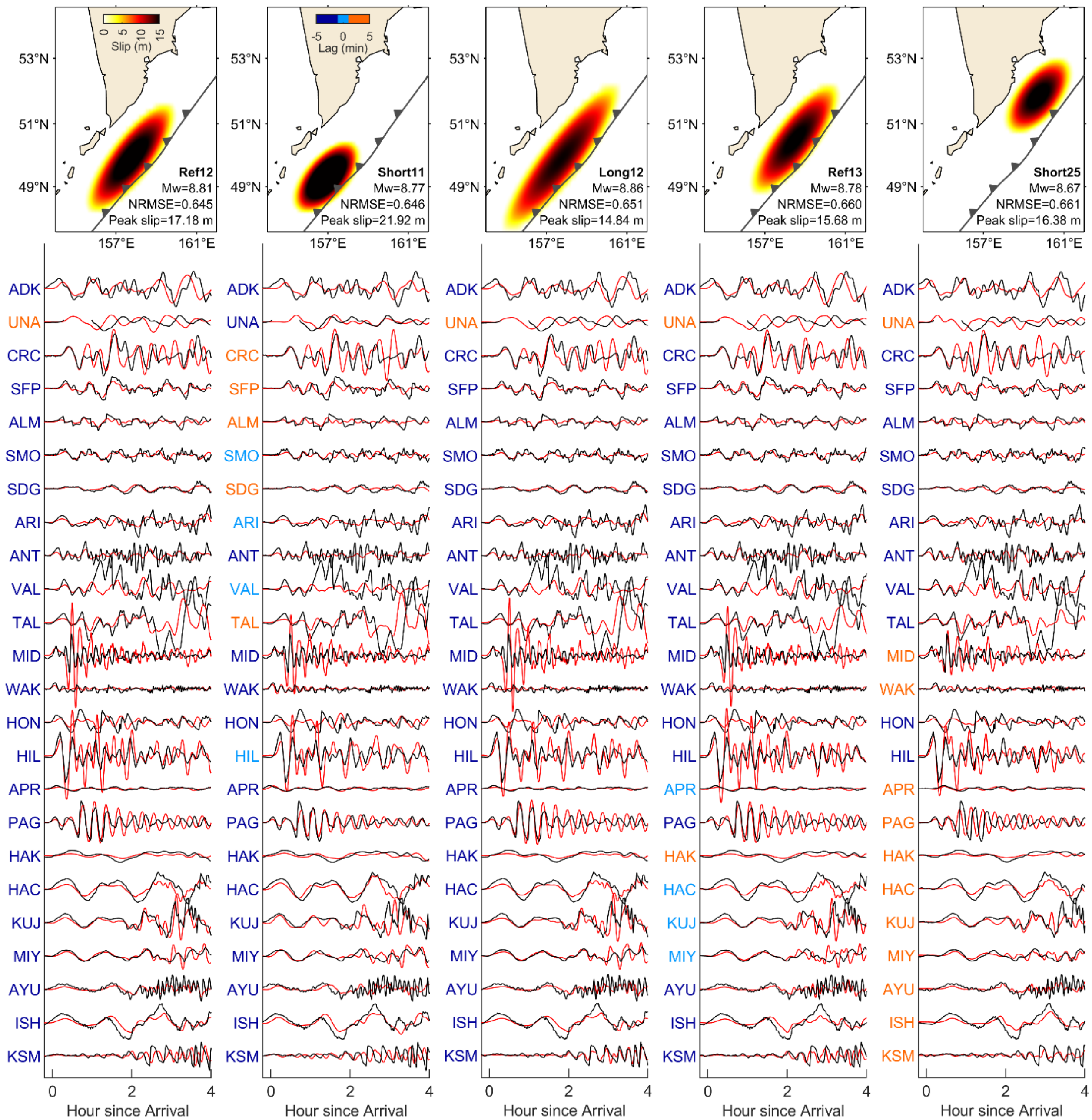

Slip (m)
0 5 10 15
Lag (min)
-5 0 5
53°N
51°N
49°N
157°E
161°E
Ref12
Mw=8.81
NRMSE=0.645
Peak slip=17.18 m
Short11
Mw=8.77
NRMSE=0.646
Peak slip=21.92 m
Long12
Mw=8.86
NRMSE=0.651
Peak slip=14.84 m
Ref13
Mw=8.78
NRMSE=0.660
Peak slip=15.68 m
Short25
Mw=8.67
NRMSE=0.661
Peak slip=16.38 m
ADK
UNA
CRC
SFP
ALM
SMO
SDG
ARI
ANT
VAL
TAL
MID
WAK
HON
HIL
APR
PAG
HAK
HAC
KUJ
MIY
AYU
ISH
KSM
0 2 4
Hour since Arrival

Fig. S6 (cont.).

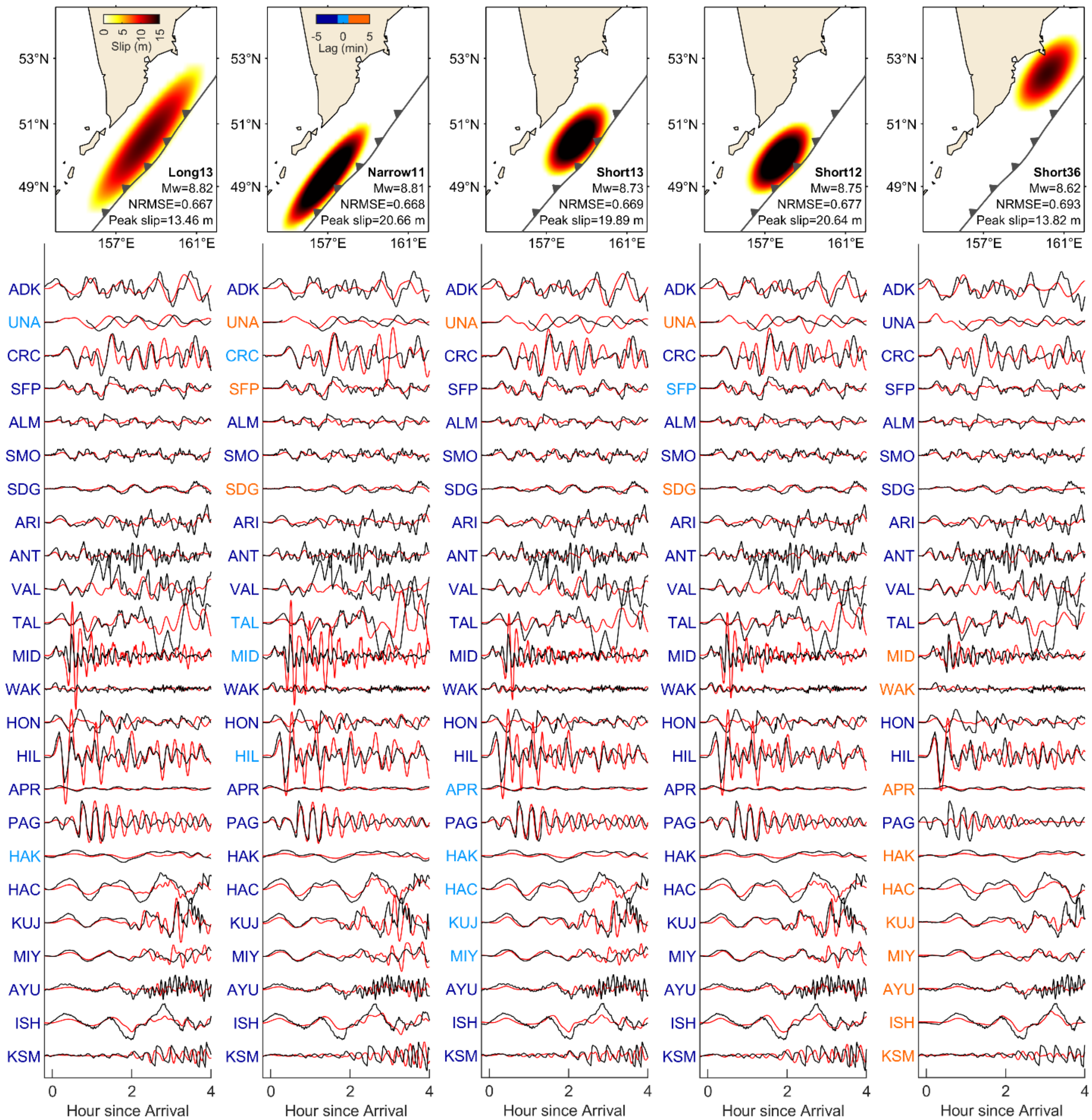

Slip (m)
Lag (min)
Long13
Mw=8.82
NRMSE=0.667
Peak slip=13.46 m
Narrow11
Mw=8.81
NRMSE=0.668
Peak slip=20.66 m
Short13
Mw=8.73
NRMSE=0.669
Peak slip=19.89 m
Short12
Mw=8.75
NRMSE=0.677
Peak slip=20.64 m
Short36
Mw=8.62
NRMSE=0.693
Peak slip=13.82 m
53°N
51°N
49°N
157°E
161°E
ADK
UNA
CRC
SFP
ALM
SMO
SDG
ARI
ANT
VAL
TAL
MID
WAK
HON
HIL
APR
PAG
HAK
HAC
KUJ
MIY
AYU
ISH
KSM
Hour since Arrival

Fig. S6 (cont.).

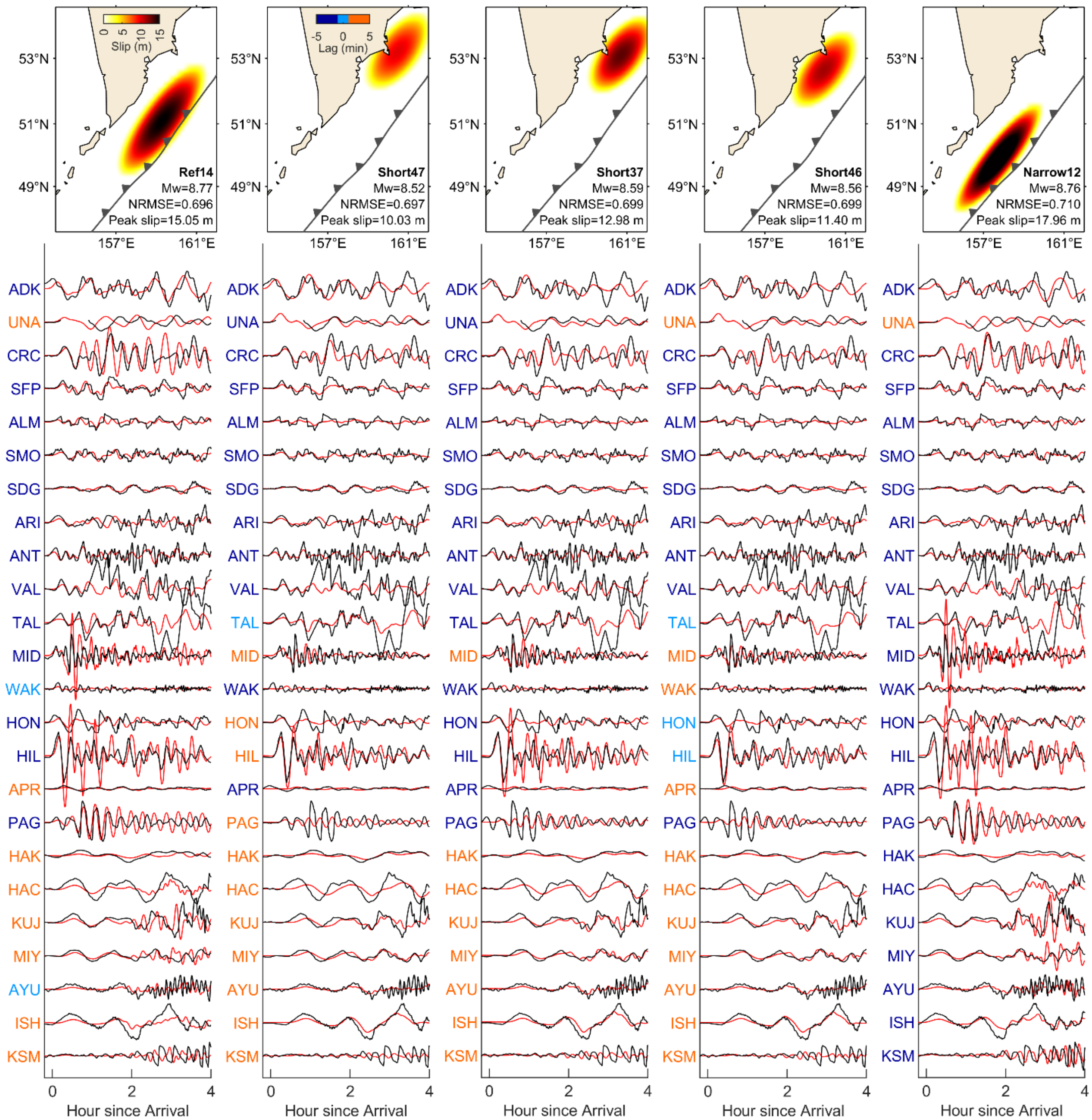

Slip (m)
0 5 10 15
Lag (min)
-5 0 5
53°N
51°N
49°N
157°E
161°E
Ref14
Mw=8.77
NRMSE=0.696
Peak slip=15.05 m
Short47
Mw=8.52
NRMSE=0.697
Peak slip=10.03 m
Short37
Mw=8.59
NRMSE=0.699
Peak slip=12.98 m
Short46
Mw=8.56
NRMSE=0.699
Peak slip=11.40 m
Narrow12
Mw=8.76
NRMSE=0.710
Peak slip=17.96 m
ADK
UNA
CRC
SFP
ALM
SMO
SDG
ARI
ANT
VAL
TAL
MID
WAK
HON
HIL
APR
PAG
HAK
HAC
KUJ
MIY
AYU
ISH
KSM
0 2 4
Hour since Arrival

Fig. S6 (cont.).

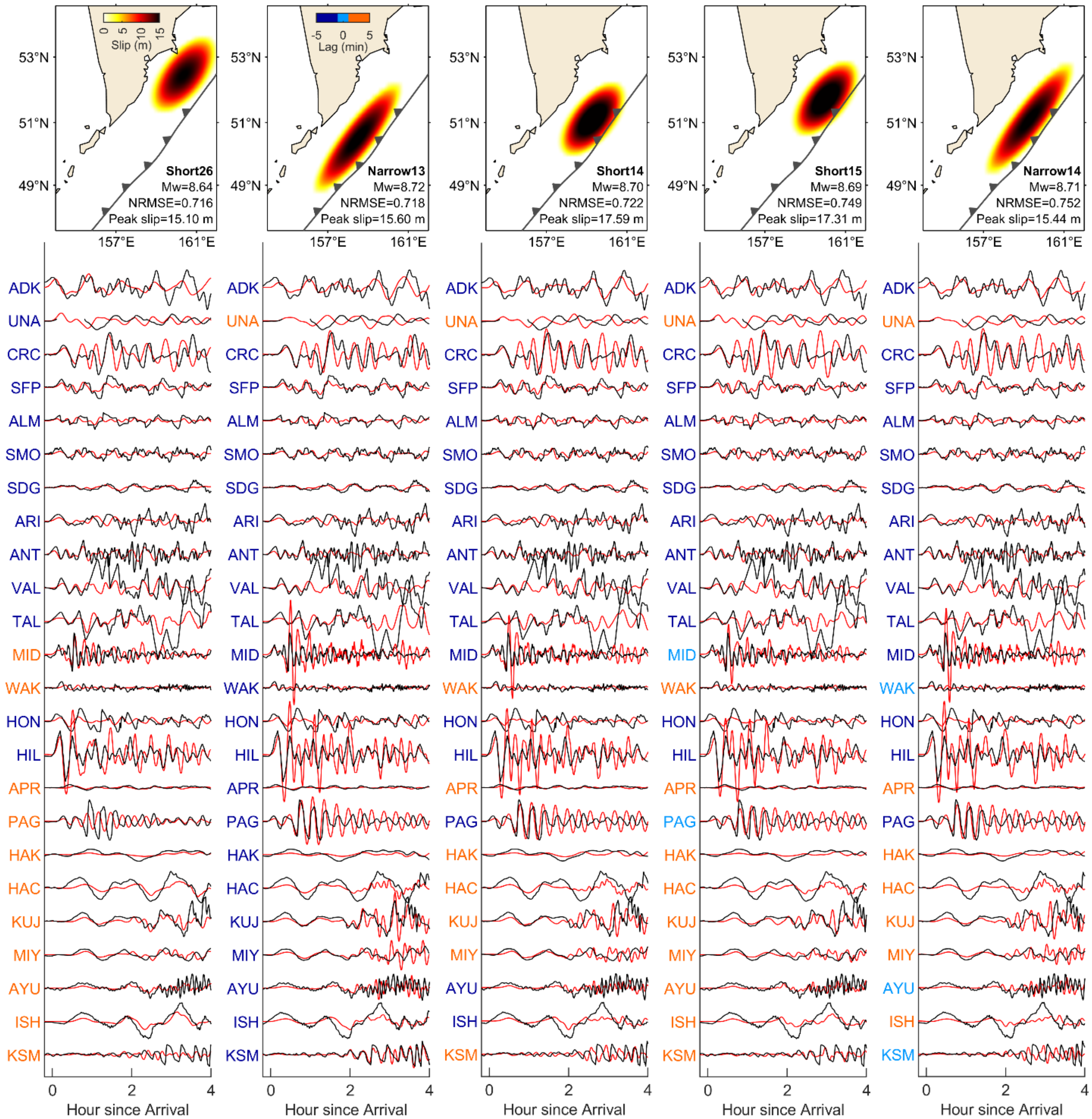

Slip (m)
0 5 10 15
Lag (min)
-5 0 5
53°N
51°N
49°N
157°E
161°E
Short26
Mw=8.64
NRMSE=0.716
Peak slip=15.10 m
Narrow13
Mw=8.72
NRMSE=0.718
Peak slip=15.60 m
Short14
Mw=8.70
NRMSE=0.722
Peak slip=17.59 m
Short15
Mw=8.69
NRMSE=0.749
Peak slip=17.31 m
Narrow14
Mw=8.71
NRMSE=0.752
Peak slip=15.44 m
ADK
UNA
CRC
SFP
ALM
SMO
SDG
ARI
ANT
VAL
TAL
MID
WAK
HON
HIL
APR
PAG
HAK
HAC
KUJ
MIY
AYU
ISH
KSM
0
2
4
Hour since Arrival

Fig. S6 (cont.).

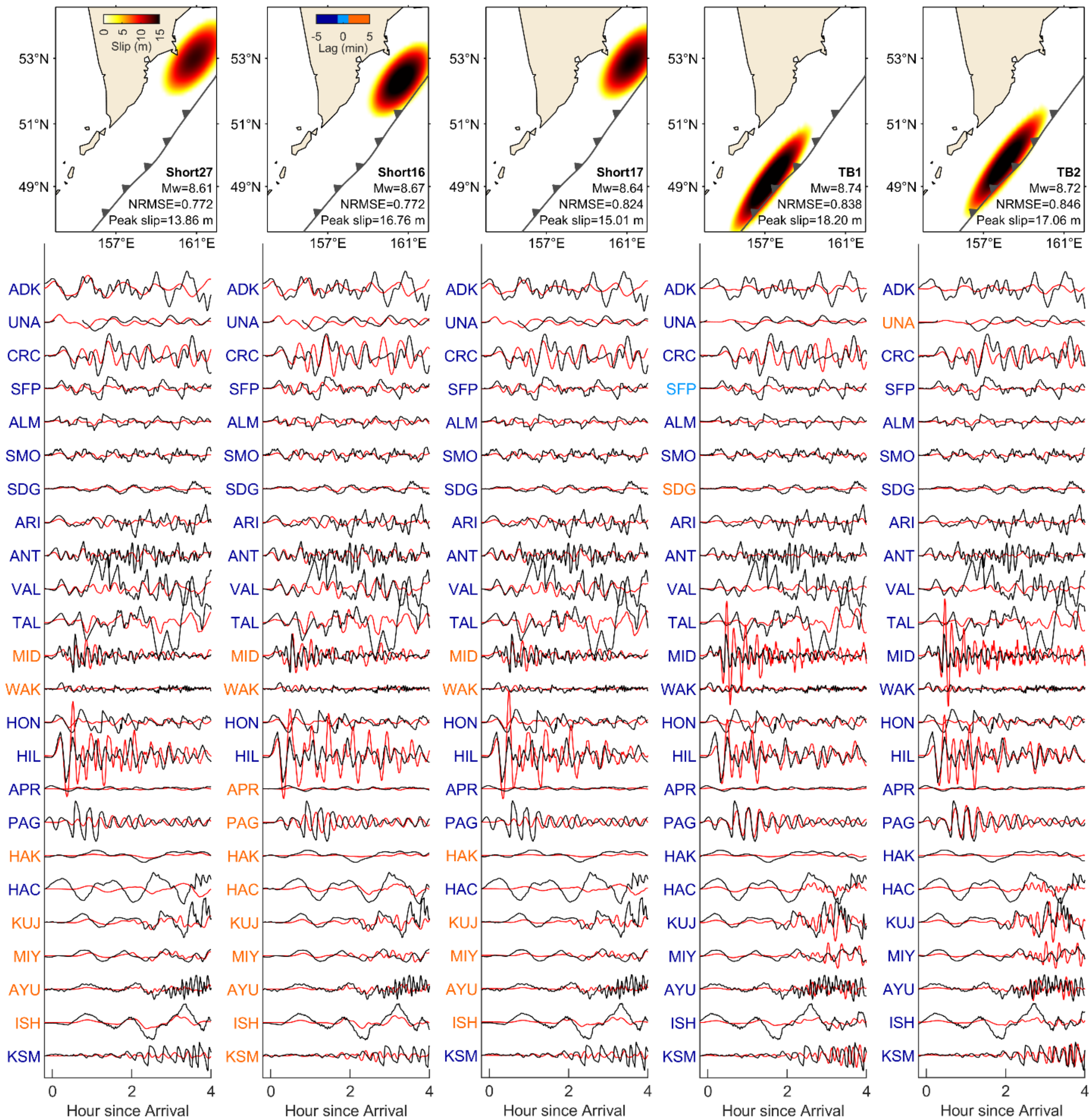

Slip (m)
Lag (min)
Short27
Mw=8.61
NRMSE=0.772
Peak slip=13.86 m
Short16
Mw=8.67
NRMSE=0.772
Peak slip=16.76 m
Short17
Mw=8.64
NRMSE=0.824
Peak slip=15.01 m
TB1
Mw=8.74
NRMSE=0.838
Peak slip=18.20 m
TB2
Mw=8.72
NRMSE=0.846
Peak slip=17.06 m
53°N
51°N
49°N
157°E
161°E
ADK
UNA
CRC
SFP
ALM
SMO
SDG
ARI
ANT
VAL
TAL
MID
WAK
HON
HIL
APR
PAG
HAK
HAC
KUJ
MIY
AYU
ISH
KSM
Hour since Arrival

Fig. S6 (cont.).

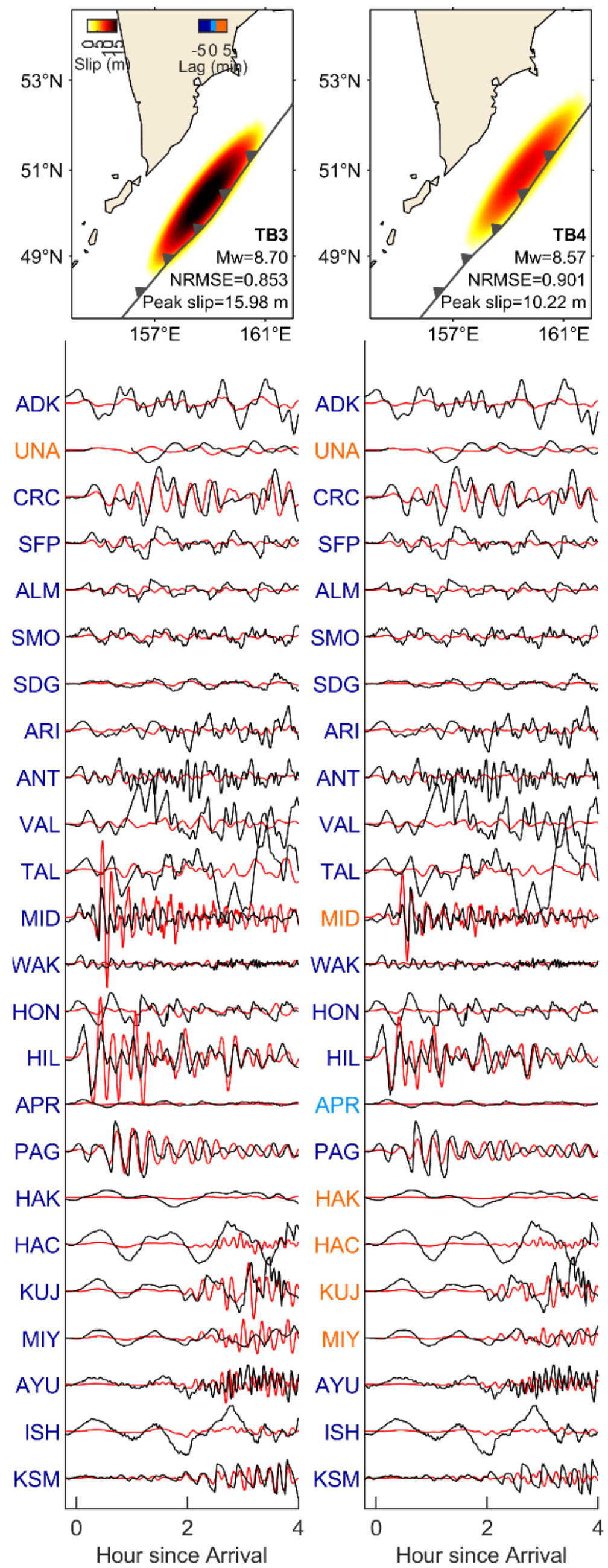
Slip (m)
Lag (min)
-50 5
53°N
51°N
49°N
TB3
Mw=8.70
NRMSE=0.853
Peak slip=15.98 m
157°E
161°E
TB4
Mw=8.57
NRMSE=0.901
Peak slip=10.22 m
ADK
UNA
CRC
SFP
ALM
SMO
SDG
ARI
ANT
VAL
TAL
MID
WAK
HON
HIL
APR
PAG
HAK
HAC
KUJ
MIY
AYU
ISH
KSM
0
2
4
Hour since Arrival

Fig. S6 (cont.).

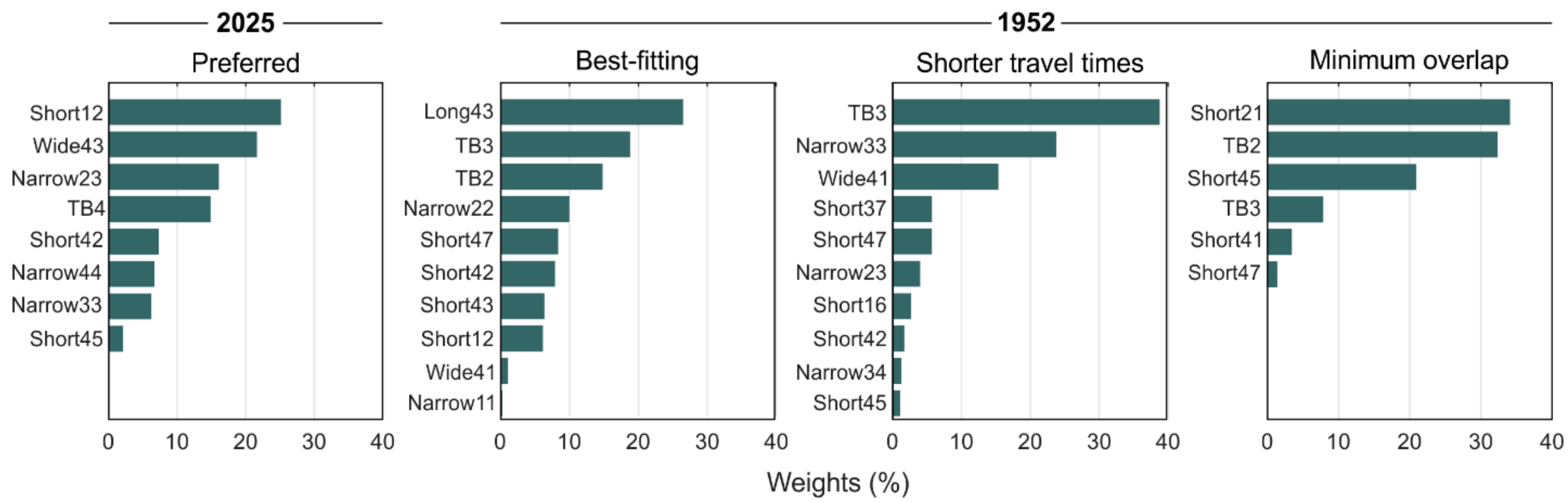

Fig. S7. Weights of the basic models that contribute to the weighted-average models shown in Fig. 4A–C and 4E. The following 13 basic models were also included in the weighted-average computation for the 1952 "Minimum overlap" model (Fig. 4E) but received negligible weights: Short25, Short26, Short27, Short15, Short16, Short17, Short11, Short35, Short36, Short37, Short46, TB1, and TB4.

Table S1. Tide-gauge stations used for comparison of the 1952 and 2025 Kamchatka tsunamis. List of 52 tide-gauge stations across the Pacific that are examined in this study, 24 of which are used for modeling as explained in the main text. The last column gives the observed travel-time difference ($\Delta t = T_{1952} - T_{2025}$) in minutes, as plotted in Figs. 1B and 2D. Names of the stations that were used in Fujii and Satake (21) are bold faced. Full references of data sources (a–i) are listed below the table.

Station name	Region	Location	Longitude	Latitude	Marigram access		Tide data	Δt
					1952	2025		
SEW	Alaska	Seward	210.683	-149.317	(c)	(f)	(g)	4
**UNA**	Alaska	Unalaska	193.470	-166.530	(c)	(f)	(g)	-11
**ADK**	Alaska	Adak	183.350	-176.650	(a)	(f)	(g)	-8
**SIT**	Alaska	Sitka	224.6583	57.0517	(c)	(f)	(g)	-3
YAK	Alaska	Yakutat	220.265	59.548	(c)	(f)	(g)	0
**PRU**	North America	Prince Rupert	229.483	-130.517	(b)	(f)	(g)	-4
**TOF**	North America	Tofino	234.083	-125.917	(b)	(f)	(g)	-4
**VIC**	North America	Victoria	236.617	-123.383	(a)	(f)	(g)	-5
AST	North America	Astoria	236.232	-123.768	(c)	(f)	(g)	-4
**CRC**	North America	Crescent City	235.817	-124.183	(b)	(f)	(g)	-6
**SFP**	North America	San Francisco	237.534	-122.466	(c)	(f)	(g)	-10
**ALM**	North America	Alameda	237.701	-122.299	(a)	(f)	(f)	-7
**SMO**	North America	Santa Monica	241.500	-118.500	(c)	(j)	(g)	-6
**LAX**	North America	Los Angeles	241.727	-118.273	(a)	(f)	(g)	-23
**LJO**	North America	La Jolla	242.740	-117.260	(a)	(f)	(g)	-11
SDG	North America	San Diego	242.826	-117.174	(a)	(f)	(g)	-2
ACA	North America	Acapulco	260.083	-99.917	(a)	(f)	(g)	-22
SAL	North America	Salina Cruz	264.817	-95.183	(a)	(f)	(g)	2
SJO	Central America	San José	269.199	-90.801	(c)	(f)	(g)	-4
LBS	Central America	La Libertad	270.683	-89.317	(c)	(f)	(f)	-15
PCR	Central America	Puntarenas	275.217	-84.783	(a)	(f)	(g)	-12
**LBE**	Sudamerica	La Libertad	279.083	-80.917	(a)	(f)	(g)	-2
**CLO**	Sudamerica	Callao	282.817	-77.183	(c)	(f)	(g)	-7
**ARI**	Sudamerica	Arica	289.677	-70.323	(b)	(f)	(g)	-4
**ANT**	Sudamerica	Antofagasta	289.595	-70.405	(c)	(f)	(g)	-3
**CAL**	Sudamerica	Caldera	289.175	-70.825	(c)	(f)	(g)	-5
**VAL**	Sudamerica	Valparaíso	288.373	-71.627	(c)	(f)	(g)	-20
**TAL**	Sudamerica	Talcahuano	286.894	-73.106	(c)	(f)	(f)	-25
MID	Pacific Islands	Midway	182.639	-177.361	(c)	(f)	(g)	-10
**HON**	Pacific Islands	Honolulu	202.135	-157.865	(c)	(f)	(g)	-5
**HIL**	Pacific Islands	Hilo	204.944	-155.056	(c)	(f)	(g)	-8
**WAK**	Pacific Islands	Wake	166.683	-193.317	(a)	(f)	(g)	-5
**APR**	Pacific Islands	Apra	144.657	-215.343	(a)	(f)	(f)	-5
YAP	Pacific Islands	Yap	138.083	-221.917	(a)	(f)	(g)	-10
**KWA**	Pacific Islands	Kwajalein	167.483	-192.517	(a)	(f)	(g)	7

**PAG**	Pacific Islands	Pago Pago	189.310	-170.690	(a)	(f)	(g)	-1
**KSH**	Japan	Kushiro	144.381	-215.619	(e)	(f)	(g)	4
**HAK**	Japan	Hakodate	140.708	-219.292	(e)	(f)	(g)	8
**AOM**	Japan	Aomori	140.734	-219.266	(d)	(i)	(h)	-7
HAC	Japan	Hachinohe	141.514	-218.486	(e)	(i)	(g)	-3
**KUJ**	Japan	Kuji	141.7965	40.192	(d)	(i)	(h)	-12
**MIY**	Japan	Miyako	141.963	-218.037	(d)	(i)	(h)	-1
ISH	Japan	Ishinomaki	141.338	-218.662	(d)	(i)	(h)	-6
AYU	Japan	Ayukawa	141.512	-218.488	(e)	(i)	(h)	-3
**ONA**	Japan	Onahama	140.901	-219.099	(e)	(i)	(h)	-1
KAT	Japan	Katsuura	140.249	-219.751	(e)	(i)	(h)	-25
**MER**	Japan	Mera	139.826	-220.174	(e)	(f)	(g)	-3
ITO	Japan	Ito	139.117	-220.883	(e)	(i)	(h)	-5
**TOB**	Japan	Toba	136.843	-223.157	(e)	(i)	(h)	-20
**KSM**	Japan	Kushimoto	135.778	-224.222	(e)	(f)	(g)	-2
UWA	Japan	Uwajima	132.550	-227.450	(e)	(i)	(h)	-25
HOS	Japan	Hososhima	131.670	-228.330	(e)	(i)	(g)	-3

(a) W. B. Zerbe, "The tsunami of November 4, 1952 as recorded at tide stations" (Special Publication No. 300, U.S. Coast and Geodetic Survey, 1953).
(b) NOAA National Centers for Environmental Information; NOAA Center for Tsunami Research, Archival and Discovery of November 4, 1952 Tsunami Event on Marigrams (NOAA National Centers for Environmental Information, 2017); https://www.ncei.noaa.gov/access/metadata/landing-page/bin/iso?id=gov.noaa.ngdc.mgg.hazards:19521104marigram.
(c) NOAA National Centers for Environmental Information, Archival and Discovery of Kamchatka November 4, 1952 Tsunami Event on Marigrams (NOAA National Centers for Environmental Information, 2025); https://www.ngdc.noaa.gov/hazel/view/hazards/tsunami/marigram-search/.
(d) Central Meteorological Observatory, "Investigation Report on the November 1952 Kamchatka Earthquake" in Quarterly Journal of Seismology (Japan Meteorological Agency, 1953), pp. 5–48; https://www.jma.go.jp/jma/kishou/books/kenshin/vol18p005.pdf.
(e) Earthquake Research Institute, Tsunami Waveform Database (The University of Tokyo, 2025); https://wwweic.eri.u-tokyo.ac.jp/tsunamidb/searchlist.html.
(f) Flanders Marine Institute (VLIZ); Intergovernmental Oceanographic Commission (IOC), Sea level station monitoring facility (VLIZ, 2025); DOI: 10.14284/482; https://www.ioc-sealevelmonitoring.org.
(g) University of Hawaii Sea Level Center (UHSLC), Hourly Sea Level Data for Station UH350A (School of Ocean and Earth Science and Technology, University of Hawaii at Manoa, 2025); https://uhslc.soest.hawaii.edu/data/?rq#uh350a.
(h) Japan Oceanographic Data Center (JODC), Tidal Data System – Sea Level Records (Japan Meteorological Agency, 2025); https://www.jodc.go.jp/vpage/tide.html.
(i) Geospatial Information Authority of Japan (GSI), Marigrams of the 1952 Kamchatka tsunami at multiple stations (Geospatial Information Authority of Japan, 2025); https://www.gsi.go.jp/kanshi/tide_furnish.html.
(j) Center for Operational Oceanographic Products and Services (CO-OPS), CO-OPS 1-minute Tsunami Water Level Data, NOAA National Centers for Environmental Information (2007); https://doi.org/10.7289/V59884XF

Data S1. (separate file)

Corrected records of 1952 tsunami.

REFERENCES

1. G. M. Sarkawi, L. Feng, J. W. Mc Caughey, A. J. Meltzner, S. Susilo, U. Muksin, A. Socquet, R. S. Oktari, S. P. Adi, R. Bürgmann, E. M. Hill, Insights into tectonic hazards since the 2004 Indian Ocean earthquake and tsunami. *Nat. Rev. Earth Environ.* **6**, 17–34 (2025).

2. T. Lay, S. P. Nishenko, Updated concepts of seismic gaps and asperities. *Proc. Natl. Acad. Sci. U.S.A.* **119**, e2216843119 (2022).

3. R. S. Stein, A. A. Barka, J. H. Dieterich, Progressive failure on the North Anatolian fault. *Geophys. J. Int.* **128**, 594–604 (1997).

4. S. Carena, A. M. Friedrich, A. Verdecchia, B. Kahle, S. Rieger, S. Kübler, Identification of source faults of large earthquakes in the Turkey–Syria border region between 1000 CE and the present, and their relevance for the 2023 M_w 7.8 Pazarcık earthquake. *Tectonics* **42**, e2023TC007890 (2023).

5. M. Ando, Source mechanisms and tectonic significance of historical earthquakes along the Nankai Trough, Japan. *Tectonophysics* **27**, 119–140 (1975).

6. S. P. Nishenko, Circum-Pacific seismic potential: 1989–1999. *Pure Appl. Geophys.* **135**, 169–259 (1991).

7. W. R. McCann, S. P. Nishenko, L. R. Sykes, J. Krause, Seismic gaps and plate tectonics. *Pure Appl. Geophys.* **117**, 1082–1147 (1979).

8. N. Hurukawa, P. Maung Maung, Two seismic gaps on the Sagaing Fault, Myanmar. *Geophys. Res. Lett.* **38**, L01310 (2011).

9. T. K. Pinegina, T. K. Pinegina, L. I. Bazanova, E. A. Zelenin, J. Bourgeois, A. I. Kozhurin, I. P. Medvedev, D. S. Vydrin, Holocene tsunamis in Avachinsky Bay. *Pure Appl. Geophys.* **175**, 1485–1506 (2018).

10. R. Bürgmann, M. G. Kogan, G. M. Steblov, G. Hilley, V. E. Levin, E. Apel, Interseismic coupling and asperity distribution along the Kamchatka subduction zone. *J. Geophys. Res.* **110**, B07405 (2005).

11. S. A. Fedotov, "On the seismic cycle, possibilities of quantitative seismic zonation, and long-term earthquake prediction," in *Seismic Zonation of the USSR* (Nauka, Moscow, 1968). pp. 121–150.

12. T. K. Pinegina, J. Bourgeois, L. I. Bazanova, E. A. Zelenin, S. P. Krasheninnikov, M. V. Portnyagin, Coseismic coastal subsidence associated with unusually wide rupture of prehistoric earthquakes on the Kamchatka subduction zone: A record in buried erosional scarps and tsunami deposits. *Quat. Sci. Rev.* **233**, 106171 (2020).

13. C. Liu, Y. Bai, T. Lay, P. He, Y. Wen, X. Xiong, T. Taymaz, Simple unilateral rupture of the great M_w 8.8 2025 Kamchatka earthquake. *Science* **391**, 812–817 (2026).

14. USGS, M8.8 Earthquake – East of the Kamchatka Peninsula (2025), https://earthquake.usgs.gov.

15. J. Kelleher, J. Savino, Distribution of seismicity before large earthquakes. *J. Geophys. Res.* **80**, 260–271 (1975).

16. T. K. Pinegina, A. Y. Ozerov, V. A. Tsvetkov, T. A. Kotenko, I. K. Mironov, J. Bourgeois, B. T. Mac Innes, A. V. Lander, D. V. Melnikov, L. V. Kotenko, A. I. Kozhurin, F. I. Batanov, A. L. Khomchanovsky, D. S. Vydrin, V. I. Frolov, D. R. Lukashenko, Tsunami from the M_w 8.8 Kamchatka earthquake of 29 July 2025 on the East Coast of Kamchatka and the North Kuril Islands. *Pure Appl. Geophys.* **183**, 1–25 (2025).

17. Y. Yamanaka, Y. Atobe, Y. Tsutsui, K. Okada, H. Takahashi, Y. Tanioka, Comparison of tsunamis following the 2025 and 1952 Kamchatka earthquakes. *Earth Planets Space* **77**, 193 (2025).

18. I. Sepúlveda, B. Nilsson, Y. Yu, M. Carvajal, M. Brandin, A.-A. Gabriel, D. Sandwell, SWOT detects dispersive tsunami tied to a near-trench source in the 2025 Kamchatka earthquake. *Science* **391**, 1368–1372 (2026).

19. B. T. MacInnes, R. Weiss, J. Bourgeois, T. K. Pinegina, Slip distribution of the 1952 Kamchatka earthquake. *Bull. Seismol. Soc. Am.* **100**, 1695–1709 (2010).

20. J. M. Johnson, K. Satake, Asperity distribution of the 1952 great Kamchatka earthquake. *Pure Appl. Geophys.* **154**, 541–553 (1999).

21. Y. Fujii, K. Satake, The slip distributions of the 1952 and 2025 Kamchatka earthquakes from tsunami waveforms recorded around the Pacific Ocean. *Geophys. Res. Lett.* **53**, e2025GL120682 (2026).

22. M. Carvajal, A. Gubler, G. Davies, K. Wang, T. Sun, P. A. Catalán, Large size of two 19th-century Chile–Peru earthquakes inferred from trans-Pacific tsunami records. *Geophys. Res. Lett.* **52**, e2024GL113849 (2025).

23. Intergovernmental Oceanographic Commission, *Manual on Sea Level Measurement and Interpretation* (UNESCO, 1985).

24. G. Davies, Tsunami variability from stochastic earthquake models: Tests against fourteen tsunamis at Australian tide gauges. *J. Geophys. Res.* **130**, e2025JB031949 (2025).

25. A. Williamson, D. Melgar, D. Rim, The effect of earthquake kinematics on tsunami propagation. *J. Geophys. Res.* **124**, 11639–11650 (2019).

26. A. Ben-Menahem, M. N. Toksöz, Source mechanism from spectrums of long-period surface waves: 2. The Kamchatka earthquake of November 4, 1952. *J. Geophys. Res.* **68**, 5207–5222 (1963).

27. K. Wang, Y. Zhu, E. Nissen, Z.-K. Shen, On the relevance of geodetic deformation rates. *Geophys. Res. Lett.* **48**, e2021GL093231 (2021).

28. F. Waldhauser, W. L. Ellsworth, A double-difference earthquake location algorithm: Method and application to the northern Hayward fault, California. *Bull. Seismol. Soc. Am.* **90**, 1353–1368 (2000).

29. F. Tomita, Re-evaluation of interseismic slip deficit rates. *Earth Planets Space* **78**, 41 (2026).

30. A. Ruiz-Angulo, D. Melgar, C. de Marez, A. Deniau, F. Nencioli, V. Hjörleifsdóttir, SWOT satellite observations and tsunami source model. *Seism. Rec.* **5**, 341–351 (2025).

31. R. E. Abercrombie, J. R. Rice, Can observations of earthquake scaling constrain slip weakening? *Geophys. J. Int.* **162**, 406–424 (2005).

32. H. Kanamori, L. Rivera, "Energy partitioning during an earthquake," in *Earthquakes: Radiated Energy and the Physics of Faulting* (AGU, 2006), pp. 3–13.

33. H. Luo, K. Wang, L. Feng, E. M. Hill, Interseismic secondary zone of subsidence during earthquake cycles in subduction zones. *Nat. Geosci.* **18**, 1027–1033 (2025).

34. K. Wang, Y. Hu, J. He, Deformation cycles of subduction earthquakes in a viscoelastic Earth. *Nature* **484**, 327–332 (2012).

35. F. Galetto, M. E. Pritchard, A. J. Hornby, E. Gazel, N. M. Mahowald, Spatial and temporal quantification of subaerial volcanism from 1980 to 2019. *Rev. Geophys.* **61**, e2022RG000783 (2023).

36. G. P. Avdeiko, A. A. Palueva, O. A. Khleborodova, Geodynamic conditions of volcanism and magma formation in the Kurile-Kamchatka island-arc system. *Petrology* **14**, 230–246 (2006).

37. A. V. Lander, M. N. Shapiro, The origin of the modern Kamchatka subduction zone. *Geophys. Monogr. Ser.* **172**, 57–64 (2007).

38. I. Koulakov, O. Bergal-Kuvikas, M. Voronova, A. Jakovlev, Deep magma sources beneath Central Kamchatka inferred from tomography. *Sci. Rep.* **15**, 16691 (2025).

39. M. Portnyagin, K. Hoernle, P. Plechov, N. Mironov, S. Khubunaya, Constraints on mantle melting and slab components in volcanic arcs from volatiles (H_2O, S, Cl, F) and trace elements in melt inclusions from the Kamchatka Arc. *Earth Planet. Sci. Lett.* **255**, 53–69 (2007).

40. B. F. Atwater, M. P. Tuttle, E. S. Schweig, C. M. Rubin, D. K. Yamaguchi, E. Hemphill-Haley, "Earthquake recurrence inferred from paleoseismology," in *The Quaternary Period in*

the United States, A. R. Gillespie, S. C. Porter, B. F. Atwater, Eds. (Elsevier Science, 2004), pp. 331–350.

41. C. Goldfinger, S. Galer, J. Beeson, T. Hamilton, B. Black, C. Romsos, J. Patton, C. H. Nelson, R. Hausmann, A. Morey, The importance of site selection, sediment supply, and hydrodynamics: A case study of submarine paleoseismology on the northern Cascadia margin, Washington USA. *Mar. Geol.* **384**, 4–46 (2017).

42. P.-L. Wang, S. E. Engelhart, K. Wang, A. D. Hawkes, B. P. Horton, A. R. Nelson, R. C. Witter, Heterogeneous rupture in the great Cascadia earthquake of 1700 inferred from coastal subsidence estimates. *J. Geophys. Res.* **118**, 2460–2473 (2013).

43. Y. Zhu, K. Wang, T. Sun, M. Carvajal, J. He, On dislocation modeling of megathrust tsunami sources. *J. Geophys. Res.* **130**, e2024JB030903 (2025).

44. G. P. Hayes, G. L. Moore, D. E. Portner, M. Hearne, H. Flamme, M. Furtney, G. M. Smoczyk, Slab2, a comprehensive subduction zone geometry model. *Science* **362**, 58–61 (2018).

45. Y. Okada, Surface deformation due to shear and tensile faults in a half-space. *Bull. Seismol. Soc. Am.* **75**, 1135–1154 (1985).

46. K. Wang, T. Sun, L. Brown, R. Hino, F. Tomita, M. Kido, T. Fujiwara, Learning from crustal deformation associated with the M9 2011 Tohoku-oki earthquake. *Geosphere* **14**, 552–571 (2018).

47. K. Kajiura, Tsunami source, energy and the directivity of wave radiation. *Bull. Earthq. Res. Inst. Univ. Tokyo* **48**, 835–869 (1970).

48. J. Macías, M. J. Castro, S. Ortega, C. Escalante, J. M. González-Vida, Performance benchmarking of tsunami-HySEA model for NTHMP's inundation mapping activities. *Pure Appl. Geophys.* **174**, 3147–3183 (2017).

49. GEBCO Bathymetric Compilation Group, *The GEBCO_2019 Grid: A Continuous Terrain Model of the Global Oceans and Land* (British Oceanographic Data Centre, National Oceanography Centre, 2019).

50. N. Chikasada, Global tsunami terrain model, NIED (2020); https://doi.org/10.17598/NIED.0021.

51. S. Watada, S. Kusumoto, K. Satake, Traveltime delay and initial phase reversal of distant tsunamis coupled with the self-gravitating elastic Earth. *J. Geophys. Res.* **119**, 4287–4310 (2014).

52. C. L. Lawson, R. J. Hanson, *Solving Least Squares Problems* (SIAM, 1995).

53. W. B. Zerbe, *The Tsunami of November 4, 1952 as Recorded at Tide Stations* (US Gov. Print. Office, 1953).
